# Four Ways to Fit an Ion Channel Model

**DOI:** 10.1016/j.bpj.2019.08.001

**Published:** 2019-08-06

**Authors:** Michael Clerx, Kylie A. Beattie, David J. Gavaghan, Gary R. Mirams

**Affiliations:** 1 Computational Biology & Health Informatics, Department of Computer Science, University of Oxford, Oxford, United Kingdom; 2 Centre for Mathematical Medicine & Biology, School of Mathematical Sciences, University of Nottingham, Nottingham, United Kingdom

## Abstract

Mathematical models of ionic currents are used to study the electrophysiology of the heart, brain, gut, and several other organs. Increasingly, these models are being used predictively in the clinic, for example, to predict the risks and results of genetic mutations, pharmacological treatments, or surgical procedures. These safety-critical applications depend on accurate characterization of the underlying ionic currents. Four different methods can be found in the literature to fit voltage-sensitive ion channel models to whole-cell current measurements: method 1, fitting model equations directly to time-constant, steady-state, and I-V summary curves; method 2, fitting by comparing simulated versions of these summary curves to their experimental counterparts; method 3, fitting to the current traces themselves from a range of protocols; and method 4, fitting to a single current trace from a short and rapidly fluctuating voltage-clamp protocol. We compare these methods using a set of experiments in which hERG1a current was measured in nine Chinese hamster ovary cells. In each cell, the same sequence of fitting protocols was applied, as well as an independent validation protocol. We show that methods 3 and 4 provide the best predictions on the independent validation set and that short, rapidly fluctuating protocols like that used in method 4 can replace much longer conventional protocols without loss of predictive ability. Although data for method 2 are most readily available from the literature, we find it performs poorly compared to methods 3 and 4 both in accuracy of predictions and computational efficiency. Our results demonstrate how novel experimental and computational approaches can improve the quality of model predictions in safety-critical applications.

## Significance

Mathematical models have been constructed to capture and share our understanding of the kinetics of ion channel currents for almost 70 years, and hundreds of models have been developed using a variety of techniques. We compare how well four of the main methods fit data, how reliable and efficient the process of fitting is, and how predictive the resulting models are for physiological situations. The most widely used traditional approaches based on current-voltage and time constant-voltage curves do not produce the most predictive models. Short, optimized experimental voltage-clamp protocols are as predictive as ones derived from traditional protocols, opening up possibilities for measuring ion channel kinetics faster, more accurately, and in single cells.

## Introduction

Computational models of ionic currents have been used to understand the formation of the cellular action potential (AP) (1, 2, 3), to simulate the effects of genetic mutations (4), and to study and predict the effects of pharmaceutical compounds that block various ion channels (5, 6). By fitting mathematical models to experimentally measured currents, we can learn about the kinetics of the underlying ion channels in healthy and in pathological situations. The need to understand voltage-sensitive ion channels’ function is widespread as they are key players in neuronal excitation (1), cardiac contraction (7), gastric function (8), insulin secretion (9), and several other aspects of physiology (10, 11). In cardiac arrhythmia research, models of ionic currents are routinely integrated into models of the AP and used to investigate the effects of genetic mutations, predict proarrhythmic risk in drug development, and inform clinical interventions (12, 13, 14). Such safety-critical applications depend on accurate characterization of the underlying ionic currents.

Several methods have been proposed to fit (parameterize) models of ionic currents, covering a range including pen-and-paper methods of the 1950s, detailed mathematical analysis specific to Hodgkin-Huxley models, and numerical “black-box” optimization. Data from several sources have been used, including whole-cell (aggregate) currents (1), single-channel currents (15), AP recordings (16), dynamic-clamp experiments (17), gating currents caused by the movement of charged parts of the ion channel proteins themselves (18), and measurements of fluorophores whose visibility varies with channel conformation (19).

In this manuscript, we focus on the common problem of fitting a set of predetermined model equations to voltage-dependent whole-cell ionic currents recorded under voltage clamp.

We describe four methods of fitting, each representative of a wider class of methods used in the electrophysiology literature. Our case study uses data from a previous study, in which currents were recorded at room temperature in nine Chinese hamster ovary (CHO) cells stably expressing hERG1a (20). Each cell was subjected to a series of training and validation protocols. All protocols were run on every cell so that we can apply all four methods to each cell, creating four model parameterizations, and then evaluate their ability to predict the current measured in the validation protocol. Finally, we discuss some of the reasons behind the observed differences in performance and compare the methods’ repeatability and time efficiency.

The first publication describing and fitting a model of ionic currents was by Hodgkin and Huxley (1). Using the idea that ionic conductance depends on some parts of the membrane being in one of two states, they postulated simple “gating” processes, each with a forward and a backward transition rate from which a time constant and a steady state could be deduced. They then set about designing protocols to measure (or approximate) these time constants and steady states at several voltages, fitted curves through these measurements, and used the resulting (phenomenological) equations to encode their transition rates. Similar methods of fitting the equations directly to experimentally derived points—using mathematical analysis, least-squares fitting, or manual adjustment—were employed by subsequent modelers (21, 22, 23, 24, 25, 26, 27, 28, 29, 30).

In this work, we describe a version of this method and refer to it as “method 1.” It is important to note that these methods rely on particular assumptions: 1) that the underlying biophysics is accurately described by one or more independent gating processes, each with their own steady-state and time-constant variables; and 2) that an experimental method and analysis procedure exists that will yield accurate values for these variables throughout a range of physiologically relevant voltages. The first assumption underlies all Hodgkin-Huxley-style models and any Markov models that have an equivalent Hodgkin-Huxley formulation. (Hodgkin-Huxley models are a subset of a more general class of Markov models. See, for example, Beattie et al. (20), which presents the equivalent Markov formulation of the Hodgkin-Huxley model used in this study, or Rudy and Silva (3), which provides examples of Markov models with and without Hodgkin-Huxley counterparts.) Testing whether the second assumption is violated can be done using simulated experiments, but proving it holds is challenging. A detailed mathematical analysis of two protocols was performed by Beaumont et al. (31), who showed that steady states can be obtained from peak currents only if there is a large difference in the time constants of the system, and that once these are known, the time constants can be estimated using time-to-peak measurements or by fitting exponential curves. In a follow-up study, they presented an iterative procedure to estimate the steady states for systems with more similar (“nonseparable”) time constants (32). In Wang and Beaumont (33), this analysis was taken further, and a method was derived to estimate steady-state equations and time constants simultaneously and further improve the results (the idea to fit both parameters together had also been used previously by Ebihara and Johnson (34)). Willms et al. (35) provided another mathematical analysis of fitting two-gate Hodgkin-Huxley models and concluded that separate estimation of time constants and steady states (“the disjoint method”) can lead to poor results. A further critique of method 1 was given by Lee et al. (36), who used mathematical analysis and simulation to point out errors arising for nonseparable time constants, and introduced methods aimed to combat this effect.

Despite these critiques, method 1 remains highly popular due of its simplicity. Estimates for time constants and steady states at different voltages are easily obtainable from the literature, which is a distinct advantage when multiple currents must be considered, e.g., when creating full AP models (7, 37, 38, 39).

An alternative to method 1, but using the same data points, is to simulate the voltage protocols and the subsequent analysis to obtain a set of simulated steady states and time constants to match the experimental ones. The parameters used in the simulation can then be updated in an iterative fashion (manually or using numerical optimization) until the simulated values match the experimental ones. This procedure allows both Hodgkin-Huxley and more generic Markov models to be fitted to published time-constant and steady-state data (although a unique fit is not guaranteed). If the modeler uses the same analysis method that was applied to the experimental data, any imperfections in the approximations of the steady states and time constants are replicated in the simulation so that good results may be obtainable even when method 1 approximations are poor (e.g., if time constants are nonseparable). In this manuscript, we use the name “method 2” to describe a version of this method based on numerical optimization.

Like method 1, method 2 is prevalent in the AP-modeling literature (40, 41, 42, 43, 44, 45, 46, 47). Further users include Moreno et al. (48), who mention the prevalence of published summary curve data as a reason to use method 2 rather than direct fits to current traces, as well as Perissinotti et al. (49), who provide a software package for performing method 2 optimizations. A tool by Teed and Silva (50) similarly implements a method 2 but also varies model structure when searching. Rather than manually tuning model parameters, several optimization algorithms have been applied to method 2, including simulated annealing (50), a Davidon-Fletcher-Powell optimizer (51), subspace trust region methods (45), and the Nelder-Mead simplex method (45, 48).

Instead of fitting to processed “summary data” (time constants and steady states), we can also fit simulated currents directly to measured currents from the same protocols: in this manuscript we term this “method 3.” Like method 2, method 3 is applicable to Markov-style models. Because method 3 does not require calculation of time constants and steady states, it is insensitive to errors in this process and can be more computationally efficient. A downside is that full current traces are more difficult to obtain and require the experimenter to have published their findings in digital format rather than printed tables or summary graphs.

The applicability of method 3 to Markov models was exploited by Balser et al. (52), who used numerical optimization and whole-current fitting to find the parameters of a model describing a cardiac potassium current. Similarly, Irvine et al. (53) formulated a Markov model of the cardiac fast sodium current and fitted it using method 3 (although they used additional data sources beyond whole-cell currents). Several algorithms have been used for the optimization step in method 3, including the Levenberg-Marquardt algorithm (52, 54), Nelder-Mead (52), simulated annealing (53), differential evolution (55), and genetic algorithms (56, 57), as well as hybrid methods combining multiple algorithms (58). Willms et al. (35) used simulated data to show advantages of method 3 over method 1 and provided a software tool for method 3 fitting (59).

Several authors have also compared fitting methodologies using identifiability analysis. A working definition of practical parameter identifiability is that fitting a particular data set constrains the parameters of a model to a small region of parameter space. As such, identifiability can be used as a tool to compare model complexity, fitting algorithms, and protocol designs. An extensive analysis of parameter estimation and identifiability for Markov models of ligand-gated channels was given by Milescu et al. (60), while Fink and Noble (61) used a sensitivity-based method to detect identifiability issues in published models and optimize voltage protocols. Csercsik et al. (62) investigated a procedure falling somewhere between methods 1 and 3, in which the parameters underlying voltage-dependence of steady states were determined, but time constants were fitted separately for different voltages. Walch and Eisenberg (63) considered the problem of estimating time constants and steady states as scalars for several voltages independently and concluded that in this case, only the time constants were identifiable. As Csercsik et al. (62) point out, full identifiability can be obtained by using voltage-dependent functions for the steady-state and time-constant equations.

In method 3, we do not calculate time constants or steady states, but we still use protocols designed specifically to estimate them. A next natural step, then, is to reconsider the established protocols and to ask what type of protocol would work best for method 3. We define “method 4” as the process of fitting directly to currents from a single protocol designed to characterize multiple aspects of current kinetics at once.

Perhaps the first demonstration of method 4 was by Millonas and Hanck (64), who fit a Markov model of a cardiac sodium current using a protocol consisting of random fluctuations between two voltages (“dichotomous noise”). This method was later applied to design protocols specifically to differentiate between competing models (65, 66, 67). A brief mention also occurs in Gurkiewicz and Korngreen (56), which discusses the benefits of method 3 for semiautomatic “high-throughput” construction of ionic current models and notes that it could be applied to “nonstandard protocols.” Fink and Noble (61) used identifiability analysis and simulation to show that standard voltage protocols can be considerably shortened while still being sufficient to parameterize various models. Importantly, they showed that protocols can be created that provide information on parameters for multiple cardiac currents so that it may be possible to formulate protocols without extensive preknowledge of the system (as is typical for standard protocols) and that protocols might be designed that are robust to changes induced by, e.g., drugs or mutations. Subsequent work by Clerx et al. (68) showed that the identifiability analysis employed by Fink and Noble (61) could be extended to take into account experimental errors. Finally, a study by Beattie et al. (20) proposed and tested a protocol design based on sinusoidal voltage clamps. Their work showed that a model could reliably be fit to the resulting current measurements and that models created this way outperformed published models in predicting the response to conventional voltage-clamp protocols and an AP waveform protocol.

Having discussed the four methods, we should point out that many studies do not stick to using a single method but use different approaches for different model parameters (69). In the remainder of this work, we formulate the four methods that characterize the approaches discussed above and evaluate their performance on a previously published experimental data set.

### Terminology and notation

Concepts such as “the time constant of activation” can be interpreted either as a property of a (macroscopic) current through the cell membrane or as a property of (microscopic) transitions of individual channels. Similarly, they can be seen either as the results of a particular set of (whole-cell) experiments or as model variables that may or may not have a physical counterpart. In this manuscript, we will use terms such as “model time constant” and “experimental time constant” to distinguish between model variables and experimentally derived values where necessary. When using mathematical symbols, we will use a tilde notation to denote experimentally derived values (e.g., ${\tilde{\tau }}_{a}$ for the experimental time constant of activation) and a plain notation for model variables (e.g., *τ*_*a*_ for the model time constant of activation). When referring to a vector of values at different voltages, we use boldface symbols (e.g., ***τ***_*a*_ and ${\tilde{\tau }}_{a}$). Finally, when referring to the full set of experimentally derived measures, we have tried to consistently use the term “summary curves” (based on the notion of a collection of “summary statistics” or “biomarkers”).

## Materials and Methods

### Current model

To model the hERG current, we use a two-gate Hodgkin-Huxley model found to provide excellent predictions by Beattie et al. (20) (described there in equivalent Markov model form). The current is defined by

(1) $$I_{\text{Kr}}=g_{\text{Kr}}\;a\;r\left(V-E_{K}\right),$$

where *g*_Kr_ is the maximal conductance, *a* and *r* are gating variables (defined below), *V* is the trans-membrane potential, and *E*_*K*_ is the reversal potential for potassium ions (note that although the measurements we used were from CHO cells expressing hERG1a, we use the shorthand terms *I*_Kr_ and *g*_Kr_ throughout this work). The Nernst equation was used to calculate a separate *E*_*K*_ value for each cell:

(2) $$E_{K}=\frac{RT}{F}\text{ln}\frac{{\left[{\text{K}}^{+}\right]}_{\text{o}}}{{\left[{\text{K}}^{+}\right]}_{\text{i}}},$$

where *R* is the gas constant, *F* is the Faraday constant, [K^+^]_o_ and [K^+^]_i_ are the bath (outside membrane) and pipette (inside membrane) concentrations of potassium ions, and *T* is the cell-specific temperature measured by Beattie et al. (20). In the nine cells used in this study, calculated values for *E*_*K*_ ranged from −88.45 to −88.30 mV with the precise value depending on the temperature at the time of recording.

The two processes represented independently by this Hodgkin-Huxley model are “activation” (with *a* representing the fraction of activated channels) and “inactivation” (with *r* representing the fraction of channels that have recovered from inactivation). Both variables *a* and *r* are dimensionless and in the range [0, 1]. Increases in *a* correspond to “activation” and decreases in *a* to “deactivation,” whereas decreases in *r* correspond to “inactivation” and increases in *r* to “recovery” from inactivation. (Note that the independence assumption means that—somewhat confusingly but perhaps appropriately—channels can be both “activated” and “inactivated” at the same time, because the opposite of activation is called deactivation and the opposite of inactivation is recovery.) We can represent the model as two independent gating reactions

(3) $$\left(1-a\right)\overset{k_{1}}{\underset{k_{2}}{⇌}}a\;\;\;\;r\overset{k_{3}}{\underset{k_{4}}{⇌}}\left(1-r\right),$$

where *a* is the fraction of channels in the activated state, (1 − *a*) is the fraction in the deactivated state, *r* is the recovered fraction, (1 − *r*) the inactivated fraction, and *k*_1_ to *k*_4_ are the voltage-dependent transition rates. The ordinary differential equations governing *a* and *r* are then derived with mass-action kinetics and can be written in the form

(4) $$\frac{\text{d}a}{\text{d}t}=\frac{a_{\infty }-a}{\tau_{a}},\;\;\;\;\frac{\text{d}r}{\text{d}t}=\frac{r_{\infty }-r}{\tau_{r}},$$

where *a*_∞_ and *r*_∞_ denote the model’s voltage-dependent steady states and *τ*_*a*_ and *τ*_*r*_ denote its voltage-dependent time constants, defined in terms of the transition rates as

(5) $$\tau_{a}=1/\left(k_{1}+k_{2}\right),\;\;\;\;\tau_{r}=1/\left(k_{3}+k_{4}\right),$$

(6) $$a_{\infty }=k_{1}\tau_{a},\;\;\;\;\;\;\;\;\;\;\;\;\;r_{\infty }=k_{4}\tau_{r}.$$

The voltage dependencies of the transition rates are defined using an Eyring-derived exponential formulation (53, 70, 71, 72) as

(7) $$k_{1}=p_{1}\text{exp}\left(p_{2}V\right),k_{3}=p_{5}\text{exp}\left(p_{6}V\right),$$

(8) $$k_{2}=p_{3}\text{exp}\left(-p_{4}V\right),k_{4}=p_{7}\text{exp}\left(-p_{8}V\right).$$

The model parameters to be inferred are therefore the kinetic parameters *p*_1_, *p*_2_, …, *p*_8_ and the conductance *p*_9_ = *g*_Kr_. All model parameters are taken to be strictly positive: *p*_*i*_ > 0 for *i* ∈ 1, 2, …, 9.

### Experimental methods

The experimental data used in this study are taken from Beattie et al. (20). In short, manual patch-clamp recordings were performed at room temperature (between 21 and 22°C) in CHO cells stably expressing hERG1a (which encodes the *α* subunit of the channel carrying *I*_Kr_). Recordings were taken from nine cells and seven protocols in each cell (we will refer to these as cells #1 to #9 and Pr1 to Pr7, with the numbering matching the original publication). After the final protocol was completed, the *I*_Kr_ blocker dofetilide was washed in, and all protocols were repeated. Each cell’s data was then postprocessed by first leak-correcting the signals recorded both in the presence and absence of dofetilide and then subtracting the *I*_Kr_-blocked signal from the unblocked one to remove any endogenous currents. For this study, we used the already leak-corrected and dofetilide-subtracted data as published on https://github.com/mirams/sine-wave. The first protocol, Pr1, did not elicit strong currents in any of the cells and so was not used in this study.

Following Beattie et al. (20), capacitance artifacts were removed from the experimental data by discarding the first 5 ms after each discontinuous voltage step. To obtain similar results from simulated protocols, the same filtering was applied to all simulated data.

**Figure 1:**
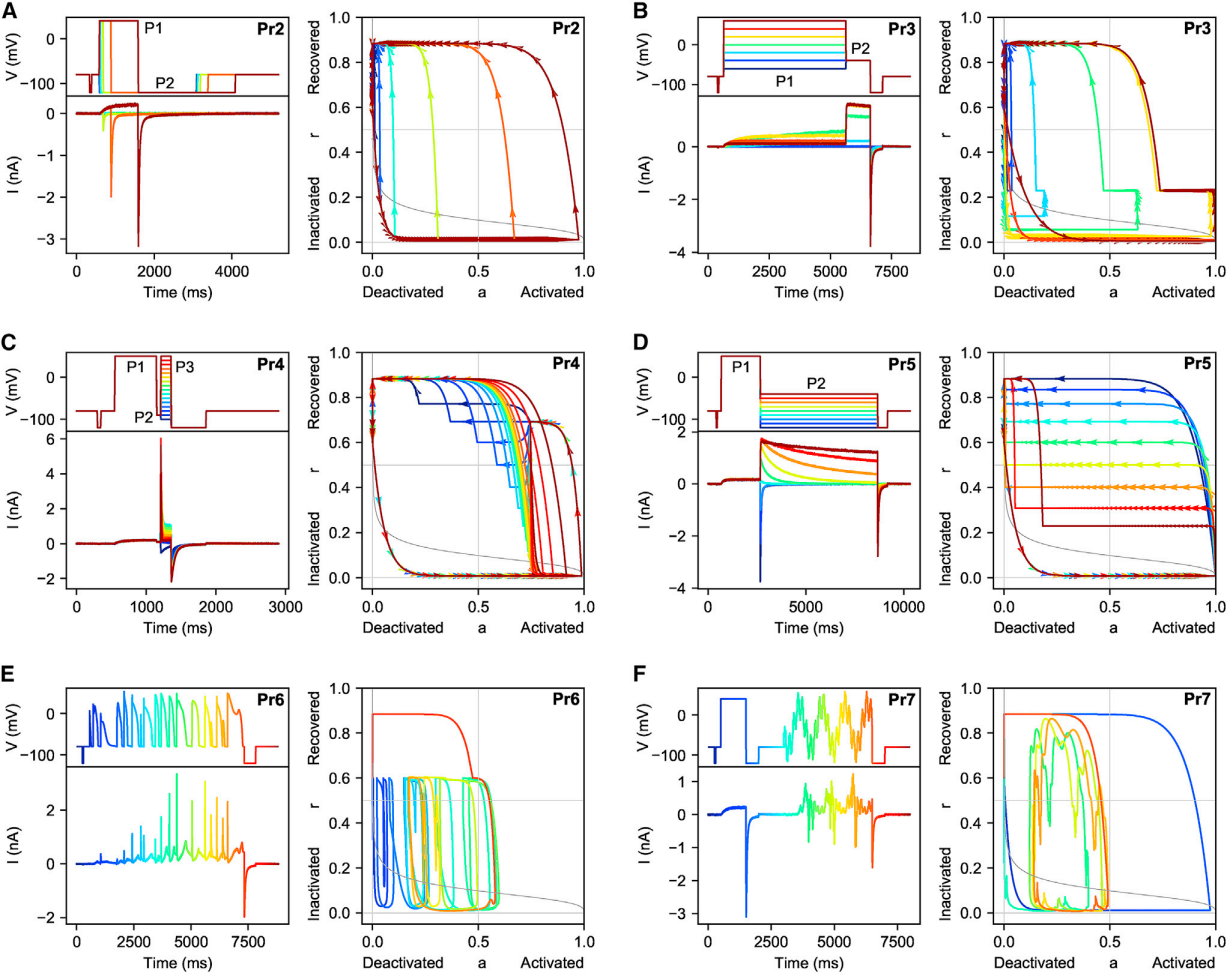
Voltage protocols, currents measured in cell #5, and simulated phase diagrams for all six protocols (Pr2–7) used in this study. Simulations for the phase diagrams were performed with the cell #5 parameters obtained in Beattie et al. (20). (*A*) Pr2 is used to measure the experimental time constant of activation. We show the voltage-step protocol (*top left*), the resulting current as measured in cell #5 (*lower left*), and a phase plane diagram (*right*). The protocol is repeated six times, with an increasing duration of the P1 step for each repeat. This is indicated in the plots by using a different color for each repeat. (*B*) Pr3 is used to measure the experimental steady state activation curve. It is repeated seven times, with a different voltage for the P1 step at each repeat. (*C*) Pr4 is used to measure experimental time constants of inactivation; it repeats 16 times with a different voltage for step P3. (*D*) Pr5 is used to measure experimental time constants of activation and inactivation, as well as steady-state inactivation. It has nine repeats with a different voltage for step P2. (*E*) Pr6 consists of several (healthy and pathological) AP wave forms and was used as an independent validation data set in this study. The colors in this plot indicate time so that the voltage and current traces can be related to the phase-plane trajectory on the right. (*F*) Pr7 is the sine wave protocol introduced by Beattie et al. (20). As in (*E*), color is used to indicate time information. To see this figure in color, go online.

The six protocols used in this study are shown in Fig. 1. The first four, Pr2–5, are adaptations of common step protocols used to characterize *I*_Kr_. Specifically, Pr2 is used to estimate a single time constant of activation (for *V* = +40 mV), Pr3 is used to estimate the steady state of activation, Pr4 is used to estimate time constants of inactivation, and Pr5 provides data about both time constants, as well as the steady state of inactivation. Pr7 is a novel sinusoidal protocol introduced by Beattie et al. (20) and is intended to provide the same information in a much shorter time. It consists of an initial step to +40 mV, designed to trigger a large current, followed by a section consisting of the sum of three sine waves. Finally, Pr6 is a collection of AP wave forms, representing the membrane potential under physiological and pathological conditions. As in Beattie et al. (20), we used Pr6 as a validation protocol while either Pr7 or the set Pr2–5 were used for fitting. Note that the full set of protocols was run on every cell.

A detailed description of all protocols and the associated analysis methods is given in Supporting Materials and Methods S1, Section S1. In analyzing these protocols, we found it useful to perform simulations (using the parameters obtained by Beattie et al. (20)) and plot the model state in a two-dimensional phase diagram as shown in Fig. 1. A guide to using these diagrams for analysis is provided in Supporting Materials and Methods, Section S1.1.

**Figure 2:**
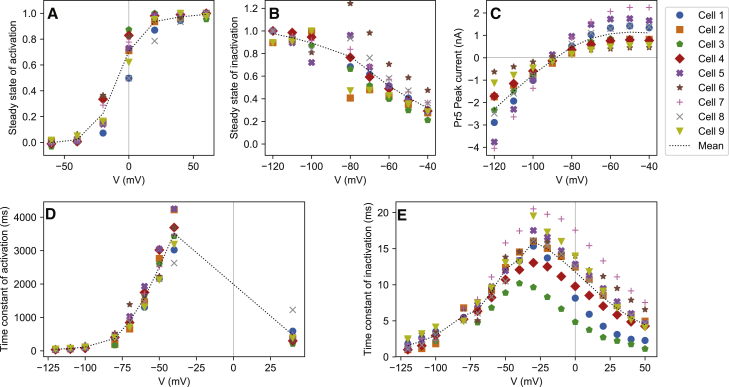
Summary curves calculated from the voltage-step protocols Pr2–5 for all nine cells. The mean for all cells is indicated with a dotted line. Note how the general trend, not always obvious from the single-cell results, is clearly captured by the line connecting the means. (*A*) The experimental activation curve ***ã***_∞_ derived from Pr3 is shown. (*B*) The experimental steady state of inactivation ${\tilde{r}}_{\infty }$ derived from Pr5. (*C*) The IV curve (peak current during the P2 step of Pr5) plotted against the P2 voltage. (*D*) The experimental time constant of activation ${\tilde{\tau }}_{a}$. Values for *V* < 0 mV were estimated from Pr5, the single value at 40 mV from Pr2. (*E*) The experimental time constant of inactivation ${\tilde{\tau }}_{r}$. Values for *V* < −30 mV were estimated from Pr5, values for −40 mV and upwards from Pr4. To see this figure in color, go online.

The voltage-step protocols Pr2–5 can be used to derive a set of graphs that characterize the current, shown in Fig. 2 for all nine cells used in this study. They are commonly referred to as the “steady state of activation” (Fig. 2 *A*), “steady state of inactivation” (Fig. 2 *B*), “[Peak/tail] IV curve” (Fig. 2 *C*), “time constant of activation” (Fig. 2 *D*), and “time constant of inactivation” (Fig. 2 *E*). In the remainder of the work, we will refer to the curves in Fig. 2 as the summary curves.

### Four ways of fitting

#### Method 1: Fitting model equations directly to summary curves

We now describe the four methods of fitting in detail. In method 1, we write out equations for the model variables *a*_∞_(*V*), *r*_∞_(*V*), *τ*_*a*_(*V*), and *τ*_*r*_(*V*) in terms of the parameters *p*_1_, *p*_2_, …, *p*_8_ and then try to fit these directly to the experimental summary curves ***ã***_∞_, ${\tilde{r}}_{\infty },{\tilde{\tau }}_{a}$, and ${\tilde{\tau }}_{r}$. The procedure for doing so closely follows that of Hodgkin and Huxley, and in fact, the procedure for finding *p*_1_ to *p*_8_ can be done entirely with pen and paper, and only a single simulation is needed to estimate the ninth parameter *g*_Kr_. First, we focus on the steady state of activation (Eq. 6) and substitute in the parameters *p*_1_ to *p*_4_:

(9) $$a_{\infty }\left(V\right)=k_{1}\tau_{a}=\frac{k_{1}}{k_{1}+k_{2}}=\frac{1}{1+k_{2}/k_{1}},$$

(10) $$={\left[1+\frac{p_{3}e^{-p_{4}V}}{p_{1}e^{p_{2}V}}\right]}^{-1},$$

(11) $$={\left[1+e^{\left(\text{log}p_{3}-\text{log}p_{1}\right)-\left(p_{2}+p_{4}\right)V}\right]}^{-1}.$$

This can be rewritten as a “Boltzmann curve”

(12) $$a_{\infty }\left(V\right)=\frac{1}{1+e^{\left(s_{a}h_{a}-s_{a}V\right)}}=\frac{1}{1+e^{s_{a}\left(h_{a}-V\right)}},$$

where *h*_*a*_ is the “midpoint of activation” (the point at which *a*_∞_(*V* = *h*_*a*_) = 0.5) and *s*_*a*_ is the slope of the activation curve at *V* = *h*_*a*_. Assuming *ã*_∞_(*V*) ≈ *a*_∞_(*V*), values for *h*_*a*_ and *s*_*a*_ can then be obtained numerically (optimizing on sum of square error) or by plotting *ã*_∞_ vs. *V* and reading the values from the graph. At this stage we have an equation for *a*_∞_(*V*) and two constraints on the parameters *p*_1_, *p*_2_, *p*_3_, *p*_4_, namely

(13) $$s_{a}=p_{2}+p_{4},$$

(14) $$h_{a}=\frac{\log\;p_{3}-\log\;p_{1}}{s_{a}}.$$

Next, we rewrite Eq. 6 to find

(15) $$a_{\infty }\left(V\right)/\tau_{a}\left(V\right)=k_{1}\left(V\right)=p_{1}e^{p_{2}V},$$

which allows us to infer values for *p*_1_ and *p*_2_ by plotting the quantity *a*_∞_/${\tilde{\tau }}_{a}$ vs. *V* (using the fitted equation for *a*_∞_(*V*) rather than the measured values ***ã***_∞_ to reduce noise) and fitting an exponential curve. Note that because *a*_∞_ ≈ 0 for values below −60 mV, any data points for low voltages will contribute very little to the final fit. As a simple alternative, we can plot the quantity log(*a*_∞_/${\tilde{\tau }}_{a}$) and fit with a linear equation log(*p*_1_) + *p*_2_*V* (note that this is equivalent to doing a graphical fit using semilogarithmic graph paper); this provides us with more reliable values for *p*_1_ and *p*_2_, after which we can use *p*_4_ = *s*_*a*_ − *p*_2_ and $p_{3}=p_{1}e^{s_{a}h_{a}}$ to find the remaining activation parameters.

Using a similar procedure (but with a slight change in signs), we plot the logarithm of $k_{4}\left(V\right)=r_{\infty }/{\tilde{\tau }}_{r}=p_{7}e^{-p_{8}V}$, fit to find *p*_7_ and *p*_8_, and then use *p*_6_ = −*s*_*r*_ − *p*_8_ and $p_{5}=p_{7}e^{s_{r}h_{r}}$ to find all four inactivation parameters.

Finally, we find a value for the conductance parameter *g*_Kr_ by performing a simulation of Pr5, deriving an IV curve, and then calculating the scaling factor that minimizes the sum-of-squares error between the simulated and experimental IV curves.

#### Quantifying goodness of fit

To evaluate the goodness of fit from method 1, we derive an error function and evaluate it with the obtained parameter values. We write ***θ*** = {*p*_1_, *p*_2_, …, *p*_9_} for the parameters and introduce symbols representing the experimental and measured data sets. To denote a cell’s set of experimentally approximated midpoints of activation, we use ${\tilde{a}}_{\infty }^{\text{cell}}$ while $a_{\infty }^{\text{model}}$ is used to indicate the value of the model variable (*a*_∞_), evaluated at the same voltages. Similar notation is used for the midpoint of inactivation and both time constants. Next, we define the root mean-squared error (RMSE) between two data sets ***x*** and ***y*** as

(16) $$R\left(x,y\right)=\sqrt{\frac{1}{n}\overset{n}{\sum_{i=1}}{\left(x_{i}-y_{i}\right)}^{2}},$$

where both data sets have equal length *n*. Using this notation, we can write the RMSE between experimental results ${\tilde{a}}_{\infty }^{\text{cell}}$ and model values $a_{\infty }^{\text{model}}$ as $R\left({\tilde{a}}_{\infty }^{\text{cell}},a_{\infty }^{\text{model}}\right)$. With this notation, we can now define the error criterion as a weighted sum of RMSEs:

(17) $$E_{\text{M}1}\left(\theta \right)=R\left({\tilde{a}}_{\infty }^{\text{cell}},a_{\infty }^{\text{model}}\right)+R\left({\tilde{r}}_{\infty }^{\text{cell}},r_{\infty }^{\text{model}}\right)+\frac{R\left({\tilde{\tau }}_{a}^{\text{cell}},\tau_{a}^{\text{model}}\right)}{\text{max}{\tilde{\tau }}_{a}^{\text{cell}}}+\frac{R\left({\tilde{\tau }}_{r}^{\text{cell}},\tau_{r}^{\text{model}}\right)}{\text{max}{\tilde{\tau }}_{r}^{\text{cell}}},$$

where $n_{a_{\infty }}=7$, $n_{r_{\infty }}=8$, $n_{\tau_{a}}=9$, and $n_{\tau_{r}}=17$. Note that this is a cell-specific measure because both time-constant terms are normalized with respect to the largest value found in the cell data used. This weighting corrects for differences in the scaling of the four RMSEs, ensuring that none of them dominate in the end result. No normalization is needed for the steady states, which are already constrained to the range [0, 1]. In contrast to measures introduced below, *E*_M1_ is invariant with respect to conductance (*p*_9_). Finally, we note that an alternative method 1 could be created by using numerical optimization to minimize *E*_M1_; this is explored further in Supporting Materials and Methods, Section S3.6, but did not create a more predictive model than the method presented here.

#### Method 2: Fitting simulated summary curves

In method 2, we accept that the summary curves are imperfect approximations of the model variables, and so we base our fitting on simulated experiments of Pr2–5, analyzed using the same methods as for the experimental data to arrive at simulated versions of the summary statistic curves. This gives us two sets of summary curves, one simulated and one experimental, using which we can define an error measure that quantifies the goodness of fit. By varying the parameters and repeating the simulations, we can then find a set of parameters that minimizes this error.

To formulate the error measure, we again write ${\tilde{a}}_{\infty }^{\text{cell}}$ to denote a cell’s set of experimentally approximated midpoints of activation, and we introduce the notation ${\tilde{a}}_{\infty }^{\text{sim}}$ for its simulated counterpart (note that we use a tilde notation here to indicate that ${\tilde{a}}_{\infty }^{\text{sim}}$ is not a model variable but a result derived from a simulated experiment). In this measure, we use the IV curve rather than the steady state of inactivation because 1) it contains the same data points (albeit with a different scaling); 2) unlike ${\tilde{r}}_{\infty }^{\text{cell}}$, it does not suffer from numerical issues near *V* = *E*_*K*_ (see Supporting Materials and Methods, Section S1.5.1); and 3) it contains information about *g*_Kr_, which is lacking from the other summary curves. Similar symbols denote the remaining summary curves: experimental midpoint of inactivation $\left({\tilde{r}}_{\infty }^{\text{cell}}\right)$, time constant of activation $\left({\tilde{\tau }}_{a}^{\text{cell}}\right)$, time constant of inactivation $\left({\tilde{\tau }}_{r}^{\text{cell}}\right)$, and the IV curve from Pr5 (**IV**^cell^).

The error to minimize for each cell is defined as a weighted sum of RMSEs

(18) $$E_{\text{M}2}\left(\theta \right)=R\left({\tilde{a}}_{\infty }^{\text{cell}},{\tilde{a}}_{\infty }^{\text{sim}}\right)+\frac{R\left({\tilde{\tau }}_{a}^{\text{cell}},{\tilde{\tau }}_{a}^{\text{sim}}\right)}{\text{max}{\tilde{\tau }}_{a}^{\text{cell}}}+\frac{R\left({\tilde{\tau }}_{r}^{\text{cell}},{\tilde{\tau }}_{r}^{\text{sim}}\right)}{\text{max}{\tilde{\tau }}_{r}^{\text{cell}}}+\frac{R\left(IV^{\text{cell}},IV^{\text{sim}}\right)}{\text{max}\;IV^{\text{cell}}-\text{min}\;IV^{\text{cell}}}.$$

The number of data points was the same for each cell, with $n_{\tau_{a}}=9$, $n_{\tau_{r}}=17$, $n_{a_{\infty }}=7$, and *n*_*IV*_ = 9 (for a total of 42 cell-specific data points). As in *E*_M1_, weighting is used here to give every term equal influence.

#### Method 3: Fitting current traces from traditional protocols

In method 3, we forgo the summary-curve calculation altogether and simply perform “whole-trace fitting” on the currents resulting from the “traditional protocols” Pr2–5. Writing $I_{i}^{\text{cell}}$ for the current recorded from protocol *i*, and $I_{i}^{\text{sim}}$(***θ***) for the simulated current in protocol *i* with parameters ***θ***, we define the function to be optimized as a normalized RMSE:

(19) $$E_{\text{M}3}\left(\theta \right)=\overset{5}{\sum_{i=2}}\frac{R\left(I_{i}^{\text{cell}},I_{i}^{\text{sim}}\right)}{\text{max}\;I_{i}^{\text{cell}}-\text{min}\;I_{i}^{\text{cell}}}.$$

Note that the weighting here is not strictly necessary for the optimization procedure but is used to enable *E*_M3_ value comparisons between cells.

#### Method 4: Fitting current traces from an optimized protocol

In method 4, we define a similar normalized RMSE measure based on fitting only the current under the sinusoidal protocol, Pr7:

(20) $$E_{\text{M}4}\left(\theta \right)=\frac{R\left(I_{7}^{\text{cell}},I_{7}^{\text{sim}}\right)}{\text{max}\;I_{7}^{\text{cell}}-\text{min}\;I_{7}^{\text{cell}}}.$$

As in *E*_M3_, the weighting used here allows us to compare values between cells.

### Validation and cross comparison

To compare the results of the four fitting methods, we applied each method to each cell, resulting in four parameter sets per cell. Next, we simulated the AP waveform protocol (Pr6) with all parameter sets and compared these to the corresponding measurements. Note that Pr6 was not used in any of the fitting methods, so this constitutes an independent validation. The results were quantified using a normalized RMSE:

(21) $$E_{\text{AP}}\left(\theta \right)=\frac{R\left(I_{6}^{\text{cell}},I_{6}^{\text{sim}}\right)}{\text{max}\;I_{6}^{\text{cell}}-\text{min}\;I_{6}^{\text{cell}}},$$

where *R* is defined by Eq. 16, as before.

In addition to the independent validation, we performed cross-validation by testing how well models fit with one method could predict the fitting data used by the others. This was shown visually and was quantified by evaluating *E*_M1_, *E*_M2_, *E*_M3_, and *E*_M4_ on the best result found for each cell/method.

Multicell measures of the fitting methods’ performance were defined as the mean average of the error measure over all nine cells. Writing *E*_AP,*k*_ for the RMSE in cell *k* on the AP signal, we defined

(22) $$E_{\text{AP},\text{all}}=\frac{1}{9}\sum_{k=1}^{9}E_{\text{AP},k}.$$

Note that *E*_AP,*k*_ already contains a term to normalize the error with respect to the magnitude of the current in cell *k* so that no further weighting is required. Combined error measures for *E*_M1_ through to *E*_M4_ were defined in the same manner.

### Minimizing the error measures

Methods 2–4 all proceed by finding a parameter set that minimizes an error function. In previous work, we found that the global optimization algorithm CMA-ES (73) provided good fits for a range of models from the ion channel to cell scale (20, 74, 75, 76, 77), and was both fast and behaved reproducibly (returning the same answer when started from different initial guesses in the parameter space). For methods 2–4, we used a CMA-ES population size of 10 and halted the optimization only when the objective function changed by less than 10^−11^ per iteration for 200 successive iterations. Other important aspects of making our optimization strategy reliable were 1) placing physiologically inspired bounds on the parameter space, 2) searching (and choosing starting points) in a log-transformed space, 3) reducing solver tolerances to eliminate numerical noise in simulation output, and 4) testing reliability by running repeated fits from different starting points.

**Figure 3:**
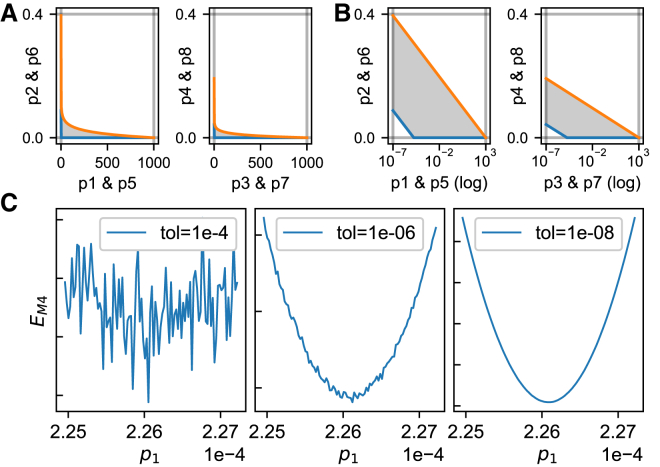
Boundaries and transforms on the parameter space and the effect of solver tolerances. (*A*) Constraining the transition rates *k*_1_–*k*_4_ (over physiologically relevant voltages) to relevant timescales creates a two-dimensional boundary on each parameter pair (Supporting Materials and Methods, Section S2.2). The gray region indicates valid samples, with an upper rate constraint in orange and a lower rate constraint in blue. The horizontal and vertical gray lines indicate the lower and upper bounds for the parameters *p*_1_–*p*_8_. (*B*) The same parameter constraints with a log transform on one of the parameters are shown. Note this increases the size of the feasible region for off-the-shelf optimizers that know nothing about the problem, and we later see the objective function becomes reasonably convex and symmetric under this transform (see Fig. 11). (*C*) Using default tolerances of adaptive solvers can lead to numerical noise in the function being optimized. This can be remedied by selecting lower tolerances. To see this figure in color, go online.

Constraints on the parameter space were set as in Beattie et al. (20) and included upper and lower bounds for the parameters *p*_1_ to *p*_9_ but also restricted the value of the reaction rates *k*_1_ to *k*_4_. The resulting boundaries are visualized in Fig. 3, *A* and *B*. Details of how the boundaries were defined are given in Supporting Materials and Methods, Section S2.2. To implement these constraints, points outside the boundaries were automatically assigned an error of ∞ during optimization.

Several studies (78, 79, 80) have found that for strictly positive parameters (i.e., for all nine parameters in our model), optimization performance can be improved by searching in a log-transformed parameter space. In at least some cases, this has been shown to turn nonconvex (i.e., hard) problems into simpler convex ones (81). For methods 2–4, we used a log transform on parameters *p*_1_, *p*_3_, *p*_5_, and *p*_7_. As Fig. 3 *B* shows, the boundaries on these parameters allowed them to vary over 10 orders of magnitude, so a log transform seemed most appropriate. The effects of this strategy are further explored in Supporting Materials and Methods, Section S2.4.

We used an adaptive step time solver for simulations of Pr6 and Pr7. As shown in Johnstone (82), simulating with lax tolerance settings can lead to (seemingly random) fluctuations in the error measure for nearby values of ***θ***, which has the effect of creating several local minima in otherwise smooth regions. To combat this, we set absolute and relative error tolerances of 10^−8^. This is shown in Fig. 3 *C*. For the voltage-step protocols Pr2–5, we circumvented the issue entirely by using the model’s analytical solution for fixed voltages to calculate the time course at each step.

When running the optimizations for methods 2–4, each fit was run several times from different starting points, sampled uniformly from within the transformed space shown in Fig. 3 *B*. Fifty repeats were run per cell for methods 3 and 4 while 80 repeats per cell were run for method 2. The best result (lowest error) was used as the final fit for each cell/method. We comment on the reliability of these fits below in the Reliability and Performance part of the Results.

We tested our methods by performing a study on synthetic data, described in Supporting Materials and Methods, Section S2.3, based on parameters given in Beattie et al. (20) and Gaussian noise comparable to the strongest noise encountered in our data set. In this in silico study, we saw that our methods reliably found a point near the known true solution, that solutions found by repeated fits had very similar parameter values, and that models fitted to one data set provided good predictions on the other data sets.

### Software and algorithms

Simulations were performed in Myokit (83), using CVODE (84) for Pr6 and Pr7 simulations with tolerances as described above and an analytical solver for Pr2–5. Further analysis was performed in Python 3.7 using NumPy/SciPy (85). When deriving experimental time constants, fits to exponential curves were performed using the Nelder-Mead downhill simplex algorithm implemented in SciPy. All other fits were performed using CMA-ES (73) via the PINTS (86) inference framework. When calculating benchmarks, methods 2–4 were run on a machine with 24 Intel Xeon 2.2 GHz CPU cores (48 with hyperthreading), with four optimizations running concurrently at all times. All data, code, results, and figures are available to download from https://github.com/CardiacModelling/FourWaysOfFitting. A permanently archived version is available on Zenodo at https://doi.org/10.5281/zenodo.3378030.

## Results

We now discuss the results of fitting a model with each of the four methods, using cell #5 as an example in our figures. Figures for all nine cells can be found online at https://github.com/CardiacModelling/FourWaysOfFitting. For each fit, we discuss the quality of fit but also investigate whether models made with the other methods do well at predicting the methods’ fitting data.

**Figure 4:**
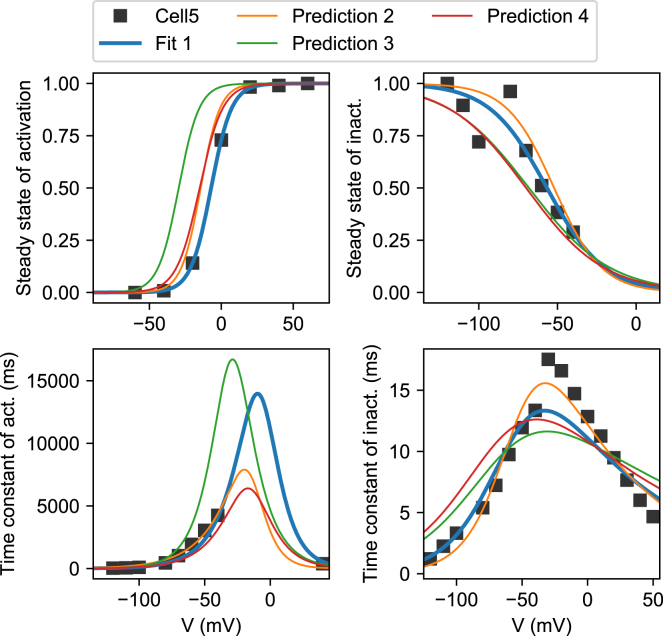
Method 1 goodness of fit and cross-validation on cell #5. Experimental approximations of the steady states (***ã***_∞_ and ${\tilde{r}}_{\infty }$) and time constants (${\tilde{\tau }}_{a}$ and ${\tilde{\tau }}_{r}$) are shown, derived from measurements in cell #5. The model curves for *a*_∞_(*V*), *r*_∞_(*V*), *τ*_*a*_(*V*), and *τ*(*V*) are shown for models fitted with each of the four methods. Note that only method 1 was trained on this data, making this a goodness-of-fit figure for method 1, whereas for the other methods, this figure shows a prediction. To see this figure in color, go online.

In method 1, we equate model variables (e.g., *a*_∞_) with experimentally derived values (e.g., *ã*_∞_). Fig. 4 shows the experimentally derived values as black squares, whereas the blue line (fit 1) represents the quality of fit obtained by method 1. Note that these lines are plotted directly from Eqs. 5 and 6 and do not involve simulation. Similar lines are shown for models fit with methods 2–4, labeled as “predictions” in the figure. As Fig. 4 shows, the lines drawn from the method 1 model fit the data well, whereas the models fitted through simulation (methods 2–4) show a notable mismatch for both steady states and time constants. However, because the summary curves approximate but do not equal the model variables, a perfect fit in this figure is not necessarily desirable. This is discussed further in Supporting Materials and Methods, Section S1.6.

**Figure 5:**
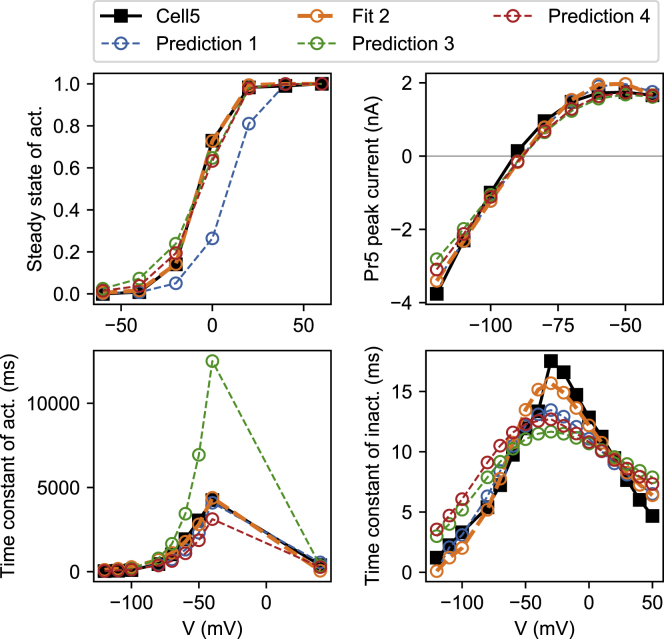
Method 2 goodness of fit and cross-validation on cell #5. The experimental steady state of activation, the IV curve, and both experimental time constants are shown. By simulating the protocols and performing the same analysis, similar summary curves were derived for each of the four methods. The fit for method 2 is shown, along with the predictions from models made with methods 1, 3, and 4. To see this figure in color, go online.

In Fig. 5, we again plot the experimentally derived data points (e.g., ${\tilde{a}}_{\infty }^{\text{cell}}$), but instead of comparing them to model variables, we compare them to simulation results (e.g., ${\tilde{a}}_{\infty }^{\text{sim}}$). Method 2 attempts to minimize the discrepancy between the two and achieves this well (fit 2 in Fig. 5). By contrast, the predictions from a model made with method 1 now show a clear shift in the steady state of activation curve. Interestingly, only the method-2-derived model shows a close fit to the experimental time-constant data, although none of the methods are able to match the steep peak in the ${\tilde{\tau }}_{r}$ points well, which may be due to the fact that points left of the peak were derived from Pr5, while the rightmost points were obtained from Pr4.

**Figure 6:**
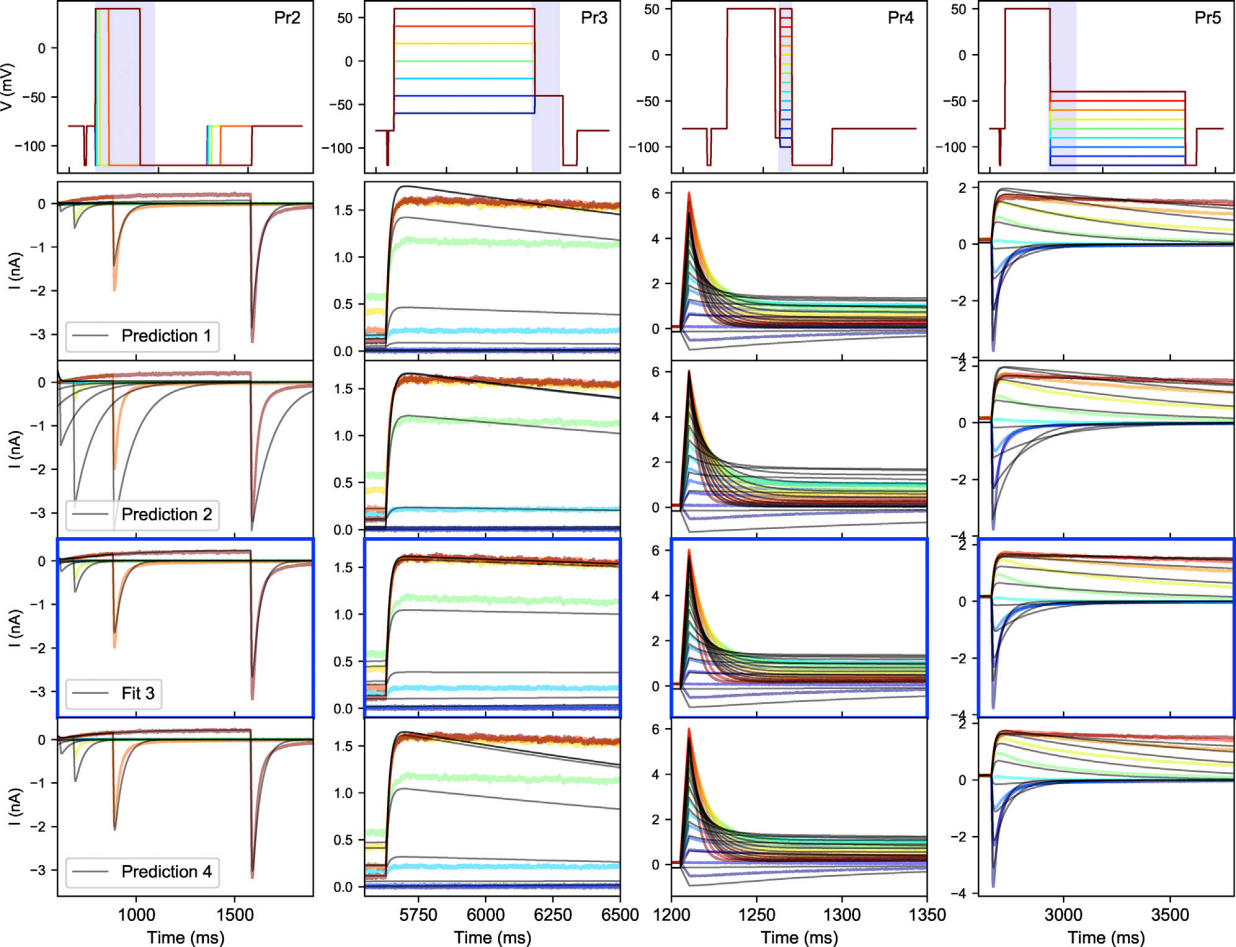
Method 3 goodness of fit and cross-validation on cell #5. The top row shows the voltage protocols Pr2–5, with different colors used for each sweep. The same colors are used in each of the panels below to show the corresponding experimental data, whereas simulation results are shown in black. In the top row, the full time span of each protocol is shown, but the plots shown below each zoom in on a specific region, indicated by the shaded area in the top row. The fit made with method 3 is highlighted with blue borders (fit 3) while the other rows show predictions from models made with methods 1, 2, and 4. To see this figure in color, go online.

Fig. 6 shows selected portions of the currents elicited by Pr2–5. Method 3 minimizes the discrepancy between simulated and measured currents and provides a good fit (fit 3 in the figure), although some differences can still be seen. Models made with methods 1 and 2 do not generally predict the observed currents well, although the qualitative behavior is correct in all cases. Models with method 4 provide better predictions here, although interestingly, only method 3 gets the slope of the Pr3 currents during the P2 step right, indicating a difference in the deactivation properties of method 3 parameters compared to the others. The negative currents in Pr4 appear challenging for all methods, whereas method 3 and 4 results match well on the higher potential steps.

**Figure 7:**
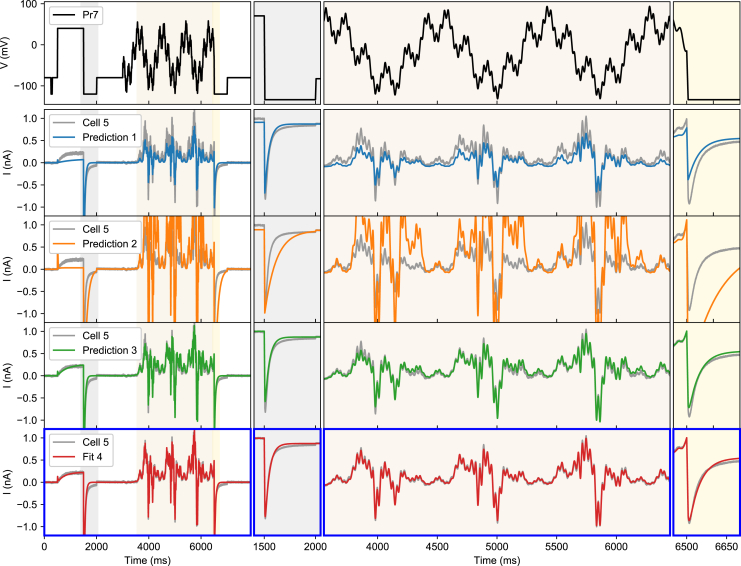
Method 4 goodness of fit and cross-validation on cell #5. The top panel shows the voltage protocol (Pr7) while the three panels below show the same current recording (*gray line*), and each row shows a prediction from a model made with methods 1–3, respectively (*colored lines*). The bottom panel, highlighted with blue borders, shows the fit for method 4 (fit 4). To see this figure in color, go online.

Next, we inspected the capability of the different models to predict the currents from the sine wave protocol (Pr7), as shown in Fig. 7. The method 4 model obtained a good fit to the data, although some differences can be seen in the deactivating part of the initial voltage step. Predictions from the model made with method 3 were relatively good, whereas models fitted with method 1 and in particular method 2 performed poorly at predicting Pr7 currents.

**Figure 8:**
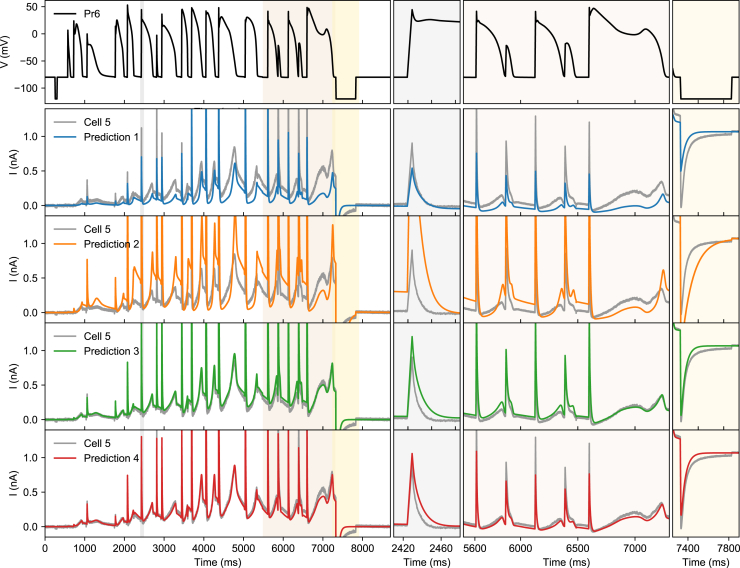
Validation on the AP waveform signal (Pr6). The voltage protocol is shown on the top row, with subsequent rows colored traces showing predictions from methods 1–4, respectively. The current measured in cell #5 in response to Pr6 is shown in gray (same in each row). None of the models were trained on Pr6 data, making this an independent validation. The left panels show the full duration of the signal, and the panels on the right zoom in on selected regions (2.41–2.48, 5.5–7.3, and 7.25–7.9 s). To see this figure in color, go online.

Figs. 4, 5, 6, and 7 showed that all four methods achieved good fits, judged by their own criteria, whereas the quality of predictions outside of the fitting data varied. The AP waveform is an attempt to test predictions for the most physiologically relevant *I*_Kr_ behavior. A visual comparison of predictions made with models from each of the four methods is shown in Fig. 8, again with the cell #5 results used as an example. In this cell, the predictions from method 2 were generally poor, whereas methods 3 and 4 provide much better predictions.

**Figure 9:**
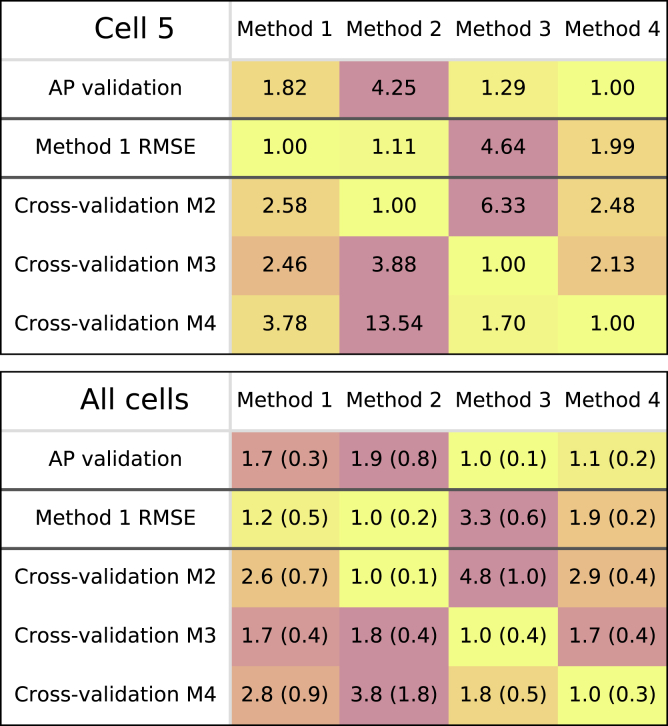
Validation and cross-validation results. (*Top*) Results for cell #5 are shown. Each row shows the relative RMSE for a fitting method, scaled so that the best performing method is indicated by 1, whereas a method with a relative score of, e.g., 1.2 had an RMSE that was 1.2 times larger. The top row shows the relative *E*_AP_ for each method, with the remaining rows showing *E*_M1_, *E*_M2_, *E*_M3_, and *E*_M4_, respectively. (*Bottom*) Mean results over all nine cells are shown, with SDs given in parentheses. To see this figure in color, go online.

A quantitative view of the validation and cross-validation results for cell #5 is given in Fig. 9 (*top*). The top row of this table shows the RMSE for the independent validation protocol. To enable easier comparisons, the RMSEs have been normalized to the best performing method so that the best method has relative RMSE 1, whereas a method with a relative value of 2 has an RMSE that is twice as high. For cell #5, method 4 provided the best predictions of the independent test protocol and also performed well on cross-validation. As may be expected, each method had the lowest score on its own fitting data. Data for all nine cells are given in Supporting Materials and Methods, Section S3.3.

The lower panel in Fig. 9 shows similar relative RMSEs but now presented as the mean and SD for all nine cells. Here, method 3 performed best at the AP prediction task, although method 4 was very close (within SD) and outperformed it at cross-validation.

Interestingly, method 2 outperformed method 1 on the *E*_M1_ criterion in the averaged data and also in six out of nine cells. This indicates the advantage of fitting steady states and time constants simultaneously (as happens in method 2) over fitting them sequentially (method 1), as previously described (33, 35). Further illustration is provided in a figure in Supporting Materials and Methods, Section S3.6, in which we show that small changes in the slope of the steady-state curve have a strong effect on the time constants derived by method 1; method 2 can use this to its advantage by slightly adjusting the model’s steady-state curve slope to obtain a better fit to experimental time-constant data.

### Reliability and performance

Having inspected the predictive capabilities and quality of fit of models obtained with each method, we next investigated their reliability. Ideally, a method returns the same result every time it is applied, and indeed, method 1 lives up to this ideal. For methods 2–4, however, we used 1) randomly sampled initial guesses for parameter sets and 2) a stochastic optimizer. To increase our chances of finding the best result, we repeated this process 50 times for methods 3 and 4 and 80 times for method 2. For a reliable method, we expect a large number of the methods to return similar parameter sets, with similar RMSEs.

**Figure 10:**
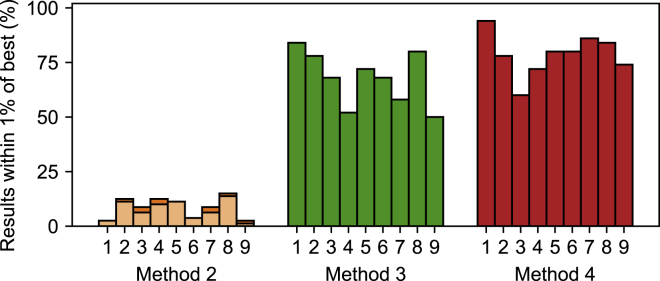
The percentage of optimizations from different starting points that found the “best result.” Darker bars in the background indicate the percentage of optimizations that returned a result with an RMSE within 1% of the best result found. The lighter bars in the foreground indicate the percentage that was also close in parameter space, (such that no single parameter varied by more than 1% from the best result found). Note that for methods 3 and 4, the bars overlap exactly, but the method 2 results for some cells contain “‘best” points with similar low RMSEs but different parameter values. To see this figure in color, go online.

Fig. 10 gives an indication of each method’s reliability. It shows that method 3 returned a result similar to the best one found on 50–84% of the repeats, depending on the cell. Method 4 appears slightly more reliable, with numbers ranging from 60 to 94%. For both these methods, results with a low RMSE value all had parameters clustered in a small area of the parameter space. For method 2, only a very small number of repeats returned a result with an RMSE similar to the lowest one found. In addition, the method 2 results with the lowest RMSE values were not always close together in parameter space. Method 1 is a deterministic method and so was omitted from this figure. This is further explored in Supporting Materials and Methods, Section S2.3.2.

**Figure 11:**
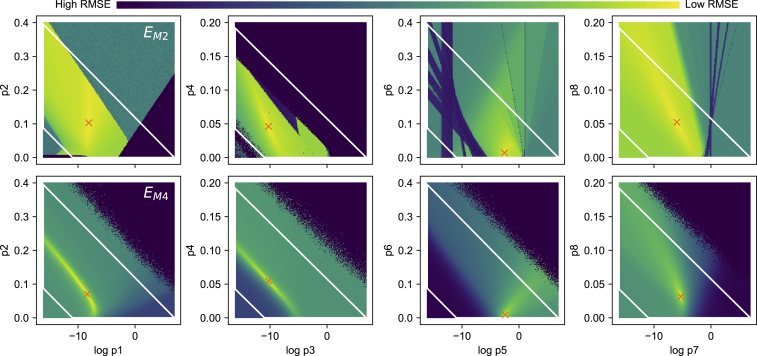
An exploration of two-dimensional slices of the objective functions. (*Top*) *E*_M2_ for cell #5 is shown, near the optimum found by method 2 (indicated by an *orange* “×”). Each panel shows the result of varying two parameters, with the other seven held constant. The parameters varied in the leftmost panels determine the rate of activation, followed by deactivation in the second panel, inactivation in the third, and finally, the rate of recovery. The white lines indicate the boundaries used during optimization. (*Lower*) An exploration of *E*_M4_ for cell #5 is shown, near the optimum found by method 4. The darkest purple color in the figure represents areas where either summary curve derivation (*top panel*) or simulation (*lower panel*) failed. To see this figure in color, go online.

To investigate the performance of the different methods, we measured the computation time for each method and counted the number of forward simulations that were performed during an optimization. On average, method 2 was much slower than method 3, which itself was slower than method 4 (shown in Supporting Materials and Methods, Section S3.4). The number of function evaluations was similar for methods 3 and 4, indicating that this difference in performance was due to the increased simulation time needed for method 3 (228 s of simulation for method 3 vs. 8 s of simulation for method 4). However, many repeats of method 2 were seen to terminate with a low number of evaluations, which may indicate these optimizations terminated early in a local optimum. To explore this hypothesis, we performed a brute-force exploration of *E*_M2_ and *E*_M4_ for cell #5 in the region near the optimum returned by methods 2 and 4, respectively, as shown in Fig. 11. For *E*_M4_, we see a clear optimum in each panel, and the function appears smooth (at least within the optimization boundaries, indicated by the *white lines*). For *E*_M2_, however, a lot of discontinuities can be seen. The darkest areas in these panels are regions where the intermediate analysis to derive time constants and steady states from the simulated experiments failed. As the rightmost panels show, this can occur in otherwise smooth parts of the slices, which may prove challenging for optimization routines. In addition, a lot of “noise” can be seen (e.g., in the *green areas* of the first panel), which may indicate the presence of many local minima—however, each panel is a two-dimensional slice of a nine-dimensional space, so this is not necessarily the case.

## Discussion

We defined four methods, each representative of a wider class, to fit ion current models using whole-cell current recordings. Methods 3 and 4, both based on whole-current fitting, were found to provide the most accurate predictions, whereas methods 1 and 2, both based on fitting preprocessed “summary” data, fared poorly. Of the methods in which we applied a stochastic optimization routine (2, 3, 4), methods 3 and 4 were found to provide the most consistent results, and method 4 was the most time-efficient both in terms of experimental and computational effort.

**Figure 12:**
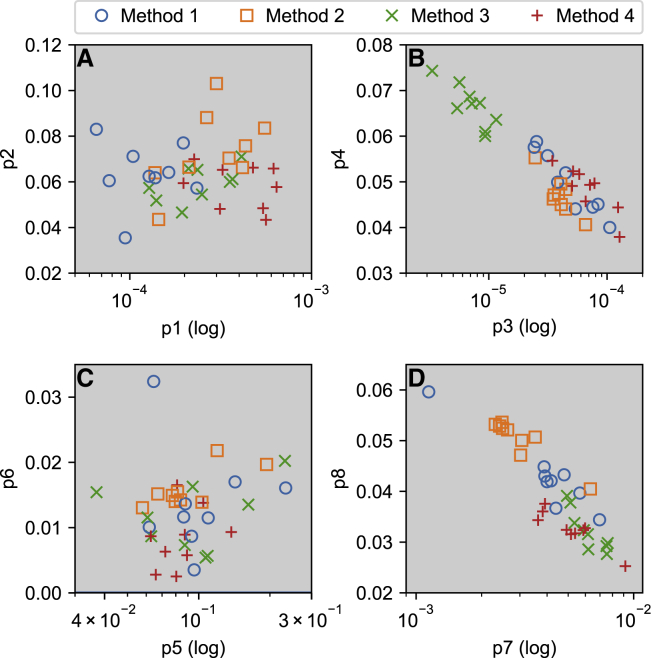
Best solutions returned by the four methods for all cells. The activation rate parameters *p*_1_ and *p*_2_ are shown in (*A*), deactivation in (*B*), and inactivation and recovery in (*C*) and (*D*), respectively. To see this figure in color, go online.

To further compare the results from different methods, we plotted the fits from each method and each cell in Fig. 12. Even in areas where parameter sets overlap, each method can be seen to introduce its own small bias. The figure also points to a difference in deactivation for method 3, which consistently placed the deactivation parameters *p*_3_ and *p*_4_ in a different part of the parameter space than the other methods. We found that models using parameter sets found by method 3 gave the best AP predictions, although this was very closely followed by method 4 (see Fig. 9). Looking at Fig. 6, we can see that many cases in which method 1, 2, and 4 predictions deviated from the measured current were during deactivation. This points to an advantage in describing deactivation for method 3 and suggests an area in which future versions of the sinusoidal protocol used in method 4 could be improved. In particular, the slow timescale of activation and deactivation may necessitate the inclusion of long steps similar to those in Pr3/Pr5 or the superposition of a lower frequency sine wave.

For methods 2–4, we observed significant improvements to the performance of optimization routines by performing a log transform on some of the parameters, providing appropriate constraints on rates and voltage-dependence of the rates, and refining differential equation numerical solver tolerances. At least some of these aspects may address previous findings that whole-trace fitting is difficult (58), as we found very consistent results with an “off-the-shelf” optimizer after adopting these simple approaches.

The optimization surface for method 2 is considerably more ragged than methods 3 and 4, making this a difficult optimization problem. As method 2 is very common in the literature on ionic model fitting, this result has some implications for the cardiac modeling community: its reputation as a hard problem requiring special methods may be due to the choice of summary data rather than any intrinsic mathematical or computational difficulty with the models. Since the choice of method 2 is typically based on data availability (48), increased sharing of whole-cell current data is a more promising way forward than developing and applying novel optimization algorithms. For experimenters, as well as universities, journals, and other publishers of scientific data, our findings re-emphasize the need for sharing of raw data, for example, in online repositories or data supplements (87).

In cases in which full currents are not available (i.e., when using historical data), some steps may be taken to alleviate the problems associated with method 2. Reviewing Fig. 11, some of the high error parts of the *E*_M2_ surface arise from numerical issues during the derivation of summary curves from simulated experiments (for instance, in deriving time constants from almost-flat current traces). It may be possible to standardize and improve these analysis methods and so remove some of the discontinuities in *E*_M2_.

Another approach we tried to improve methods 2 and 3 is to use method 1 to propose an initial guess parameter set for methods 2 and 3 instead of sampling a point from randomly within the parameter constraints, as hinted at previously (88). In initial tests, this gave very similar results to the “full” methods but with only a single optimization repeat needed. The full results are shown in Supporting Materials and Methods, Sections S3.7 and S3.8.

A reason sometimes given to continue using experimental steady-state and time-constant curves as a summary of current kinetics is that they allow comparison to published data and previous results from the same lab. So, method 3 may be seen to have an advantage over method 4 in that it contains the traditional protocols needed to derive these curves. However, as Vandenberg et al. (89) point out, it is important to realize that these values can be highly sensitive to the (occasionally unpublished) details of the protocols and analysis methods so that making these comparisons is not without danger. We have seen, though, that method 4 can provide excellent simulations of these protocols, so comparisons to previous data could be made by 1) running an experiment with a novel optimized protocol, 2) quickly and reliably fitting a model to the raw current data, and 3) simulating the previously used protocols and performing the analysis. Indeed, this method could be used to compare data sets (via modeling) from protocols with slight discrepancies (90).

### Limitations and further work

In this work, we characterized four methods of fitting an ion current model and proceeded to analyze and critique these methods based on our characterization. Although we tried not to misrepresent any method, it is therefore important to highlight any areas in which our efforts may become subjective or otherwise fall short.

Firstly, running both a full set of conventional protocols, as well as Pr6 and Pr7, in a single cell is experimentally infeasible. For that reason, the study by Beattie et al. (20) used truncated version of the conventional protocols (e.g., with fewer voltage sweeps) and omitted Pr2 variants with different P1 voltages. We must therefore admit the possibility that methods 1 or 2, performed with a larger data set, would lead to improved results. In particular, the experimental time constants of activation are “missing” in the range (−30 mV, 30 mV), which is exactly where the peak value should occur. However, given the excellent performance of method 3 in our study, it seems the required information was present in the recordings, so the limited efficiency of both methods still stands.

Another limitation stemming from the cell-specific nature of our measurements is that we cannot benefit from averaging as a noise-reduction strategy. As Fig. 2 shows, the mean summary curves over all nine cells often show qualitative behavior that is more like the expected results than our individual cell measurements. However, it has also been shown that averaging in cellular electrophysiology can lead to erroneous results (91), and there is a clear benefit of having methods that work on a single cell, thereby allowing investigations of cell-to-cell variability, heterogeneity, and measurements of cells only available in limited quantities (e.g., human tissue).

In our method 1, we did not use time-to-peak information (as suggested by (31)) and used what Willms et al. (35) refer to as the “disjoint method” rather than estimating time constants and steady states simultaneously. We did not, unfortunately, find much mention of such improved methodologies in the applied literature and so believe that our method 1 is still a good representation of a commonly followed approach. Similarly, the derivation of the summary curves might be improved in other ways, which would lead to better results for methods 1 and 2. As a counterpoint, we note that methods 3 and 4 do not require these complex intermediate analyses, are easier to implement, and do not rely on the assumption that the summary curves provide an accurate representation of the raw current data.

In deriving methods 2 and 3, subjective choices had to be made regarding weightings of the individual protocols, and changing these may alter the results. For example, similar error measures could be derived that do not normalize according to the length of each protocol or that use fixed weightings per protocol instead of the cell-specific maximal current ones we used. As it is based on a single protocol, method 4 does not require a weighting per protocol, which could be seen as an advantage because it means there are fewer subjective choices to be made during optimization. However, the lack of scalings could also be viewed as a weakness because it may be desirable (for method 4 but also 2 and 3) to scale different parts of the protocol(s) differently. Such an approach could, for example, compensate for varying current amplitudes (which otherwise cause the error function to emphasize fitting regions of the protocol that elicit stronger currents) or even weight “important” (in some sense) parts of the currents more strongly than others. For our study, we thought it advisable to avoid such “tweaking,” hopefully leading to a result that is representative of standard approaches and easily applicable.

Relatedly, error measures other than RMSE could have been used for the optimization objective. Using a statistical likelihood instead of an RMSE has an advantage in that it opens the door to Bayesian analysis, which would allow prior information to be used in fitting and could be used to shed more light on the origins of observed cell-to-cell differences (92). However, the assumption of independent and identically distributed Gaussian noise may not be the most appropriate one if there are significant drifts in the system or if the high sampling rate used in recording captures single bursts of noise in several adjoining time points. Different “noise models,” e.g., autoregressive or autoregressive moving average, might be used, but whether these are more appropriate models of voltage-clamp error is an open question that warrants further research. Because such error measures are not commonly used in the literature, our comparison here was restricted to RMSE.

Various models of hERG and *I*_Kr_ currents have been proposed and compared in the literature (20, 93), and so far, a consensus has not been reached. All four methods in this study used the same two-gate Hodgkin-Huxley model and so share the common limitation that this model may not be the optimal choice. Studies such as Bett et al. (93) have shown that some experiments are better fitted with a five-state Markov model (94), which suggests that the method 3 and 4 fits shown in Figs. 6 and 7 could be further improved. The study by Beattie et al. (20) investigated this question (section H in its Appendix) and confirmed that the model by Wang et al. (94) provided better fits to the sine wave data. However, predictions of the AP waveform (Pr6) response (as well as the metrics derived from Pr2–5) were no more accurate than those made with the simpler four-state model. This finding suggests there is still a tradeoff to make between fitting training data precisely and predictive power in new situations, and the choice of an optimal model depends on the expected “context of use” (95). However, we expect that further refinements to either models or fitting protocols could produce better fits and predictions, an area in which further work is needed.

Conversely, one might question whether the model used in this study is already more complex than the data warrant, commonly termed “overfitting”: when a model is fitted to (some of the) noise in the training data, leading to an improved fit but a loss of predictive power in new situations. Using methods 3 and 4, however, we found we could accurately predict the response to the independent validation data of Pr6 (Fig. 8) and that models trained on Pr7 could predict the response to Pr2–5 (Fig. 6) and vice versa (Fig. 7), so we do not believe overfitting is an issue for this simple model.

Finally, it is worth discussing how our results may be generalized beyond Hodgkin-Huxley-style independent gating models. As noted in the introduction, method 1 requires a model for which analytic expressions for steady states and time constants are available, which means it does not generalize to arbitrary Markov models. For Markov models of intermediate complexity—but with dependent gating—we expect our results for methods 2–4 will generalize well, although more complex models with many states and transition parameters to capture may require more complex “method-4-style” protocols to be devised. We believe this is an exciting area for future work.

## Conclusions

We presented and compared four methods to fit an ion current model, each based on a common class of methods used in the literature. By performing these methods on a set of CHO-cell hERG1a measurements, we found that methods based on whole-current fitting provided both the most accurate and the most reproducible results. Models fitted using a recently developed sinusoidal protocol were found to have similar predictive ability as those fitted to conventional voltage-step protocols while having a lower experimental and computational cost. Further analysis showed that using numerical optimization to fit a model to experimental steady-state and time-constant data was potentially hazardous, and we point toward increased sharing and use of raw current traces as a viable solution to this problem. Our results highlight some of the remaining challenges to make truly predictive models of ionic currents and the possibilities for novel experimental protocols to enable faster and more reliable fitting.

## Author Contributions

M.C., D.J.G., and G.R.M. designed and performed the analysis. K.A.B. performed the experiments. K.A.B. and G.R.M. designed the experimental protocols. M.C., D.J.G., and G.R.M. wrote the manuscript. All authors approved the final version of the manuscript.

## Supporting Material

Document S1. Supporting Materials and Methods, Figs. S1–S27, and Tables S1–S5

Document S2. Article plus Supporting Material
