# Supplementary material for: Four Ways to Fit an Ion Channel Model
Source: Biophys J. 2019 Aug 6;117(12):2420–37. doi: 10.1016/j.bpj.2019.08.001 (PMC6990153; doi:10.1016/j.bpj.2019.08.001)
Supplement: Document S2. Article plus Supporting Material [file mmc2.pdf]

Biophysical Journal
Article

Biophysical Society

Four Ways to Fit an Ion Channel Model

Michael Clerx,[1] Kylie A. Beattie,[1] David J. Gavaghan,[1] and Gary R. Mirams[2,*]
[1]Computational Biology & Health Informatics, Department of Computer Science, University of Oxford, Oxford, United Kingdom and [2]Centre for Mathematical Medicine & Biology, School of Mathematical Sciences, University of Nottingham, Nottingham, United Kingdom

ABSTRACT Mathematical models of ionic currents are used to study the electrophysiology of the heart, brain, gut, and several other organs. Increasingly, these models are being used predictively in the clinic, for example, to predict the risks and results of genetic mutations, pharmacological treatments, or surgical procedures. These safety-critical applications depend on accurate characterization of the underlying ionic currents. Four different methods can be found in the literature to fit voltage-sensitive ion channel models to whole-cell current measurements: method 1, fitting model equations directly to time-constant, steady-state, and I-V summary curves; method 2, fitting by comparing simulated versions of these summary curves to their experimental counterparts; method 3, fitting to the current traces themselves from a range of protocols; and method 4, fitting to a single current trace from a short and rapidly fluctuating voltage-clamp protocol. We compare these methods using a set of experiments in which hERG1a current was measured in nine Chinese hamster ovary cells. In each cell, the same sequence of fitting protocols was applied, as well as an independent validation protocol. We show that methods 3 and 4 provide the best predictions on the independent validation set and that short, rapidly fluctuating protocols like that used in method 4 can replace much longer conventional protocols without loss of predictive ability. Although data for method 2 are most readily available from the literature, we find it performs poorly compared to methods 3 and 4 both in accuracy of predictions and computational efficiency. Our results demonstrate how novel experimental and computational approaches can improve the quality of model predictions in safety-critical applications.

SIGNIFICANCE Mathematical models have been constructed to capture and share our understanding of the kinetics of ion channel currents for almost 70 years, and hundreds of models have been developed using a variety of techniques. We compare how well four of the main methods fit data, how reliable and efficient the process of fitting is, and how predictive the resulting models are for physiological situations. The most widely used traditional approaches based on current-voltage and time constant-voltage curves do not produce the most predictive models. Short, optimized experimental voltage-clamp protocols are as predictive as ones derived from traditional protocols, opening up possibilities for measuring ion channel kinetics faster, more accurately, and in single cells.

INTRODUCTION

Computational models of ionic currents have been used to understand the formation of the cellular action potential (AP) (1–3), to simulate the effects of genetic mutations (4), and to study and predict the effects of pharmaceutical compounds that block various ion channels (5,6). By fitting mathematical models to experimentally measured currents, we can learn about the kinetics of the underlying ion channels in healthy and in pathological situations. The need to understand voltage-sensitive ion channels' function is widespread as they are key players in neuronal excitation (1), cardiac contraction (7), gastric function (8), insulin secretion (9), and several other aspects of physiology (10,11). In cardiac arrhythmia research, models of ionic currents are routinely integrated into models of the AP and used to investigate the effects of genetic mutations, predict proarrhythmic risk in drug development, and inform clinical interventions (12–14). Such safety-critical applications depend on accurate characterization of the underlying ionic currents.

Several methods have been proposed to fit (parameterize) models of ionic currents, covering a range including pen-and-paper methods of the 1950s, detailed mathematical analysis specific to Hodgkin-Huxley models, and numerical "black-box" optimization. Data from several sources have been used, including whole-cell (aggregate) currents (1), single-channel currents (15), AP recordings (16),

Submitted April 15, 2019, and accepted for publication August 1, 2019.
*Correspondence: gary.mirams@nottingham.ac.uk
Editor: Kenneth Campbell.
https://doi.org/10.1016/j.bpj.2019.08.001
© 2019 Biophysical Society.
This is an open access article under the CC BY license (http://creativecommons.org/licenses/by/4.0/).

dynamic-clamp experiments (17), gating currents caused by the movement of charged parts of the ion channel proteins themselves (18), and measurements of fluorophores whose visibility varies with channel conformation (19).

In this manuscript, we focus on the common problem of fitting a set of predetermined model equations to voltage-dependent whole-cell ionic currents recorded under voltage clamp.

We describe four methods of fitting, each representative of a wider class of methods used in the electrophysiology literature. Our case study uses data from a previous study, in which currents were recorded at room temperature in nine Chinese hamster ovary (CHO) cells stably expressing hERG1a (20). Each cell was subjected to a series of training and validation protocols. All protocols were run on every cell so that we can apply all four methods to each cell, creating four model parameterizations, and then evaluate their ability to predict the current measured in the validation protocol. Finally, we discuss some of the reasons behind the observed differences in performance and compare the methods' repeatability and time efficiency.

The first publication describing and fitting a model of ionic currents was by Hodgkin and Huxley (1). Using the idea that ionic conductance depends on some parts of the membrane being in one of two states, they postulated simple "gating" processes, each with a forward and a backward transition rate from which a time constant and a steady state could be deduced. They then set about designing protocols to measure (or approximate) these time constants and steady states at several voltages, fitted curves through these measurements, and used the resulting (phenomenological) equations to encode their transition rates. Similar methods of fitting the equations directly to experimentally derived points—using mathematical analysis, least-squares fitting, or manual adjustment—were employed by subsequent modelers (21–30).

In this work, we describe a version of this method and refer to it as "method 1." It is important to note that these methods rely on particular assumptions: 1) that the underlying biophysics is accurately described by one or more independent gating processes, each with their own steady-state and time-constant variables; and 2) that an experimental method and analysis procedure exists that will yield accurate values for these variables throughout a range of physiologically relevant voltages. The first assumption underlies all Hodgkin-Huxley-style models and any Markov models that have an equivalent Hodgkin-Huxley formulation. (Hodgkin-Huxley models are a subset of a more general class of Markov models. See, for example, Beattie et al. (20), which presents the equivalent Markov formulation of the Hodgkin-Huxley model used in this study, or Rudy and Silva (3), which provides examples of Markov models with and without Hodgkin-Huxley counterparts.) Testing whether the second assumption is violated can be done using simulated experiments, but proving it holds is challenging. A detailed mathematical analysis of two protocols was performed by Beaumont et al. (31), who showed that steady states can be obtained from peak currents only if there is a large difference in the time constants of the system, and that once these are known, the time constants can be estimated using time-to-peak measurements or by fitting exponential curves. In a follow-up study, they presented an iterative procedure to estimate the steady states for systems with more similar ("nonseparable") time constants (32). In Wang and Beaumont (33), this analysis was taken further, and a method was derived to estimate steady-state equations and time constants simultaneously and further improve the results (the idea to fit both parameters together had also been used previously by Ebihara and Johnson (34)). Willms et al. (35) provided another mathematical analysis of fitting two-gate Hodgkin-Huxley models and concluded that separate estimation of time constants and steady states ("the disjoint method") can lead to poor results. A further critique of method 1 was given by Lee et al. (36), who used mathematical analysis and simulation to point out errors arising for nonseparable time constants, and introduced methods aimed to combat this effect.

Despite these critiques, method 1 remains highly popular due of its simplicity. Estimates for time constants and steady states at different voltages are easily obtainable from the literature, which is a distinct advantage when multiple currents must be considered, e.g., when creating full AP models (7,37–39).

An alternative to method 1, but using the same data points, is to simulate the voltage protocols and the subsequent analysis to obtain a set of simulated steady states and time constants to match the experimental ones. The parameters used in the simulation can then be updated in an iterative fashion (manually or using numerical optimization) until the simulated values match the experimental ones. This procedure allows both Hodgkin-Huxley and more generic Markov models to be fitted to published time-constant and steady-state data (although a unique fit is not guaranteed). If the modeler uses the same analysis method that was applied to the experimental data, any imperfections in the approximations of the steady states and time constants are replicated in the simulation so that good results may be obtainable even when method 1 approximations are poor (e.g., if time constants are nonseparable). In this manuscript, we use the name "method 2" to describe a version of this method based on numerical optimization.

Like method 1, method 2 is prevalent in the AP-modeling literature (40–47). Further users include Moreno et al. (48), who mention the prevalence of published summary curve data as a reason to use method 2 rather than direct fits to current traces, as well as Perissinotti et al. (49), who provide a software package for performing method 2 optimizations. A tool by Teed and Silva (50) similarly implements a method 2 but also varies model structure when searching. Rather than manually tuning model parameters, several optimization

algorithms have been applied to method 2, including simulated annealing (50), a Davidon-Fletcher-Powell optimizer (51), subspace trust region methods (45), and the Nelder-Mead simplex method (45,48).

Instead of fitting to processed "summary data" (time constants and steady states), we can also fit simulated currents directly to measured currents from the same protocols: in this manuscript we term this "method 3." Like method 2, method 3 is applicable to Markov-style models. Because method 3 does not require calculation of time constants and steady states, it is insensitive to errors in this process and can be more computationally efficient. A downside is that full current traces are more difficult to obtain and require the experimenter to have published their findings in digital format rather than printed tables or summary graphs.

The applicability of method 3 to Markov models was exploited by Balser et al. (52), who used numerical optimization and whole-current fitting to find the parameters of a model describing a cardiac potassium current. Similarly, Irvine et al. (53) formulated a Markov model of the cardiac fast sodium current and fitted it using method 3 (although they used additional data sources beyond whole-cell currents). Several algorithms have been used for the optimization step in method 3, including the Levenberg-Marquardt algorithm (52,54), Nelder-Mead (52), simulated annealing (53), differential evolution (55), and genetic algorithms (56,57), as well as hybrid methods combining multiple algorithms (58). Willms et al. (35) used simulated data to show advantages of method 3 over method 1 and provided a software tool for method 3 fitting (59).

Several authors have also compared fitting methodologies using identifiability analysis. A working definition of practical parameter identifiability is that fitting a particular data set constrains the parameters of a model to a small region of parameter space. As such, identifiability can be used as a tool to compare model complexity, fitting algorithms, and protocol designs. An extensive analysis of parameter estimation and identifiability for Markov models of ligand-gated channels was given by Milescu et al. (60), while Fink and Noble (61) used a sensitivity-based method to detect identifiability issues in published models and optimize voltage protocols. Csercsik et al. (62) investigated a procedure falling somewhere between methods 1 and 3, in which the parameters underlying voltage-dependence of steady states were determined, but time constants were fitted separately for different voltages. Walch and Eisenberg (63) considered the problem of estimating time constants and steady states as scalars for several voltages independently and concluded that in this case, only the time constants were identifiable. As Csercsik et al. (62) point out, full identifiability can be obtained by using voltage-dependent functions for the steady-state and time-constant equations.

In method 3, we do not calculate time constants or steady states, but we still use protocols designed specifically to estimate them. A next natural step, then, is to reconsider the established protocols and to ask what type of protocol would work best for method 3. We define "method 4" as the process of fitting directly to currents from a single protocol designed to characterize multiple aspects of current kinetics at once.

Perhaps the first demonstration of method 4 was by Millonas and Hanck (64), who fit a Markov model of a cardiac sodium current using a protocol consisting of random fluctuations between two voltages ("dichotomous noise"). This method was later applied to design protocols specifically to differentiate between competing models (65–67). A brief mention also occurs in Gurkiewicz and Korngreen (56), which discusses the benefits of method 3 for semiautomatic "high-throughput" construction of ionic current models and notes that it could be applied to "nonstandard protocols." Fink and Noble (61) used identifiability analysis and simulation to show that standard voltage protocols can be considerably shortened while still being sufficient to parameterize various models. Importantly, they showed that protocols can be created that provide information on parameters for multiple cardiac currents so that it may be possible to formulate protocols without extensive preknowledge of the system (as is typical for standard protocols) and that protocols might be designed that are robust to changes induced by, e.g., drugs or mutations. Subsequent work by Clerx et al. (68) showed that the identifiability analysis employed by Fink and Noble (61) could be extended to take into account experimental errors. Finally, a study by Beattie et al. (20) proposed and tested a protocol design based on sinusoidal voltage clamps. Their work showed that a model could reliably be fit to the resulting current measurements and that models created this way outperformed published models in predicting the response to conventional voltage-clamp protocols and an AP waveform protocol.

Having discussed the four methods, we should point out that many studies do not stick to using a single method but use different approaches for different model parameters (69). In the remainder of this work, we formulate the four methods that characterize the approaches discussed above and evaluate their performance on a previously published experimental data set.

Terminology and notation

Concepts such as "the time constant of activation" can be interpreted either as a property of a (macroscopic) current through the cell membrane or as a property of (microscopic) transitions of individual channels. Similarly, they can be seen either as the results of a particular set of (whole-cell) experiments or as model variables that may or may not have a physical counterpart. In this manuscript, we will use terms such as "model time constant" and "experimental time constant" to distinguish between model variables and experimentally derived values where necessary. When using

mathematical symbols, we will use a tilde notation to denote experimentally derived values (e.g., $\tilde{\tau}_a$ for the experimental time constant of activation) and a plain notation for model variables (e.g., τ_a for the model time constant of activation). When referring to a vector of values at different voltages, we use boldface symbols (e.g., $\boldsymbol{\tau}_a$ and $\tilde{\boldsymbol{\tau}}_a$). Finally, when referring to the full set of experimentally derived measures, we have tried to consistently use the term "summary curves" (based on the notion of a collection of "summary statistics" or "biomarkers").

MATERIALS AND METHODS

Current model

To model the hERG current, we use a two-gate Hodgkin-Huxley model found to provide excellent predictions by Beattie et al. (20) (described there in equivalent Markov model form). The current is defined by

$$I_{\mathrm{Kr}} = g_{\mathrm{Kr}} \; a \; r(V - E_K), \tag{1}$$

where g_{Kr} is the maximal conductance, a and r are gating variables (defined below), V is the trans-membrane potential, and E_K is the reversal potential for potassium ions (note that although the measurements we used were from CHO cells expressing hERG1a, we use the shorthand terms I_{Kr} and g_{Kr} throughout this work). The Nernst equation was used to calculate a separate E_K value for each cell:

$$E_K = \frac{RT}{F} \ln \frac{[\mathrm{K}^+]_{\mathrm{o}}}{[\mathrm{K}^+]_{\mathrm{i}}}, \tag{2}$$

where R is the gas constant, F is the Faraday constant, $[\mathrm{K}^+]_{\mathrm{o}}$ and $[\mathrm{K}^+]_{\mathrm{i}}$ are the bath (outside membrane) and pipette (inside membrane) concentrations of potassium ions, and T is the cell-specific temperature measured by Beattie et al. (20). In the nine cells used in this study, calculated values for E_K ranged from −88.45 to −88.30 mV with the precise value depending on the temperature at the time of recording.

The two processes represented independently by this Hodgkin-Huxley model are "activation" (with a representing the fraction of activated channels) and "inactivation" (with r representing the fraction of channels that have recovered from inactivation). Both variables a and r are dimensionless and in the range [0, 1]. Increases in a correspond to "activation" and decreases in a to "deactivation," whereas decreases in r correspond to "inactivation" and increases in r to "recovery" from inactivation. (Note that the independence assumption means that—somewhat confusingly but perhaps appropriately—channels can be both "activated" and "inactivated" at the same time, because the opposite of activation is called deactivation and the opposite of inactivation is recovery.) We can represent the model as two independent gating reactions

$$(1-a) \underset{k_2}{\overset{k_1}{\rightleftharpoons}} a \qquad\qquad r \underset{k_4}{\overset{k_3}{\rightleftharpoons}} (1-r), \tag{3}$$

where a is the fraction of channels in the activated state, $(1-a)$ is the fraction in the deactivated state, r is the recovered fraction, $(1-r)$ the inactivated fraction, and k_1 to k_4 are the voltage-dependent transition rates. The ordinary differential equations governing a and r are then derived with mass-action kinetics and can be written in the form

$$\frac{\mathrm{d}a}{\mathrm{d}t} = \frac{a_\infty - a}{\tau_a}, \qquad \frac{\mathrm{d}r}{\mathrm{d}t} = \frac{r_\infty - r}{\tau_r}, \tag{4}$$

where a_∞ and r_∞ denote the model's voltage-dependent steady states and τ_a and τ_r denote its voltage-dependent time constants, defined in terms of the transition rates as

$$\tau_a = 1/(k_1 + k_2), \qquad \tau_r = 1/(k_3 + k_4), \tag{5}$$

$$a_\infty = k_1\tau_a, \qquad r_\infty = k_4\tau_r. \tag{6}$$

The voltage dependencies of the transition rates are defined using an Eyring-derived exponential formulation (53,70–72) as

$$k_1 = p_1\exp(p_2V), \qquad k_3 = p_5\exp(p_6V), \tag{7}$$

$$k_2 = p_3\exp(-p_4V), \qquad k_4 = p_7\exp(-p_8V). \tag{8}$$

The model parameters to be inferred are therefore the kinetic parameters $p_1, p_2, \ldots, p_8$ and the conductance $p_9 = g_{\mathrm{Kr}}$. All model parameters are taken to be strictly positive: $p_i > 0$ for $i \in 1, 2, \ldots, 9$.

Experimental methods

The experimental data used in this study are taken from Beattie et al. (20). In short, manual patch-clamp recordings were performed at room temperature (between 21 and 22°C) in CHO cells stably expressing hERG1a (which encodes the α subunit of the channel carrying I_{Kr}). Recordings were taken from nine cells and seven protocols in each cell (we will refer to these as cells #1 to #9 and Pr1 to Pr7, with the numbering matching the original publication). After the final protocol was completed, the I_{Kr} blocker dofetilide was washed in, and all protocols were repeated. Each cell's data was then postprocessed by first leak-correcting the signals recorded both in the presence and absence of dofetilide and then subtracting the I_{Kr}-blocked signal from the unblocked one to remove any endogenous currents. For this study, we used the already leak-corrected and dofetilide-subtracted data as published on https://github.com/mirams/sine-wave. The first protocol, Pr1, did not elicit strong currents in any of the cells and so was not used in this study.

Following Beattie et al. (20), capacitance artifacts were removed from the experimental data by discarding the first 5 ms after each discontinuous voltage step. To obtain similar results from simulated protocols, the same filtering was applied to all simulated data.

The six protocols used in this study are shown in Fig. 1. The first four, Pr2–5, are adaptations of common step protocols used to characterize I_{Kr}. Specifically, Pr2 is used to estimate a single time constant of activation (for $V = +40$ mV), Pr3 is used to estimate the steady state of activation, Pr4 is used to estimate time constants of inactivation, and Pr5 provides data about both time constants, as well as the steady state of inactivation. Pr7 is a novel sinusoidal protocol introduced by Beattie et al. (20) and is intended to provide the same information in a much shorter time. It consists of an initial step to +40 mV, designed to trigger a large current, followed by a section consisting of the sum of three sine waves. Finally, Pr6 is a collection of AP wave forms, representing the membrane potential under physiological and pathological conditions. As in Beattie et al. (20), we used Pr6 as a validation protocol while either Pr7 or the set Pr2–5 were used for fitting. Note that the full set of protocols was run on every cell.

A detailed description of all protocols and the associated analysis methods is given in Supporting Materials and Methods S1, Section S1. In analyzing these protocols, we found it useful to perform simulations (using the parameters obtained by Beattie et al. (20)) and plot the model state in a two-dimensional phase diagram as shown in Fig. 1. A guide to using these diagrams for analysis is provided in Supporting Materials and Methods, Section S1.1.

The voltage-step protocols Pr2–5 can be used to derive a set of graphs that characterize the current, shown in Fig. 2 for all nine cells used in

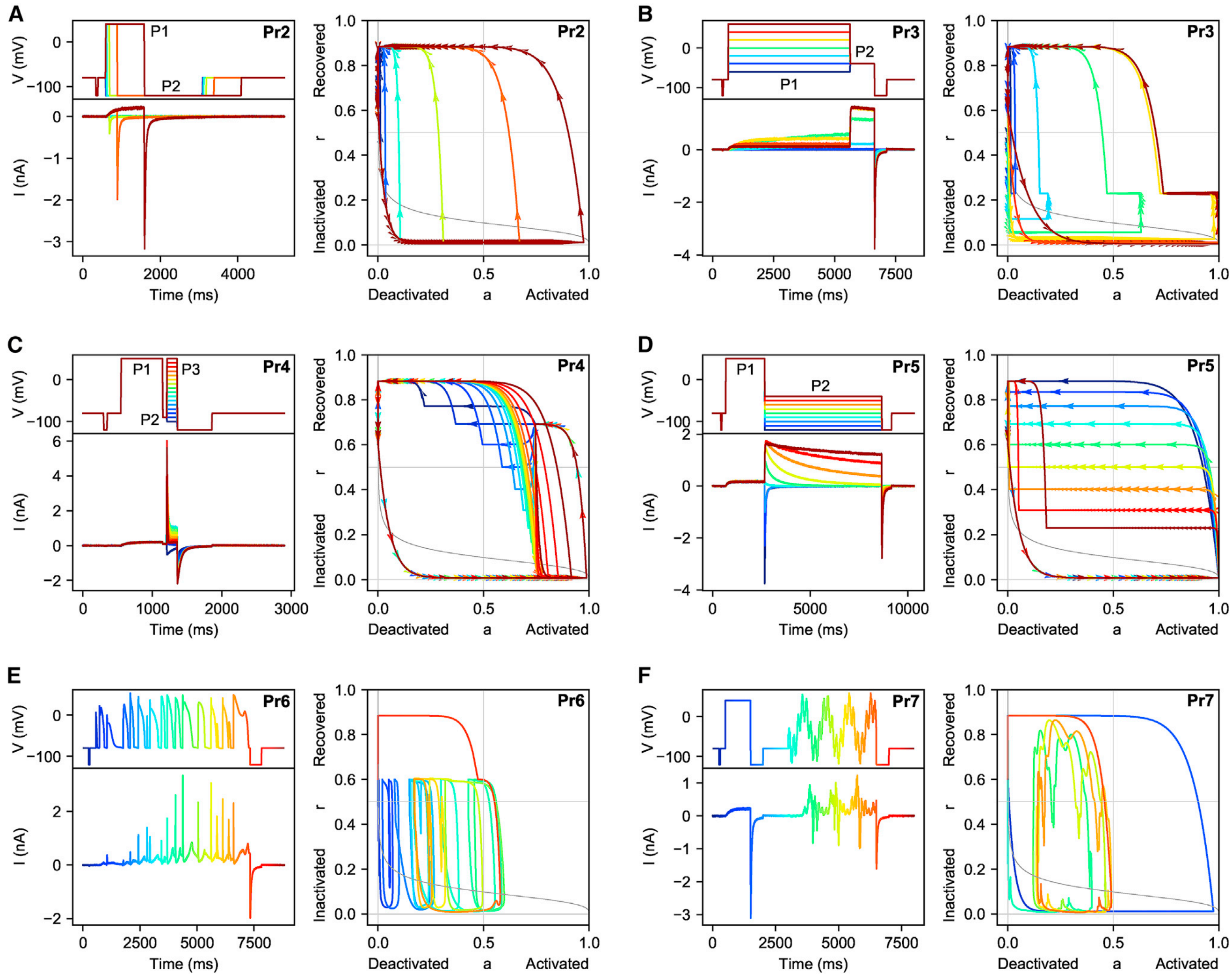

FIGURE 1 Voltage protocols, currents measured in cell #5, and simulated phase diagrams for all six protocols (Pr2–7) used in this study. Simulations for the phase diagrams were performed with the cell #5 parameters obtained in Beattie et al. (20). (*A*) Pr2 is used to measure the experimental time constant of activation. We show the voltage-step protocol (*top left*), the resulting current as measured in cell #5 (*lower left*), and a phase plane diagram (*right*). The protocol is repeated six times, with an increasing duration of the P1 step for each repeat. This is indicated in the plots by using a different color for each repeat. (*B*) Pr3 is used to measure the experimental steady state activation curve. It is repeated seven times, with a different voltage for the P1 step at each repeat. (*C*) Pr4 is used to measure experimental time constants of inactivation; it repeats 16 times with a different voltage for step P3. (*D*) Pr5 is used to measure experimental time constants of activation and inactivation, as well as steady-state inactivation. It has nine repeats with a different voltage for step P2. (*E*) Pr6 consists of several (healthy and pathological) AP wave forms and was used as an independent validation data set in this study. The colors in this plot indicate time so that the voltage and current traces can be related to the phase-plane trajectory on the right. (*F*) Pr7 is the sine wave protocol introduced by Beattie et al. (20). As in (*E*), color is used to indicate time information. To see this figure in color, go online.

this study. They are commonly referred to as the "steady state of activation" (Fig. 2 *A*), "steady state of inactivation" (Fig. 2 *B*), "[Peak/tail] IV curve" (Fig. 2 *C*), "time constant of activation" (Fig. 2 *D*), and "time constant of inactivation" (Fig. 2 *E*). In the remainder of the work, we will refer to the curves in Fig. 2 as the summary curves.

Four ways of fitting

Method 1: Fitting model equations directly to summary curves

We now describe the four methods of fitting in detail. In method 1, we write out equations for the model variables $a_\infty(V)$, $r_\infty(V)$, $\tau_a(V)$, and $\tau_r(V)$ in terms of the parameters $p_1, p_2, \ldots, p_8$ and then try to fit these directly to the experimental summary curves $\tilde{\boldsymbol{a}}_\infty$, $\tilde{\boldsymbol{r}}_\infty$, $\tilde{\boldsymbol{\tau}}_a$, and $\tilde{\boldsymbol{\tau}}_r$. The procedure for doing so closely follows that of Hodgkin and Huxley, and in fact, the procedure for finding p_1 to p_8 can be done entirely with pen and paper, and only a single simulation is needed to estimate the ninth parameter g_{Kr}. First, we focus on the steady state of activation (Eq. 6) and substitute in the parameters p_1 to p_4:

$$a_\infty(V) = k_1\tau_a = \frac{k_1}{k_1 + k_2} = \frac{1}{1 + k_2/k_1}, \tag{9}$$

$$= \left[1 + \frac{p_3 e^{-p_4 V}}{p_1 e^{p_2 V}}\right]^{-1}, \tag{10}$$

$$= \left[1 + e^{(\log p_3 - \log p_1) - (p_2 + p_4)V}\right]^{-1}. \tag{11}$$

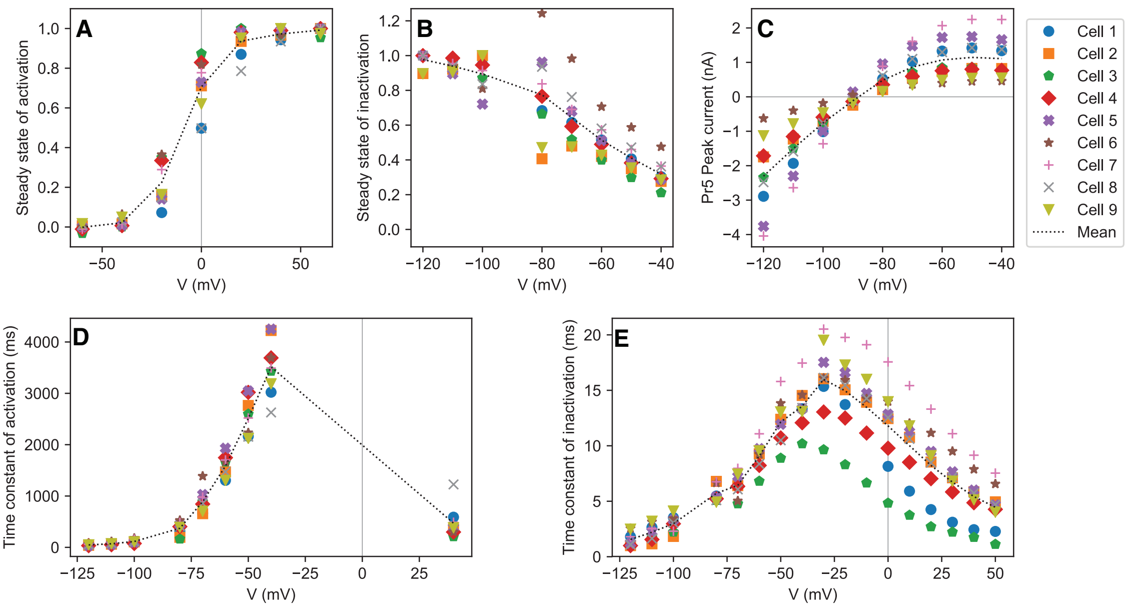

FIGURE 2 Summary curves calculated from the voltage-step protocols Pr2–5 for all nine cells. The mean for all cells is indicated with a dotted line. Note how the general trend, not always obvious from the single-cell results, is clearly captured by the line connecting the means. (*A*) The experimental activation curve $\tilde{\boldsymbol{a}}_\infty$ derived from Pr3 is shown. (*B*) The experimental steady state of inactivation $\tilde{\boldsymbol{r}}_\infty$ derived from Pr5. (*C*) The IV curve (peak current during the P2 step of Pr5) plotted against the P2 voltage. (*D*) The experimental time constant of activation $\tilde{\boldsymbol{\tau}}_a$. Values for $V < 0$ mV were estimated from Pr5, the single value at 40 mV from Pr2. (*E*) The experimental time constant of inactivation $\tilde{\boldsymbol{\tau}}_r$. Values for $V < -30$ mV were estimated from Pr5, values for -40 mV and upwards from Pr4. To see this figure in color, go online.

This can be rewritten as a "Boltzmann curve"

$$a_\infty(V) = \frac{1}{1 + e^{(s_a h_a - s_a V)}} = \frac{1}{1 + e^{s_a(h_a - V)}}, \tag{12}$$

where h_a is the "midpoint of activation" (the point at which $a_\infty(V = h_a) = 0.5$) and s_a is the slope of the activation curve at $V = h_a$. Assuming $\tilde{a}_\infty(V) \approx a_\infty(V)$, values for h_a and s_a can then be obtained numerically (optimizing on sum of square error) or by plotting $\tilde{a}_\infty$ vs. V and reading the values from the graph. At this stage we have an equation for $a_\infty(V)$ and two constraints on the parameters p_1, p_2, p_3, p_4, namely

$$s_a = p_2 + p_4, \tag{13}$$

$$h_a = \frac{\log p_3 - \log p_1}{s_a}. \tag{14}$$

Next, we rewrite Eq. 6 to find

$$a_\infty(V) \, / \, \tau_a(V) = k_1(V) = p_1 e^{p_2 V}, \tag{15}$$

which allows us to infer values for p_1 and p_2 by plotting the quantity $a_\infty / \tilde{\tau}_a$ vs. V (using the fitted equation for $a_\infty(V)$ rather than the measured values $\tilde{\boldsymbol{a}}_\infty$ to reduce noise) and fitting an exponential curve. Note that because $a_\infty \approx 0$ for values below -60 mV, any data points for low voltages will contribute very little to the final fit. As a simple alternative, we can plot the quantity $\log(a_\infty / \tilde{\tau}_a)$ and fit with a linear equation $\log(p_1) + p_2 V$ (note that this is equivalent to doing a graphical fit using semilogarithmic graph paper); this provides us with more reliable values for p_1 and p_2, after which we can use $p_4 = s_a - p_2$ and $p_3 = p_1 e^{s_a h_a}$ to find the remaining activation parameters.

Using a similar procedure (but with a slight change in signs), we plot the logarithm of $k_4(V) = r_\infty / \tilde{\tau}_r = p_7 e^{-p_8 V}$, fit to find p_7 and p_8, and then use $p_6 = -s_r - p_8$ and $p_5 = p_7 e^{s_r h_r}$ to find all four inactivation parameters.

Finally, we find a value for the conductance parameter g_{Kr} by performing a simulation of Pr5, deriving an IV curve, and then calculating the scaling factor that minimizes the sum-of-squares error between the simulated and experimental IV curves.

Quantifying goodness of fit

To evaluate the goodness of fit from method 1, we derive an error function and evaluate it with the obtained parameter values. We write $\boldsymbol{\theta} = \{p_1, p_2, \ldots, p_9\}$ for the parameters and introduce symbols representing the experimental and measured data sets. To denote a cell's set of experimentally approximated midpoints of activation, we use $\tilde{\boldsymbol{a}}_\infty^{\mathrm{cell}}$ while $\boldsymbol{a}_\infty^{\mathrm{model}}$ is used to indicate the value of the model variable (a_∞), evaluated at the same voltages. Similar notation is used for the midpoint of inactivation and both time constants. Next, we define the root mean-squared error (RMSE) between two data sets $\boldsymbol{x}$ and $\boldsymbol{y}$ as

$$R(\boldsymbol{x}, \boldsymbol{y}) = \sqrt{\frac{1}{n} \sum_{i=1}^{n} (x_i - y_i)^2}, \tag{16}$$

where both data sets have equal length n. Using this notation, we can write the RMSE between experimental results $\tilde{\boldsymbol{a}}_\infty^{\mathrm{cell}}$ and model values $\boldsymbol{a}_\infty^{\mathrm{model}}$ as $R(\tilde{\boldsymbol{a}}_\infty^{\mathrm{cell}}, \boldsymbol{a}_\infty^{\mathrm{model}})$. With this notation, we can now define the error criterion as a weighted sum of RMSEs:

$$E_{\mathrm{M1}}(\boldsymbol{\theta}) = R\left(\tilde{\boldsymbol{a}}_\infty^{\mathrm{cell}}, \boldsymbol{a}_\infty^{\mathrm{model}}\right) + R\left(\tilde{\boldsymbol{r}}_\infty^{\mathrm{cell}}, \boldsymbol{r}_\infty^{\mathrm{model}}\right) + \frac{R\left(\tilde{\boldsymbol{\tau}}_a^{\mathrm{cell}}, \boldsymbol{\tau}_a^{\mathrm{model}}\right)}{\max \tilde{\boldsymbol{\tau}}_a^{\mathrm{cell}}} + \frac{R\left(\tilde{\boldsymbol{\tau}}_r^{\mathrm{cell}}, \boldsymbol{\tau}_r^{\mathrm{model}}\right)}{\max \tilde{\boldsymbol{\tau}}_r^{\mathrm{cell}}}, \tag{17}$$

where $n_{a_\infty} = 7$, $n_{r_\infty} = 8$, $n_{\tau_a} = 9$, and $n_{\tau_r} = 17$. Note that this is a cell-specific measure because both time-constant terms are normalized with respect to the largest value found in the cell data used. This weighting corrects for differences in the scaling of the four RMSEs, ensuring that none of them

dominate in the end result. No normalization is needed for the steady states, which are already constrained to the range [0, 1]. In contrast to measures introduced below, E_{M1} is invariant with respect to conductance (p_9). Finally, we note that an alternative method 1 could be created by using numerical optimization to minimize E_{M1}; this is explored further in Supporting Materials and Methods, Section S3.6, but did not create a more predictive model than the method presented here.

Method 2: Fitting simulated summary curves

In method 2, we accept that the summary curves are imperfect approximations of the model variables, and so we base our fitting on simulated experiments of Pr2–5, analyzed using the same methods as for the experimental data to arrive at simulated versions of the summary statistic curves. This gives us two sets of summary curves, one simulated and one experimental, using which we can define an error measure that quantifies the goodness of fit. By varying the parameters and repeating the simulations, we can then find a set of parameters that minimizes this error.

To formulate the error measure, we again write $\tilde{\boldsymbol{a}}_\infty^{\text{cell}}$ to denote a cell's set of experimentally approximated midpoints of activation, and we introduce the notation $\tilde{\boldsymbol{a}}_\infty^{\text{sim}}$ for its simulated counterpart (note that we use a tilde notation here to indicate that $\tilde{\boldsymbol{a}}_\infty^{\text{sim}}$ is not a model variable but a result derived from a simulated experiment). In this measure, we use the IV curve rather than the steady state of inactivation because 1) it contains the same data points (albeit with a different scaling); 2) unlike $\tilde{\boldsymbol{r}}_\infty^{\text{cell}}$, it does not suffer from numerical issues near $V = E_K$ (see Supporting Materials and Methods, Section S1.5.1); and 3) it contains information about g_{Kr}, which is lacking from the other summary curves. Similar symbols denote the remaining summary curves: experimental midpoint of inactivation ($\tilde{\boldsymbol{r}}_\infty^{\text{cell}}$), time constant of activation ($\tilde{\boldsymbol{\tau}}_a^{\text{cell}}$), time constant of inactivation ($\tilde{\boldsymbol{\tau}}_r^{\text{cell}}$), and the IV curve from Pr5 ($\mathbf{IV}^{\text{cell}}$).

The error to minimize for each cell is defined as a weighted sum of RMSEs

$$E_{M2}(\boldsymbol{\theta}) = R\left(\tilde{\boldsymbol{a}}_\infty^{\text{cell}}, \tilde{\boldsymbol{a}}_\infty^{\text{sim}}\right) + \frac{R\left(\tilde{\boldsymbol{\tau}}_a^{\text{cell}}, \tilde{\boldsymbol{\tau}}_a^{\text{sim}}\right)}{\max \tilde{\boldsymbol{\tau}}_a^{\text{cell}}} + \frac{R\left(\tilde{\boldsymbol{\tau}}_r^{\text{cell}}, \tilde{\boldsymbol{\tau}}_r^{\text{sim}}\right)}{\max \tilde{\boldsymbol{\tau}}_r^{\text{cell}}} + \frac{R\left(\mathbf{IV}^{\text{cell}}, \mathbf{IV}^{\text{sim}}\right)}{\max \mathbf{IV}^{\text{cell}} - \min \mathbf{IV}^{\text{cell}}}. \tag{18}$$

The number of data points was the same for each cell, with $n_{\tau_a} = 9$, $n_{\tau_r} = 17$, $n_{a_\infty} = 7$, and $n_{IV} = 9$ (for a total of 42 cell-specific data points). As in E_{M1}, weighting is used here to give every term equal influence.

Method 3: Fitting current traces from traditional protocols

In method 3, we forgo the summary-curve calculation altogether and simply perform "whole-trace fitting" on the currents resulting from the "traditional protocols" Pr2–5. Writing $\boldsymbol{I}_i^{\text{cell}}$ for the current recorded from protocol i, and $\boldsymbol{I}_i^{\text{sim}}(\boldsymbol{\theta})$ for the simulated current in protocol i with parameters $\boldsymbol{\theta}$, we define the function to be optimized as a normalized RMSE:

$$E_{M3}(\boldsymbol{\theta}) = \sum_{i=2}^{5} \frac{R\left(\boldsymbol{I}_i^{\text{cell}}, \boldsymbol{I}_i^{\text{sim}}\right)}{\max \boldsymbol{I}_i^{\text{cell}} - \min \boldsymbol{I}_i^{\text{cell}}}. \tag{19}$$

Note that the weighting here is not strictly necessary for the optimization procedure but is used to enable E_{M3} value comparisons between cells.

Method 4: Fitting current traces from an optimized protocol

In method 4, we define a similar normalized RMSE measure based on fitting only the current under the sinusoidal protocol, Pr7:

$$E_{M4}(\boldsymbol{\theta}) = \frac{R\left(\boldsymbol{I}_7^{\text{cell}}, \boldsymbol{I}_7^{\text{sim}}\right)}{\max \boldsymbol{I}_7^{\text{cell}} - \min \boldsymbol{I}_7^{\text{cell}}}. \tag{20}$$

As in E_{M3}, the weighting used here allows us to compare values between cells.

Validation and cross comparison

To compare the results of the four fitting methods, we applied each method to each cell, resulting in four parameter sets per cell. Next, we simulated the AP waveform protocol (Pr6) with all parameter sets and compared these to the corresponding measurements. Note that Pr6 was not used in any of the fitting methods, so this constitutes an independent validation. The results were quantified using a normalized RMSE:

$$E_{AP}(\boldsymbol{\theta}) = \frac{R\left(\boldsymbol{I}_6^{\text{cell}}, \boldsymbol{I}_6^{\text{sim}}\right)}{\max \boldsymbol{I}_6^{\text{cell}} - \min \boldsymbol{I}_6^{\text{cell}}}, \tag{21}$$

where R is defined by Eq. 16, as before.

In addition to the independent validation, we performed cross-validation by testing how well models fit with one method could predict the fitting data used by the others. This was shown visually and was quantified by evaluating E_{M1}, E_{M2}, E_{M3}, and E_{M4} on the best result found for each cell/method.

Multicell measures of the fitting methods' performance were defined as the mean average of the error measure over all nine cells. Writing $E_{AP,k}$ for the RMSE in cell k on the AP signal, we defined

$$E_{AP,all} = \frac{1}{9} \sum_{k=1}^{9} E_{AP,k}. \tag{22}$$

Note that $E_{AP,k}$ already contains a term to normalize the error with respect to the magnitude of the current in cell k so that no further weighting is required. Combined error measures for E_{M1} through to E_{M4} were defined in the same manner.

Minimizing the error measures

Methods 2–4 all proceed by finding a parameter set that minimizes an error function. In previous work, we found that the global optimization algorithm CMA-ES (73) provided good fits for a range of models from the ion channel to cell scale (20,74–77), and was both fast and behaved reproducibly (returning the same answer when started from different initial guesses in the parameter space). For methods 2–4, we used a CMA-ES population size of 10 and halted the optimization only when the objective function changed by less than 10^{-11} per iteration for 200 successive iterations. Other important aspects of making our optimization strategy reliable were 1) placing physiologically inspired bounds on the parameter space, 2) searching (and choosing starting points) in a log-transformed space, 3) reducing solver tolerances to eliminate numerical noise in simulation output, and 4) testing reliability by running repeated fits from different starting points.

Constraints on the parameter space were set as in Beattie et al. (20) and included upper and lower bounds for the parameters p_1 to p_9 but also restricted the value of the reaction rates k_1 to k_4. The resulting boundaries are visualized in Fig. 3, *A* and *B*. Details of how the boundaries were defined are given in Supporting Materials and Methods, Section S2.2. To implement these constraints, points outside the boundaries were automatically assigned an error of ∞ during optimization.

Several studies (78–80) have found that for strictly positive parameters (i.e., for all nine parameters in our model), optimization performance can be improved by searching in a log-transformed parameter space. In at least some cases, this has been shown to turn nonconvex (i.e., hard) problems into simpler convex ones (81). For methods 2–4, we used a log transform

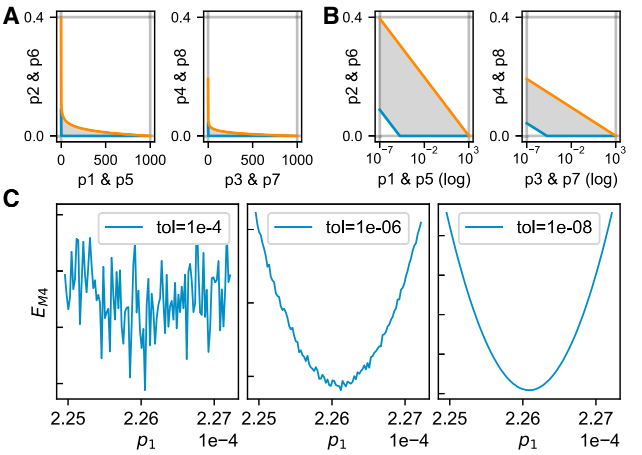

FIGURE 3 Boundaries and transforms on the parameter space and the effect of solver tolerances. (*A*) Constraining the transition rates k_1–k_4 (over physiologically relevant voltages) to relevant timescales creates a two-dimensional boundary on each parameter pair (Supporting Materials and Methods, Section S2.2). The gray region indicates valid samples, with an upper rate constraint in orange and a lower rate constraint in blue. The horizontal and vertical gray lines indicate the lower and upper bounds for the parameters p_1–p_8. (*B*) The same parameter constraints with a log transform on one of the parameters are shown. Note this increases the size of the feasible region for off-the-shelf optimizers that know nothing about the problem, and we later see the objective function becomes reasonably convex and symmetric under this transform (see Fig. 11). (*C*) Using default tolerances of adaptive solvers can lead to numerical noise in the function being optimized. This can be remedied by selecting lower tolerances. To see this figure in color, go online.

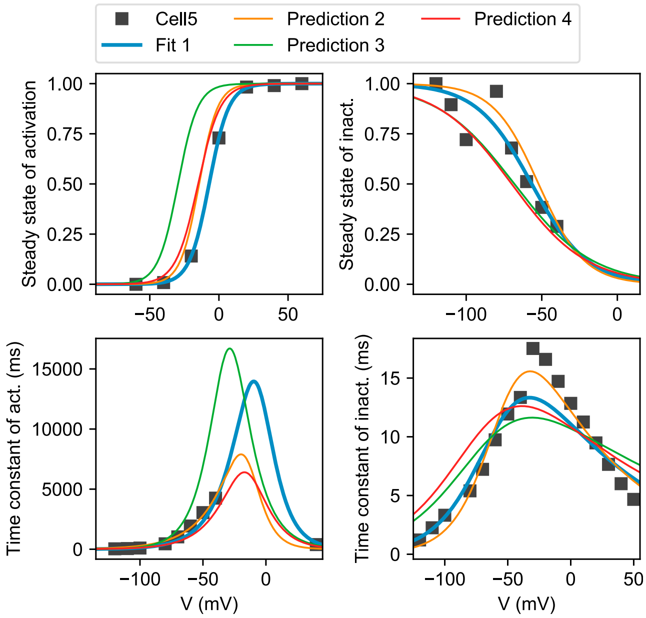

FIGURE 4 Method 1 goodness of fit and cross-validation on cell #5. Experimental approximations of the steady states ($\tilde{a}_\infty$ and $\tilde{r}_\infty$) and time constants ($\tilde{\tau}_a$ and $\tilde{\tau}_r$) are shown, derived from measurements in cell #5. The model curves for $a_\infty(V)$, $r_\infty(V)$, $\tau_a(V)$, and $\tau(V)$ are shown for models fitted with each of the four methods. Note that only method 1 was trained on this data, making this a goodness-of-fit figure for method 1, whereas for the other methods, this figure shows a prediction. To see this figure in color, go online.

on parameters p_1, p_3, p_5, and p_7. As Fig. 3 *B* shows, the boundaries on these parameters allowed them to vary over 10 orders of magnitude, so a log transform seemed most appropriate. The effects of this strategy are further explored in Supporting Materials and Methods, Section S2.4.

We used an adaptive step time solver for simulations of Pr6 and Pr7. As shown in Johnstone (82), simulating with lax tolerance settings can lead to (seemingly random) fluctuations in the error measure for nearby values of $\boldsymbol{\theta}$, which has the effect of creating several local minima in otherwise smooth regions. To combat this, we set absolute and relative error tolerances of 10^{-8}. This is shown in Fig. 3 *C*. For the voltage-step protocols Pr2–5, we circumvented the issue entirely by using the model's analytical solution for fixed voltages to calculate the time course at each step.

When running the optimizations for methods 2–4, each fit was run several times from different starting points, sampled uniformly from within the transformed space shown in Fig. 3 *B*. Fifty repeats were run per cell for methods 3 and 4 while 80 repeats per cell were run for method 2. The best result (lowest error) was used as the final fit for each cell/method. We comment on the reliability of these fits below in the Reliability and Performance part of the Results.

We tested our methods by performing a study on synthetic data, described in Supporting Materials and Methods, Section S2.3, based on parameters given in Beattie et al. (20) and Gaussian noise comparable to the strongest noise encountered in our data set. In this in silico study, we saw that our methods reliably found a point near the known true solution, that solutions found by repeated fits had very similar parameter values, and that models fitted to one data set provided good predictions on the other data sets.

Software and algorithms

Simulations were performed in Myokit (83), using CVODE (84) for Pr6 and Pr7 simulations with tolerances as described above and an analytical solver for Pr2–5. Further analysis was performed in Python 3.7 using NumPy/SciPy (85). When deriving experimental time constants, fits to exponential curves were performed using the Nelder-Mead downhill simplex algorithm implemented in SciPy. All other fits were performed using CMA-ES (73) via the PINTS (86) inference framework. When calculating benchmarks, methods 2–4 were run on a machine with 24 Intel Xeon 2.2 GHz CPU cores (48 with hyperthreading), with four optimizations running concurrently at all times. All data, code, results, and figures are available to download from https://github.com/CardiacModelling/FourWaysOfFitting. A permanently archived version is available on Zenodo at https://doi.org/10.5281/zenodo.3378030.

RESULTS

We now discuss the results of fitting a model with each of the four methods, using cell #5 as an example in our figures. Figures for all nine cells can be found online at https://github.com/CardiacModelling/FourWaysOfFitting. For each fit, we discuss the quality of fit but also investigate whether models made with the other methods do well at predicting the methods' fitting data.

In method 1, we equate model variables (e.g., a_∞) with experimentally derived values (e.g., $\tilde{a}_\infty$). Fig. 4 shows the experimentally derived values as black squares, whereas the blue line (fit 1) represents the quality of fit obtained by method 1. Note that these lines are plotted directly from Eqs. 5 and 6 and do not involve simulation. Similar lines are shown for models fit with methods 2–4, labeled

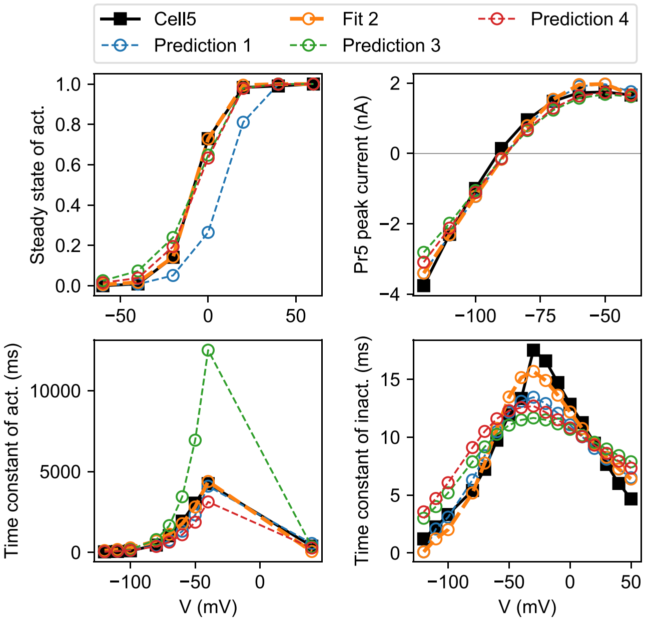

FIGURE 5 Method 2 goodness of fit and cross-validation on cell #5. The experimental steady state of activation, the IV curve, and both experimental time constants are shown. By simulating the protocols and performing the same analysis, similar summary curves were derived for each of the four methods. The fit for method 2 is shown, along with the predictions from models made with methods 1, 3, and 4. To see this figure in color, go online.

as "predictions" in the figure. As Fig. 4 shows, the lines drawn from the method 1 model fit the data well, whereas the models fitted through simulation (methods 2–4) show a notable mismatch for both steady states and time constants. However, because the summary curves approximate but do not equal the model variables, a perfect fit in this figure is not necessarily desirable. This is discussed further in Supporting Materials and Methods, Section S1.6.

In Fig. 5, we again plot the experimentally derived data points (e.g., $\tilde{\boldsymbol{a}}_{\infty}^{\text{cell}}$), but instead of comparing them to model variables, we compare them to simulation results (e.g., $\tilde{\boldsymbol{a}}_{\infty}^{\text{sim}}$). Method 2 attempts to minimize the discrepancy between the two and achieves this well (fit 2 in Fig. 5). By contrast, the predictions from a model made with method 1 now show a clear shift in the steady state of activation curve. Interestingly, only the method-2-derived model shows a close fit to the experimental time-constant data, although none of the methods are able to match the steep peak in the $\tilde{\boldsymbol{\tau}}_r$ points well, which may be due to the fact that points left of the peak were derived from Pr5, while the rightmost points were obtained from Pr4.

Fig. 6 shows selected portions of the currents elicited by Pr2–5. Method 3 minimizes the discrepancy between simulated and measured currents and provides a good fit (fit 3 in the figure), although some differences can still be seen. Models made with methods 1 and 2 do not generally predict the observed currents well, although the qualitative behavior is correct in all cases. Models with method 4 provide better predictions here, although interestingly, only method 3 gets the slope of the Pr3 currents during the P2 step right, indicating a difference in the deactivation properties of method 3 parameters compared to the others. The negative currents in Pr4 appear challenging for all methods, whereas method 3 and 4 results match well on the higher potential steps.

Next, we inspected the capability of the different models to predict the currents from the sine wave protocol (Pr7), as shown in Fig. 7. The method 4 model obtained a good fit to the data, although some differences can be seen in the deactivating part of the initial voltage step. Predictions from the model made with method 3 were relatively good, whereas models fitted with method 1 and in particular method 2 performed poorly at predicting Pr7 currents.

Figs. 4, 5, 6, and 7 showed that all four methods achieved good fits, judged by their own criteria, whereas the quality of predictions outside of the fitting data varied. The AP waveform is an attempt to test predictions for the most physiologically relevant I_{Kr} behavior. A visual comparison of predictions made with models from each of the four methods is shown in Fig. 8, again with the cell #5 results used as an example. In this cell, the predictions from method 2 were generally poor, whereas methods 3 and 4 provide much better predictions.

A quantitative view of the validation and cross-validation results for cell #5 is given in Fig. 9 (*top*). The top row of this table shows the RMSE for the independent validation protocol. To enable easier comparisons, the RMSEs have been normalized to the best performing method so that the best method has relative RMSE 1, whereas a method with a relative value of 2 has an RMSE that is twice as high. For cell #5, method 4 provided the best predictions of the independent test protocol and also performed well on cross-validation. As may be expected, each method had the lowest score on its own fitting data. Data for all nine cells are given in Supporting Materials and Methods, Section S3.3.

The lower panel in Fig. 9 shows similar relative RMSEs but now presented as the mean and SD for all nine cells. Here, method 3 performed best at the AP prediction task, although method 4 was very close (within SD) and outperformed it at cross-validation.

Interestingly, method 2 outperformed method 1 on the E_{M1} criterion in the averaged data and also in six out of nine cells. This indicates the advantage of fitting steady states and time constants simultaneously (as happens in method 2) over fitting them sequentially (method 1), as previously described (33,35). Further illustration is provided in a figure in Supporting Materials and Methods, Section S3.6, in which we show that small changes in the slope of the steady-state curve have a strong effect on the time constants derived by method 1; method 2 can use this to its advantage by slightly adjusting the model's steady-state curve slope to obtain a better fit to experimental time-constant data.

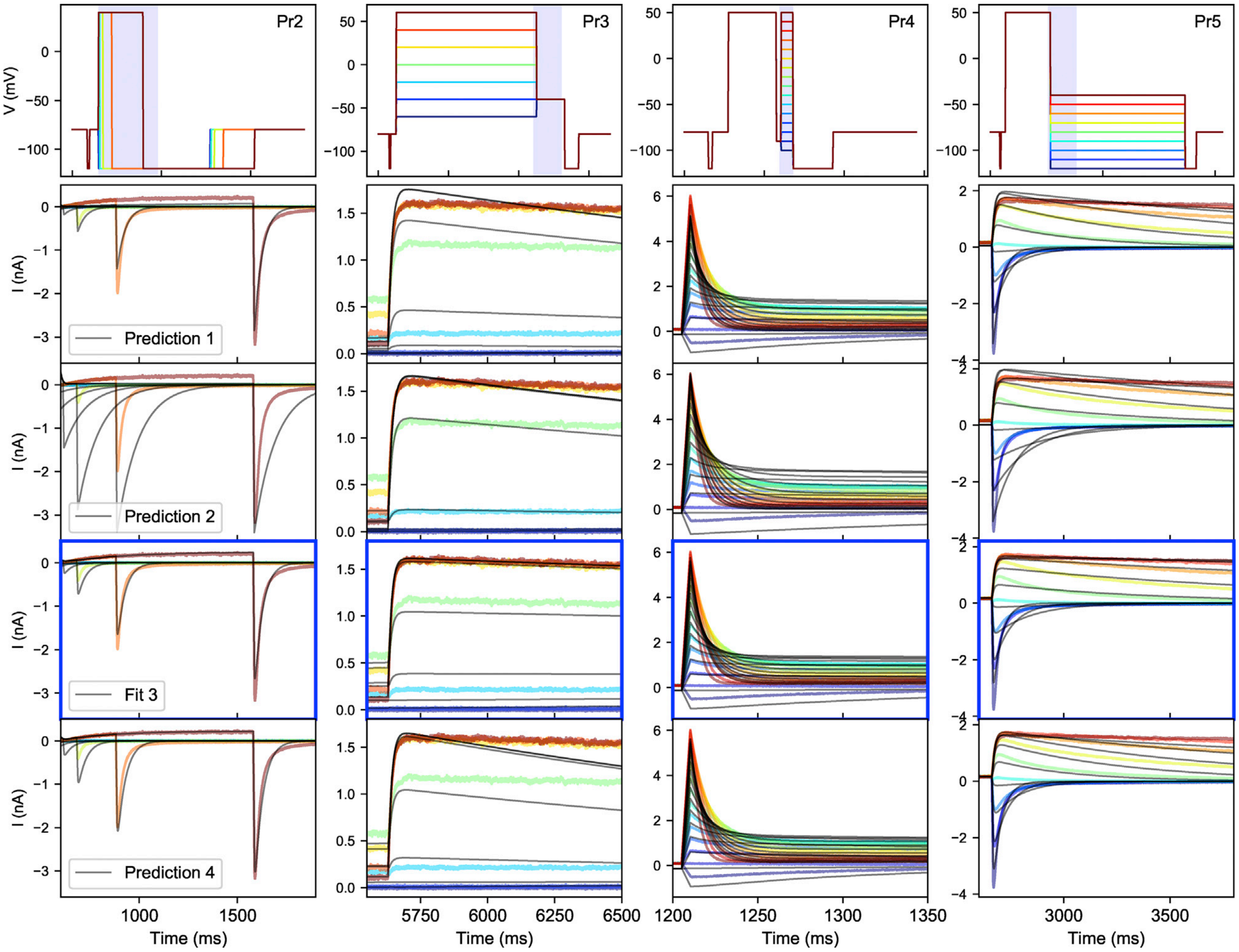

FIGURE 6 Method 3 goodness of fit and cross-validation on cell #5. The top row shows the voltage protocols Pr2–5, with different colors used for each sweep. The same colors are used in each of the panels below to show the corresponding experimental data, whereas simulation results are shown in black. In the top row, the full time span of each protocol is shown, but the plots shown below each zoom in on a specific region, indicated by the shaded area in the top row. The fit made with method 3 is highlighted with blue borders (fit 3) while the other rows show predictions from models made with methods 1, 2, and 4. To see this figure in color, go online.

Reliability and performance

Having inspected the predictive capabilities and quality of fit of models obtained with each method, we next investigated their reliability. Ideally, a method returns the same result every time it is applied, and indeed, method 1 lives up to this ideal. For methods 2–4, however, we used 1) randomly sampled initial guesses for parameter sets and 2) a stochastic optimizer. To increase our chances of finding the best result, we repeated this process 50 times for methods 3 and 4 and 80 times for method 2. For a reliable method, we expect a large number of the methods to return similar parameter sets, with similar RMSEs.

Fig. 10 gives an indication of each method's reliability. It shows that method 3 returned a result similar to the best one found on 50–84% of the repeats, depending on the cell. Method 4 appears slightly more reliable, with numbers ranging from 60 to 94%. For both these methods, results with a low RMSE value all had parameters clustered in a small area of the parameter space. For method 2, only a very small number of repeats returned a result with an RMSE similar to the lowest one found. In addition, the method 2 results with the lowest RMSE values were not always close together in parameter space. Method 1 is a deterministic method and so was omitted from this figure. This is further explored in Supporting Materials and Methods, Section S2.3.2.

To investigate the performance of the different methods, we measured the computation time for each method and counted the number of forward simulations that were performed during an optimization. On average, method 2 was much slower than method 3, which itself was slower than method 4 (shown in Supporting Materials and Methods, Section S3.4). The number of function evaluations was similar for methods 3 and 4, indicating that

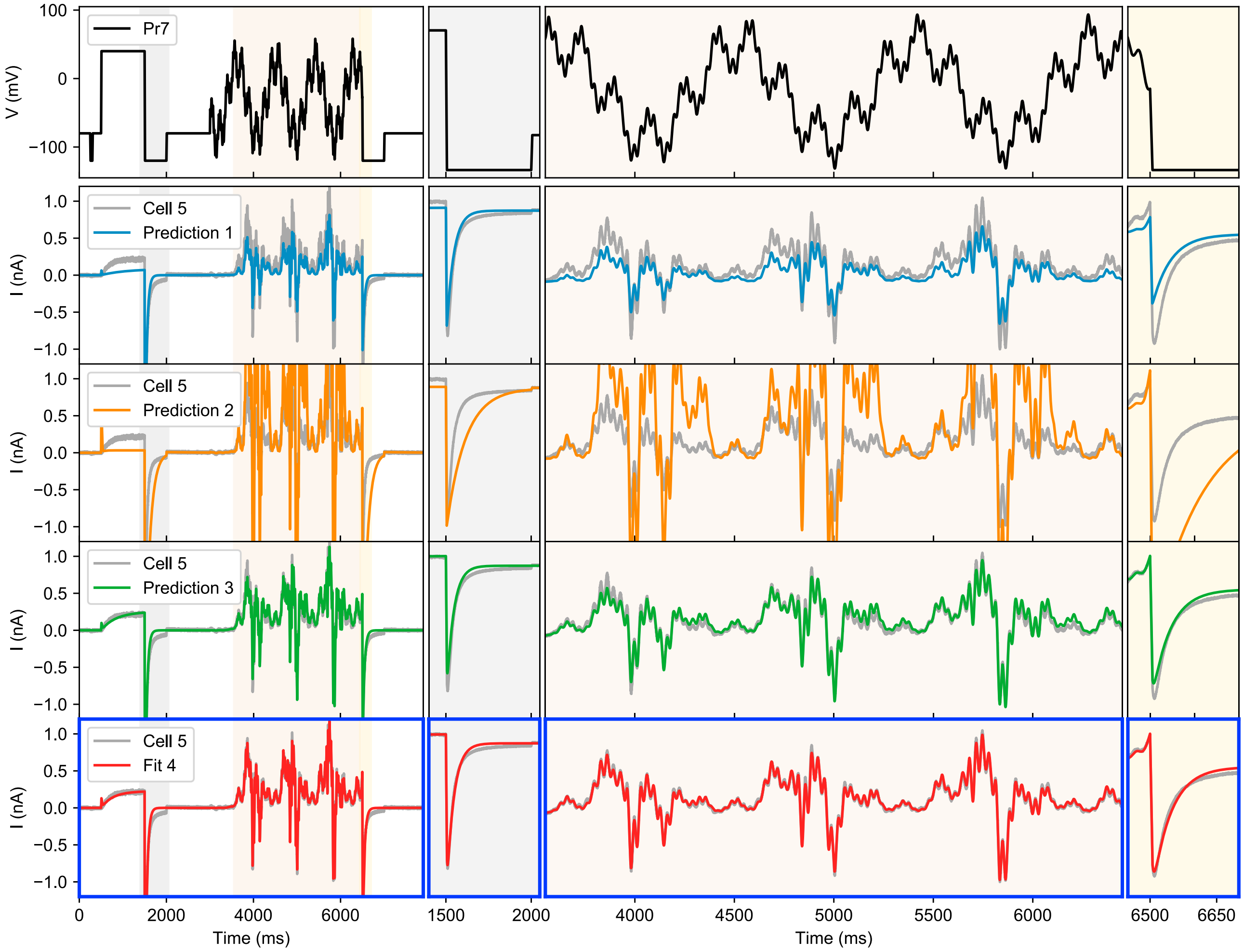

FIGURE 7 Method 4 goodness of fit and cross-validation on cell #5. The top panel shows the voltage protocol (Pr7) while the three panels below show the same current recording (*gray line*), and each row shows a prediction from a model made with methods 1–3, respectively (*colored lines*). The bottom panel, highlighted with blue borders, shows the fit for method 4 (fit 4). To see this figure in color, go online.

this difference in performance was due to the increased simulation time needed for method 3 (228 s of simulation for method 3 vs. 8 s of simulation for method 4). However, many repeats of method 2 were seen to terminate with a low number of evaluations, which may indicate these optimizations terminated early in a local optimum. To explore this hypothesis, we performed a brute-force exploration of E_{M2} and E_{M4} for cell #5 in the region near the optimum returned by methods 2 and 4, respectively, as shown in Fig. 11. For E_{M4}, we see a clear optimum in each panel, and the function appears smooth (at least within the optimization boundaries, indicated by the *white lines*). For E_{M2}, however, a lot of discontinuities can be seen. The darkest areas in these panels are regions where the intermediate analysis to derive time constants and steady states from the simulated experiments failed. As the rightmost panels show, this can occur in otherwise smooth parts of the slices, which may prove challenging for optimization routines. In addition, a lot of "noise" can be seen (e.g., in the *green areas* of the first panel), which may indicate the presence of many local minima—however, each panel is a two-dimensional slice of a nine-dimensional space, so this is not necessarily the case.

DISCUSSION

We defined four methods, each representative of a wider class, to fit ion current models using whole-cell current recordings. Methods 3 and 4, both based on whole-current fitting, were found to provide the most accurate predictions, whereas methods 1 and 2, both based on fitting preprocessed "summary" data, fared poorly. Of the methods in which we applied a stochastic optimization routine (2–4), methods 3 and 4 were found to provide the most consistent results, and method 4 was the most time-efficient both in terms of experimental and computational effort.

To further compare the results from different methods, we plotted the fits from each method and each cell in Fig. 12.

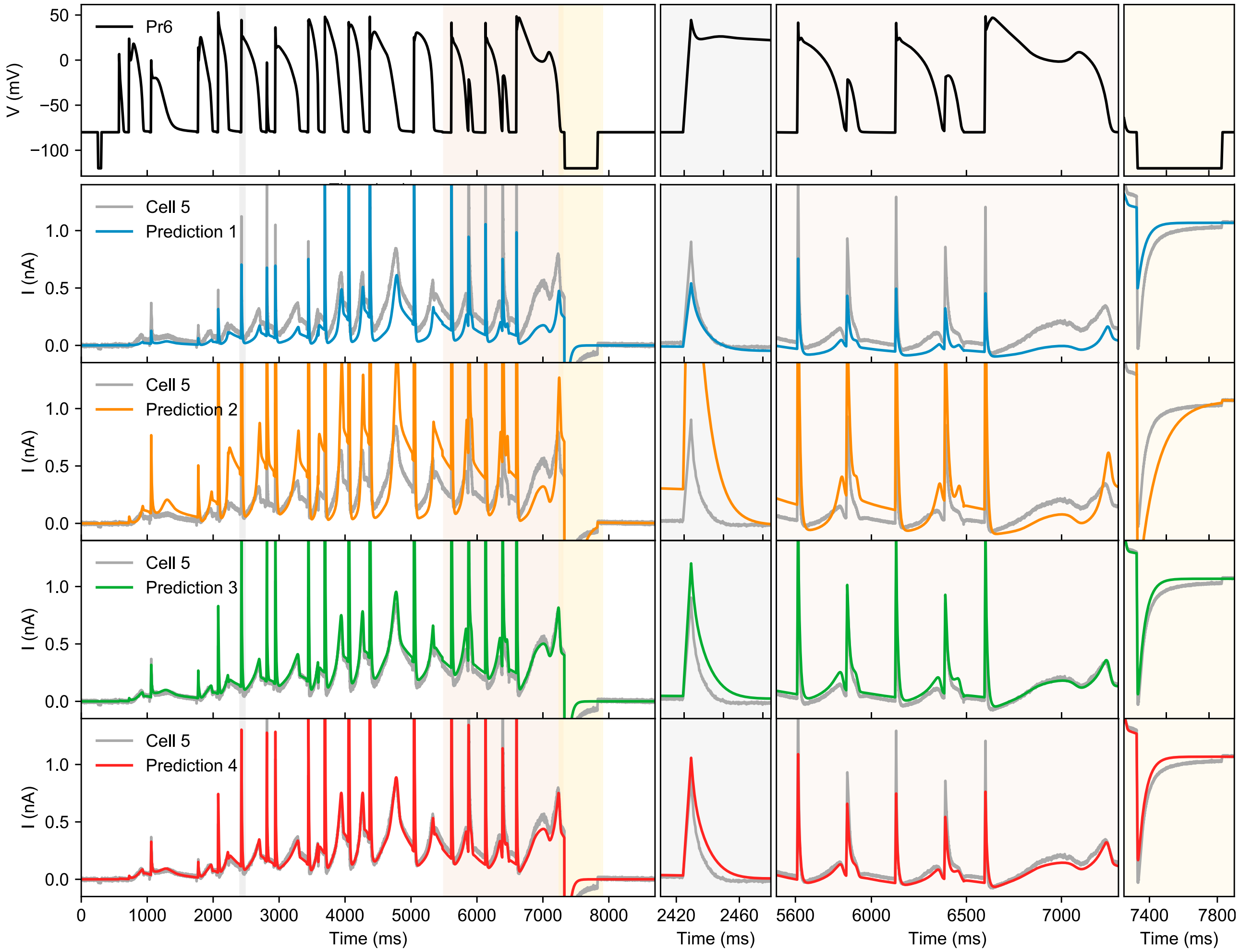

FIGURE 8 Validation on the AP waveform signal (Pr6). The voltage protocol is shown on the top row, with subsequent rows colored traces showing predictions from methods 1–4, respectively. The current measured in cell #5 in response to Pr6 is shown in gray (same in each row). None of the models were trained on Pr6 data, making this an independent validation. The left panels show the full duration of the signal, and the panels on the right zoom in on selected regions (2.41–2.48, 5.5–7.3, and 7.25–7.9 s). To see this figure in color, go online.

Even in areas where parameter sets overlap, each method can be seen to introduce its own small bias. The figure also points to a difference in deactivation for method 3, which consistently placed the deactivation parameters p_3 and p_4 in a different part of the parameter space than the other methods. We found that models using parameter sets found by method 3 gave the best AP predictions, although this was very closely followed by method 4 (see Fig. 9). Looking at Fig. 6, we can see that many cases in which method 1, 2, and 4 predictions deviated from the measured current were during deactivation. This points to an advantage in describing deactivation for method 3 and suggests an area in which future versions of the sinusoidal protocol used in method 4 could be improved. In particular, the slow timescale of activation and deactivation may necessitate the inclusion of long steps similar to those in Pr3/Pr5 or the superposition of a lower frequency sine wave.

For methods 2–4, we observed significant improvements to the performance of optimization routines by performing a log transform on some of the parameters, providing appropriate constraints on rates and voltage-dependence of the rates, and refining differential equation numerical solver tolerances. At least some of these aspects may address previous findings that whole-trace fitting is difficult (58), as we found very consistent results with an "off-the-shelf" optimizer after adopting these simple approaches.

The optimization surface for method 2 is considerably more ragged than methods 3 and 4, making this a difficult optimization problem. As method 2 is very common in the literature on ionic model fitting, this result has some implications for the cardiac modeling community: its reputation as a hard problem requiring special methods may be due to the choice of summary data rather than any intrinsic mathematical or computational difficulty with the models. Since the choice of method 2 is typically based on data availability (48), increased sharing of whole-cell current data is a more promising way forward than developing and applying novel optimization algorithms. For experimenters, as well as

Cell 5	Method 1	Method 2	Method 3	Method 4
AP validation	1.82	4.25	1.29	1.00
Method 1 RMSE	1.00	1.11	4.64	1.99
Cross-validation M2	2.58	1.00	6.33	2.48
Cross-validation M3	2.46	3.88	1.00	2.13
Cross-validation M4	3.78	13.54	1.70	1.00

All cells	Method 1	Method 2	Method 3	Method 4
AP validation	1.7 (0.3)	1.9 (0.8)	1.0 (0.1)	1.1 (0.2)
Method 1 RMSE	1.2 (0.5)	1.0 (0.2)	3.3 (0.6)	1.9 (0.2)
Cross-validation M2	2.6 (0.7)	1.0 (0.1)	4.8 (1.0)	2.9 (0.4)
Cross-validation M3	1.7 (0.4)	1.8 (0.4)	1.0 (0.4)	1.7 (0.4)
Cross-validation M4	2.8 (0.9)	3.8 (1.8)	1.8 (0.5)	1.0 (0.3)

FIGURE 9 Validation and cross-validation results. (*Top*) Results for cell #5 are shown. Each row shows the relative RMSE for a fitting method, scaled so that the best performing method is indicated by 1, whereas a method with a relative score of, e.g., 1.2 had an RMSE that was 1.2 times larger. The top row shows the relative E_{AP} for each method, with the remaining rows showing E_{M1}, E_{M2}, E_{M3}, and E_{M4}, respectively. (*Bottom*) Mean results over all nine cells are shown, with SDs given in parentheses. To see this figure in color, go online.

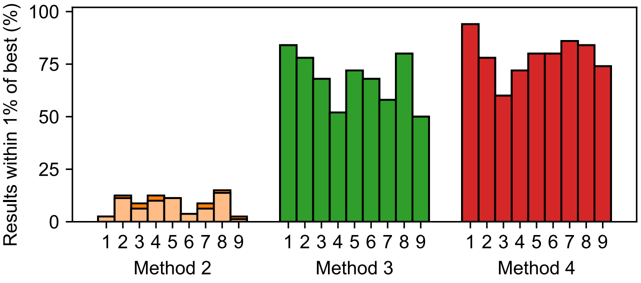

FIGURE 10 The percentage of optimizations from different starting points that found the "best result." Darker bars in the background indicate the percentage of optimizations that returned a result with an RMSE within 1% of the best result found. The lighter bars in the foreground indicate the percentage that was also close in parameter space, (such that no single parameter varied by more than 1% from the best result found). Note that for methods 3 and 4, the bars overlap exactly, but the method 2 results for some cells contain "'best" points with similar low RMSEs but different parameter values. To see this figure in color, go online.

universities, journals, and other publishers of scientific data, our findings re-emphasize the need for sharing of raw data, for example, in online repositories or data supplements (87).

In cases in which full currents are not available (i.e., when using historical data), some steps may be taken to alleviate the problems associated with method 2. Reviewing Fig. 11, some of the high error parts of the E_{M2} surface arise from numerical issues during the derivation of summary curves from simulated experiments (for instance, in deriving time constants from almost-flat current traces). It may be possible to standardize and improve these analysis methods and so remove some of the discontinuities in E_{M2}.

Another approach we tried to improve methods 2 and 3 is to use method 1 to propose an initial guess parameter set for methods 2 and 3 instead of sampling a point from randomly within the parameter constraints, as hinted at previously (88). In initial tests, this gave very similar results to the "full" methods but with only a single optimization repeat needed. The full results are shown in Supporting Materials and Methods, Sections S3.7 and S3.8.

A reason sometimes given to continue using experimental steady-state and time-constant curves as a summary of current kinetics is that they allow comparison to published data and previous results from the same lab. So, method 3 may be seen to have an advantage over method 4 in that it contains the traditional protocols needed to derive these curves. However, as Vandenberg et al. (89) point out, it is important to realize that these values can be highly sensitive to the (occasionally unpublished) details of the protocols and analysis methods so that making these comparisons is not without danger. We have seen, though, that method 4 can provide excellent simulations of these protocols, so comparisons to previous data could be made by 1) running an experiment with a novel optimized protocol, 2) quickly and reliably fitting a model to the raw current data, and 3) simulating the previously used protocols and performing the analysis. Indeed, this method could be used to compare data sets (via modeling) from protocols with slight discrepancies (90).

Limitations and further work

In this work, we characterized four methods of fitting an ion current model and proceeded to analyze and critique these methods based on our characterization. Although we tried not to misrepresent any method, it is therefore important to highlight any areas in which our efforts may become subjective or otherwise fall short.

Firstly, running both a full set of conventional protocols, as well as Pr6 and Pr7, in a single cell is experimentally infeasible. For that reason, the study by Beattie et al. (20) used truncated version of the conventional protocols (e.g., with fewer voltage sweeps) and omitted Pr2 variants with different P1 voltages. We must therefore admit the possibility that methods 1 or 2, performed with a larger data set, would lead to improved results. In particular, the experimental time constants of activation are "missing" in the range (−30 mV, 30 mV), which is exactly where the peak value should occur. However, given the excellent performance of method 3 in our study, it seems the required information was present in the recordings, so the limited efficiency of both methods still stands.

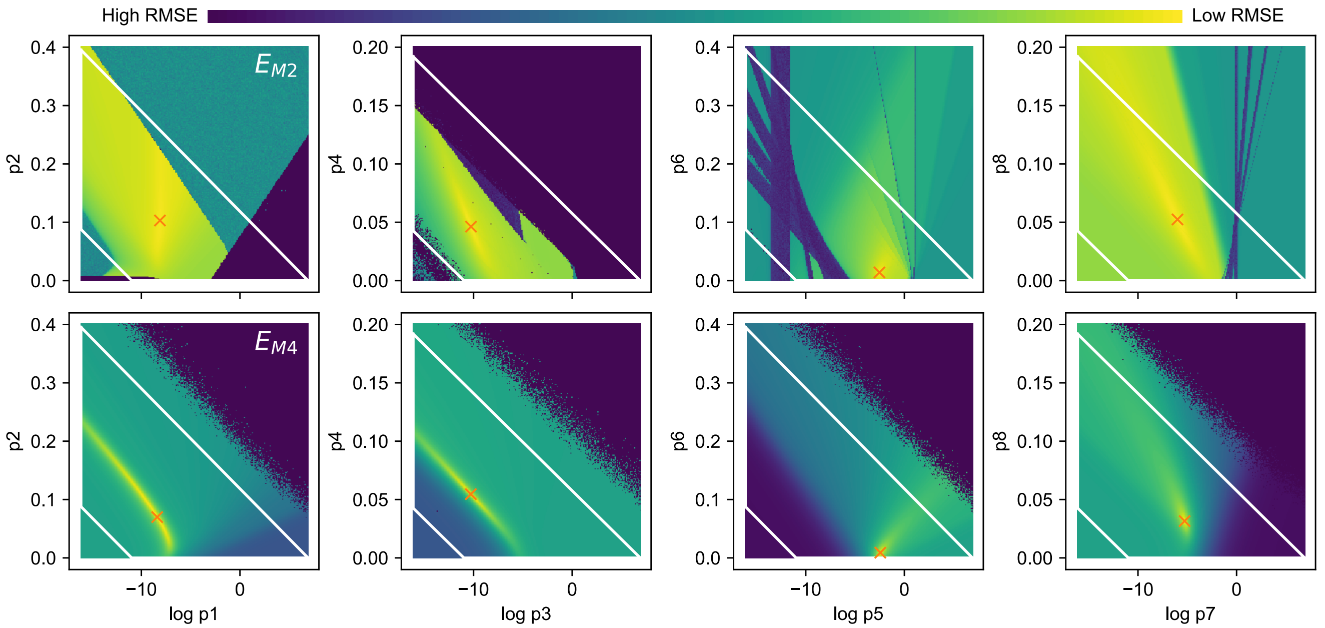

FIGURE 11 An exploration of two-dimensional slices of the objective functions. (*Top*) E_{M2} for cell #5 is shown, near the optimum found by method 2 (indicated by an *orange* "×"). Each panel shows the result of varying two parameters, with the other seven held constant. The parameters varied in the left-most panels determine the rate of activation, followed by deactivation in the second panel, inactivation in the third, and finally, the rate of recovery. The white lines indicate the boundaries used during optimization. (*Lower*) An exploration of E_{M4} for cell #5 is shown, near the optimum found by method 4. The darkest purple color in the figure represents areas where either summary curve derivation (*top panel*) or simulation (*lower panel*) failed. To see this figure in color, go online.

Another limitation stemming from the cell-specific nature of our measurements is that we cannot benefit from averaging as a noise-reduction strategy. As Fig. 2 shows, the mean summary curves over all nine cells often show qualitative behavior that is more like the expected results than our individual cell measurements. However, it has also been shown that averaging in cellular electrophysiology can lead to erroneous results (91), and there is a clear benefit of having methods that work on a single cell, thereby allowing investigations of cell-to-cell variability, heterogeneity, and measurements of cells only available in limited quantities (e.g., human tissue).

In our method 1, we did not use time-to-peak information (as suggested by (31)) and used what Willms et al. (35) refer to as the "disjoint method" rather than estimating time constants and steady states simultaneously. We did not, unfortunately, find much mention of such improved methodologies in the applied literature and so believe that our method 1 is still a good representation of a commonly followed approach. Similarly, the derivation of the summary curves might be improved in other ways, which would lead to better results for methods 1 and 2. As a counterpoint, we note that methods 3 and 4 do not require these complex intermediate analyses, are easier to implement, and do not rely on the assumption that the summary curves provide an accurate representation of the raw current data.

In deriving methods 2 and 3, subjective choices had to be made regarding weightings of the individual protocols, and changing these may alter the results. For example, similar error measures could be derived that do not normalize according to the length of each protocol or that use fixed weightings per protocol instead of the cell-specific maximal current ones we used. As it is based on a single protocol, method 4 does not require a weighting per protocol, which could be seen as an advantage because it means there are fewer subjective choices to be made during optimization. However, the lack of scalings could also be viewed as a weakness because it may be desirable (for method 4 but also 2 and 3) to scale different parts of the protocol(s) differently. Such an approach could, for example, compensate for varying current amplitudes (which otherwise cause the error function to emphasize fitting regions of the protocol that elicit stronger currents) or even weight "important" (in some sense) parts of the currents more strongly than others. For our study, we thought it advisable to avoid such "tweaking," hopefully leading to a result that is representative of standard approaches and easily applicable.

Relatedly, error measures other than RMSE could have been used for the optimization objective. Using a statistical likelihood instead of an RMSE has an advantage in that it opens the door to Bayesian analysis, which would allow prior information to be used in fitting and could be used to shed more light on the origins of observed cell-to-cell differences (92). However, the assumption of independent and identically distributed Gaussian noise may not be the most appropriate one if there are significant drifts in the system or if the high sampling rate used in recording captures single

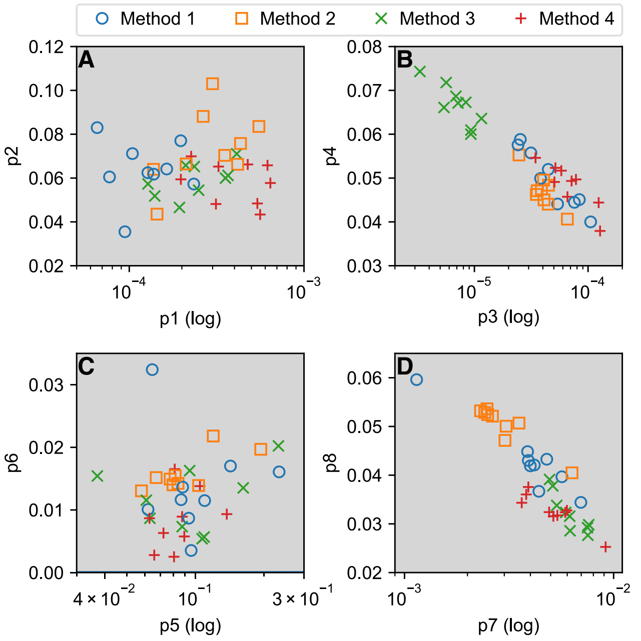

FIGURE 12 Best solutions returned by the four methods for all cells. The activation rate parameters p_1 and p_2 are shown in (*A*), deactivation in (*B*), and inactivation and recovery in (*C*) and (*D*), respectively. To see this figure in color, go online.

bursts of noise in several adjoining time points. Different "noise models," e.g., autoregressive or autoregressive moving average, might be used, but whether these are more appropriate models of voltage-clamp error is an open question that warrants further research. Because such error measures are not commonly used in the literature, our comparison here was restricted to RMSE.

Various models of hERG and I_{Kr} currents have been proposed and compared in the literature (20,93), and so far, a consensus has not been reached. All four methods in this study used the same two-gate Hodgkin-Huxley model and so share the common limitation that this model may not be the optimal choice. Studies such as Bett et al. (93) have shown that some experiments are better fitted with a five-state Markov model (94), which suggests that the method 3 and 4 fits shown in Figs. 6 and 7 could be further improved. The study by Beattie et al. (20) investigated this question (section H in its Appendix) and confirmed that the model by Wang et al. (94) provided better fits to the sine wave data. However, predictions of the AP waveform (Pr6) response (as well as the metrics derived from Pr2–5) were no more accurate than those made with the simpler four-state model. This finding suggests there is still a trade-off to make between fitting training data precisely and predictive power in new situations, and the choice of an optimal model depends on the expected "context of use" (95). However, we expect that further refinements to either models or fitting protocols could produce better fits and predictions, an area in which further work is needed.

Conversely, one might question whether the model used in this study is already more complex than the data warrant, commonly termed "overfitting": when a model is fitted to (some of the) noise in the training data, leading to an improved fit but a loss of predictive power in new situations. Using methods 3 and 4, however, we found we could accurately predict the response to the independent validation data of Pr6 (Fig. 8) and that models trained on Pr7 could predict the response to Pr2–5 (Fig. 6) and vice versa (Fig. 7), so we do not believe overfitting is an issue for this simple model.

Finally, it is worth discussing how our results may be generalized beyond Hodgkin-Huxley-style independent gating models. As noted in the introduction, method 1 requires a model for which analytic expressions for steady states and time constants are available, which means it does not generalize to arbitrary Markov models. For Markov models of intermediate complexity—but with dependent gating—we expect our results for methods 2–4 will generalize well, although more complex models with many states and transition parameters to capture may require more complex "method-4-style" protocols to be devised. We believe this is an exciting area for future work.

CONCLUSIONS

We presented and compared four methods to fit an ion current model, each based on a common class of methods used in the literature. By performing these methods on a set of CHO-cell hERG1a measurements, we found that methods based on whole-current fitting provided both the most accurate and the most reproducible results. Models fitted using a recently developed sinusoidal protocol were found to have similar predictive ability as those fitted to conventional voltage-step protocols while having a lower experimental and computational cost. Further analysis showed that using numerical optimization to fit a model to experimental steady-state and time-constant data was potentially hazardous, and we point toward increased sharing and use of raw current traces as a viable solution to this problem. Our results highlight some of the remaining challenges to make truly predictive models of ionic currents and the possibilities for novel experimental protocols to enable faster and more reliable fitting.

SUPPORTING MATERIAL

Supporting Material can be found online at https://doi.org/10.1016/j.bpj.2019.08.001.

AUTHOR CONTRIBUTIONS

M.C., D.J.G., and G.R.M. designed and performed the analysis. K.A.B. performed the experiments. K.A.B. and G.R.M. designed the experimental protocols. M.C., D.J.G., and G.R.M. wrote the manuscript. All authors approved the final version of the manuscript.

ACKNOWLEDGMENTS

This work was supported by the Biotechnology and Biological Sciences Research Council (grant number BB/P010008/1); the Engineering and Physical Sciences Research Council (grant numbers EP/I017909/1 and EP/K503769/1); and the Wellcome Trust (grant numbers 101222/Z/13/Z and 212203/Z/18/Z). M.C., G.R.M., and D.J.G. acknowledge support from a Biotechnology and Biological Sciences Research Council project grant. K.A.B. was supported by the Engineering and Physical Sciences Research Council via PhD studentship and postdoctoral support. G.R.M. acknowledges support from the Wellcome Trust & Royal Society via a Sir Henry Dale Fellowship and a Wellcome Trust Senior Research Fellowship. K.A.B. is an employee and shareholder of GlaxoSmithKline Plc.

REFERENCES

1. Hodgkin, A. L., and A. F. Huxley. 1952. A quantitative description of membrane current and its application to conduction and excitation in nerve. *J. Physiol.* 117:500–544.
2. Noble, D. 1960. Cardiac action and pacemaker potentials based on the Hodgkin-Huxley equations. *Nature.* 188:495–497.
3. Rudy, Y., and J. R. Silva. 2006. Computational biology in the study of cardiac ion channels and cell electrophysiology. *Q. Rev. Biophys.* 39:57–116.
4. Clancy, C. E., and Y. Rudy. 1999. Linking a genetic defect to its cellular phenotype in a cardiac arrhythmia. *Nature.* 400:566–569.
5. Bottino, D., R. C. Penland, …, G. S. Lett. 2006. Preclinical cardiac safety assessment of pharmaceutical compounds using an integrated systems-based computer model of the heart. *Prog. Biophys. Mol. Biol.* 90:414–443.
6. Mirams, G. R., Y. Cui, …, D. Noble. 2011. Simulation of multiple ion channel block provides improved early prediction of compounds' clinical torsadogenic risk. *Cardiovasc. Res.* 91:53–61.
7. O'Hara, T., L. Virág, …, Y. Rudy. 2011. Simulation of the undiseased human cardiac ventricular action potential: model formulation and experimental validation. *PLoS Comput. Biol.* 7:e1002061.
8. Corrias, A., and M. L. Buist. 2008. Quantitative cellular description of gastric slow wave activity. *Am. J. Physiol. Gastrointest. Liver Physiol.* 294:G989–G995.
9. Cha, C. Y., Y. Nakamura, …, A. Noma. 2011. Ionic mechanisms and Ca2+ dynamics underlying the glucose response of pancreatic β cells: a simulation study. *J. Gen. Physiol.* 138:21–37.
10. Cannon, S. C. 2007. Physiologic principles underlying ion channelopathies. *Neurotherapeutics.* 4:174–183.
11. Hille, B. 2001. Ion Channels of Excitable Membranes. Sinauer Associates, Inc, Sunderland, MA.
12. Hoefen, R., M. Reumann, …, C. M. Lopes. 2012. In silico cardiac risk assessment in patients with long QT syndrome: type 1: clinical predictability of cardiac models. *J. Am. Coll. Cardiol.* 60:2182–2191.
13. Passini, E., O. J. Britton, …, B. Rodriguez. 2017. Human in silico drug trials demonstrate higher accuracy than animal models in predicting clinical pro-arrhythmic cardiotoxicity. *Front. Physiol.* 8:668.
14. Li, Z., B. J. Ridder, …, D. G. Strauss. 2019. Assessment of an in silico mechanistic model for proarrhythmia risk prediction under the CiPA initiative. *Clin. Pharmacol. Ther.* 105:466–475.
15. Horn, R., and C. A. Vandenberg. 1984. Statistical properties of single sodium channels. *J. Gen. Physiol.* 84:505–534.
16. Hafner, D., U. Borchard, and M. Neugebauer. 1981. Parameter estimation in Hodgkin-Huxley-type equations for membrane action potentials in nerve and heart muscle. *J. Theor. Biol.* 91:321–345.
17. Milescu, L. S., T. Yamanishi, …, J. C. Smith. 2008. Real-time kinetic modeling of voltage-gated ion channels using dynamic clamp. *Biophys. J.* 95:66–87.
18. Vandenberg, C. A., and F. Bezanilla. 1991. A sodium channel gating model based on single channel, macroscopic ionic, and gating currents in the squid giant axon. *Biophys. J.* 60:1511–1533.
19. Cha, A., P. C. Ruben, …, F. Bezanilla. 1999. Voltage sensors in domains III and IV, but not I and II, are immobilized by Na+ channel fast inactivation. *Neuron.* 22:73–87.
20. Beattie, K. A., A. P. Hill, …, G. R. Mirams. 2018. Sinusoidal voltage protocols for rapid characterisation of ion channel kinetics. *J. Physiol.* 596:1813–1828.
21. Noble, D. 1962. A modification of the Hodgkin–Huxley equations applicable to Purkinje fibre action and pace-maker potentials. *J. Physiol.* 160:317–352.
22. McAllister, R. E., D. Noble, and R. W. Tsien. 1975. Reconstruction of the electrical activity of cardiac Purkinje fibres. *J. Physiol.* 251:1–59.
23. Beeler, G. W., and H. Reuter. 1977. Reconstruction of the action potential of ventricular myocardial fibres. *J. Physiol.* 268:177–210.
24. DiFrancesco, D., and D. Noble. 1985. A model of cardiac electrical activity incorporating ionic pumps and concentration changes. *Philos. Trans. R. Soc. Lond. B Biol. Sci.* 307:353–398.
25. Rasmusson, R. L., J. W. Clark, …, D. L. Campbell. 1990. A mathematical model of a bullfrog cardiac pacemaker cell. *Am. J. Physiol.* 259:H352–H369.
26. Wilders, R., H. J. Jongsma, and A. C. van Ginneken. 1991. Pacemaker activity of the rabbit sinoatrial node. A comparison of mathematical models. *Biophys. J.* 60:1202–1216.
27. Luo, C. H., and Y. Rudy. 1991. A model of the ventricular cardiac action potential. Depolarization, repolarization, and their interaction. *Circ. Res.* 68:1501–1526.
28. Courtemanche, M., R. J. Ramirez, and S. Nattel. 1998. Ionic mechanisms underlying human atrial action potential properties: insights from a mathematical model. *Am. J. Physiol.* 275:H301–H321.
29. Nygren, A., C. Fiset, …, W. R. Giles. 1998. Mathematical model of an adult human atrial cell: the role of K+ currents in repolarization. *Circ. Res.* 82:63–81.
30. Priebe, L., and D. J. Beuckelmann. 1998. Simulation study of cellular electric properties in heart failure. *Circ. Res.* 82:1206–1223.
31. Beaumont, J., F. A. Roberge, and L. J. Leon. 1993. On the interpretation of voltage-clamp data using the Hodgkin-Huxley model. *Math. Biosci.* 115:65–101.
32. Beaumont, J., F. A. Roberge, and D. R. Lemieux. 1993. Estimation of the steady-state characteristics of the Hodgkin-Huxley model from voltage-clamp data. *Math. Biosci.* 115:145–186.
33. Wang, G. J., and J. Beaumont. 2004. Parameter estimation of the Hodgkin–Huxley gating model: an inversion procedure. *SIAM J. Appl. Math.* 64:1249–1267.
34. Ebihara, L., and E. A. Johnson. 1980. Fast sodium current in cardiac muscle. A quantitative description. *Biophys. J.* 32:779–790.
35. Willms, A. R., D. J. Baro, …, J. Guckenheimer. 1999. An improved parameter estimation method for Hodgkin-Huxley models. *J. Comput. Neurosci.* 6:145–168.
36. Lee, J., B. Smaill, and N. Smith. 2006. Hodgkin-Huxley type ion channel characterization: an improved method of voltage clamp experiment parameter estimation. *J. Theor. Biol.* 242:123–134.
37. ten Tusscher, K. H., D. Noble, …, A. V. Panfilov. 2004. A model for human ventricular tissue. *Am. J. Physiol. Heart Circ. Physiol.* 286:H1573–H1589.
38. Inada, S., J. C. Hancox, …, M. R. Boyett. 2009. One-dimensional mathematical model of the atrioventricular node including atrio-nodal, nodal, and nodal-his cells. *Biophys. J.* 97:2117–2127.
39. Corrias, A., W. Giles, and B. Rodriguez. 2011. Ionic mechanisms of electrophysiological properties and repolarization abnormalities in rabbit Purkinje fibers. *Am. J. Physiol. Heart Circ. Physiol.* 300:H1806–H1813.

40. Bondarenko, V. E., G. P. Szigeti, …, R. L. Rasmusson. 2004. Computer model of action potential of mouse ventricular myocytes. *Am. J. Physiol. Heart Circ. Physiol.* 287:H1378–H1403.

41. Iyer, V., R. Mazhari, and R. L. Winslow. 2004. A computational model of the human left-ventricular epicardial myocyte. *Biophys. J.* 87:1507–1525.

42. Benson, A. P., O. V. Aslanidi, …, A. V. Holden. 2008. The canine virtual ventricular wall: a platform for dissecting pharmacological effects on propagation and arrhythmogenesis. *Prog. Biophys. Mol. Biol.* 96:187–208.

43. Aslanidi, O. V., M. R. Boyett, …, H. Zhang. 2009. Mechanisms of transition from normal to reentrant electrical activity in a model of rabbit atrial tissue: interaction of tissue heterogeneity and anisotropy. *Biophys. J.* 96:798–817.

44. Decker, K. F., J. Heijman, …, Y. Rudy. 2009. Properties and ionic mechanisms of action potential adaptation, restitution, and accommodation in canine epicardium. *Am. J. Physiol. Heart Circ. Physiol.* 296:H1017–H1026.

45. Li, L., S. A. Niederer, …, N. P. Smith. 2010. A mathematical model of the murine ventricular myocyte: a data-driven biophysically based approach applied to mice overexpressing the canine NCX isoform. *Am. J. Physiol. Heart Circ. Physiol.* 299:H1045–H1063.

46. Paci, M., J. Hyttinen, …, S. Severi. 2013. Computational models of ventricular- and atrial-like human induced pluripotent stem cell derived cardiomyocytes. *Ann. Biomed. Eng.* 41:2334–2348.

47. Colman, M. A., P. Saxena, …, A. J. Workman. 2018. Description of the human atrial action potential derived from a single, congruent data source: novel computational models for integrated experimental-numerical study of atrial arrhythmia mechanisms. *Front. Physiol*. 9:1211.

48. Moreno, J. D., T. J. Lewis, and C. E. Clancy. 2016. Parameterization for in-silico modeling of ion channel interactions with drugs. *PLoS One.* 11:e0150761.

49. Perissinotti, L. L., J. Guo, …, S. Y. Noskov. 2015. Kinetic model for NS1643 drug activation of WT and L529I variants of Kv11.1 (hERG1) potassium channel. *Biophys. J.* 108:1414–1424.

50. Teed, Z. R., and J. R. Silva. 2016. A computationally efficient algorithm for fitting ion channel parameters. *MethodsX.* 3:577–588.

51. Navarro, M. A., A. Salari, …, L. S. Milescu. 2018. Estimating kinetic mechanisms with prior knowledge II: behavioral constraints and numerical tests. *J. Gen. Physiol.* 150:339–354.

52. Balser, J. R., D. M. Roden, and P. B. Bennett. 1990. Global parameter optimization for cardiac potassium channel gating models. *Biophys. J.* 57:433–444.

53. Irvine, L. A., M. S. Jafri, and R. L. Winslow. 1999. Cardiac sodium channel Markov model with temperature dependence and recovery from inactivation. *Biophys. J.* 76:1868–1885.

54. Madden, J. L., Z. B. Miled, …, J. Schild. 2000. On parameter estimation for neuron models. *In* Proceedings IEEE International Symposium on Bio-Informatics and Biomedical Engineering. IEEE, pp. 253–262.

55. Buhry, L., F. Grassia, …, S. Saïghi. 2011. Automated parameter estimation of the Hodgkin-Huxley model using the differential evolution algorithm: application to neuromimetic analog integrated circuits. *Neural Comput.* 23:2599–2625.

56. Gurkiewicz, M., and A. Korngreen. 2007. A numerical approach to ion channel modelling using whole-cell voltage-clamp recordings and a genetic algorithm. *PLoS Comput. Biol.* 3:e169.

57. Wilhelms, M. 2013. Multiscale modeling of cardiac electrophysiology: adaptation to atrial and ventricular rhythm disorders and pharmacological treatment. PhD thesis. Karlsruher Institute für Technologie.

58. Loewe, A., M. Wilhelms, …, G. Seemann. 2016. Parameter estimation of ion current formulations requires hybrid optimization approach to be both accurate and reliable. *Front. Bioeng. Biotechnol.* 3:209.

59. Willms, A. R. 2002. NEUROFIT: software for fitting Hodgkin-Huxley models to voltage-clamp data. *J. Neurosci. Methods.* 121:139–150.

60. Milescu, L. S., G. Akk, and F. Sachs. 2005. Maximum likelihood estimation of ion channel kinetics from macroscopic currents. *Biophys. J.* 88:2494–2515.

61. Fink, M., and D. Noble. 2009. Markov models for ion channels: versatility versus identifiability and speed. *Philos. Trans. A Math. Phys. Eng. Sci.* 367:2161–2179.

62. Csercsik, D., K. M. Hangos, and G. Szederkényi. 2012. Identifiability analysis and parameter estimation of a single Hodgkin–Huxley type voltage dependent ion channel under voltage step measurement conditions. *Neurocomputing.* 77:178–188.

63. Walch, O. J., and M. C. Eisenberg. 2016. Parameter identifiability and identifiable combinations in generalized Hodgkin–Huxley models. *Neurocomputing.* 199:137–143.

64. Millonas, M. M., and D. A. Hanck. 1998. Nonequilibrium response spectroscopy of voltage-sensitive ion channel gating. *Biophys. J.* 74:210–229.

65. Kargol, A., B. Smith, and M. M. Millonas. 2002. Applications of nonequilibrium response spectroscopy to the study of channel gating. Experimental design and optimization. *J. Theor. Biol.* 218:239–258.

66. Hosein-Sooklal, A., and A. Kargol. 2002. Wavelet analysis of nonequilibrium ionic currents in human heart sodium channel (hH1a). *J. Membr. Biol.* 188:199–212.

67. Kargol, A. 2013. Wavelet-based protocols for ion channel electrophysiology. *BMC Biophys.* 6:3.

68. Clerx, M., P. Collins, and P. G. A. Volders. 2015. Applying novel identification protocols to Markov models of INa. *In* Computing in Cardiology, Volume 42. CINC, pp. 889–892.

69. Tóth, T. I., and V. Crunelli. 1995. A numerical procedure to estimate kinetic and steady-state characteristics of inactivating ionic currents. *J. Neurosci. Methods.* 63:1–12.

70. Fitzhugh, R. 1965. A kinetic model of the conductance changes in nerve membrane. *J. Cell. Comp. Physiol.* 66:111–117.

71. Tsien, R. W., and D. Noble. 1969. A transition state theory approach to the kinetics of conductance changes in excitable membranes. *J. Membr. Biol.* 1:248–273.

72. Lei, C., M. Clerx, …, G. R. Mirams. 2019. Rapid characterisation of hERG channel kinetics II: temperature dependence. *Biophys. J* Published online July 25, 2019. https://doi.org/10.1016/j.bpj.2019.07.030.

73. Hansen, N., and A. Ostermeier. 2001. Completely derandomized self-adaptation in evolution strategies. *Evol. Comput.* 9:159–195.

74. Johnstone, R. H., E. T. Y. Chang, …, G. R. Mirams. 2016. Uncertainty and variability in models of the cardiac action potential: can we build trustworthy models? *J. Mol. Cell. Cardiol.* 96:49–62.

75. Johnstone, R. H., R. Bardenet, …, G. R. Mirams. 2016. Hierarchical Bayesian inference for ion channel screening dose-response data. *Wellcome Open Res.* 1:6.

76. Chang, K. C., S. Dutta, …, Z. Li. 2017. Uncertainty quantification reveals the importance of data variability and experimental design considerations for *in silico* proarrhythmia risk assessment. *Front. Physiol.* 8:917.

77. Lei, C. L., K. Wang, …, L. Polonchuk. 2017. Tailoring mathematical models to stem-cell derived cardiomyocyte lines can improve predictions of drug-induced changes to their electrophysiology. *Front. Physiol.* 8:986.

78. Raue, A., M. Schilling, …, J. Timmer. 2013. Lessons learned from quantitative dynamical modeling in systems biology. *PLoS One.* 8:e74335.

79. Kreutz, C. 2016. New concepts for evaluating the performance of computational methods. *IFAC-PapersOnLine.* 49:63–70.

80. Villaverde, A. F., F. Fröhlich, …, J. R. Banga. 2019. Benchmarking optimization methods for parameter estimation in large kinetic models. *Bioinformatics.* 35:830–838.

81. Hass, H., C. Loos, …, C. Kreutz. 2019. Benchmark problems for dynamic modeling of intracellular processes. *Bioinformatics* Published online January 8, 2019. https://doi.org/10.1093/bioinformatics/btz020.

82. Johnstone, R. H. 2018. Uncertainty characterisation in action potential modelling for cardiac drug safety. PhD thesis. University of Oxford.

83. Clerx, M., P. Collins, …, P. G. Volders. 2016. Myokit: a simple interface to cardiac cellular electrophysiology. *Prog. Biophys. Mol. Biol.* 120:100–114.

84. Hindmarsh, A. C., P. N. Brown, …, C. S. Woodward. 2005. SUNDIALS: suite of nonlinear and differential/algebraic equation solvers. *ACM Trans. Math. Softw.* 31:363–396.

85. Jones, E., T. Oliphant, …. 2001. SciPy: Open source scientific tools for Python. http://www.scipy.org/ [Online; accessed 2017–08–17].

86. Clerx, M., M. Robinson, …, D. J. Gavaghan. 2019. Probabilistic inference on noisy time series (PINTS). *J. Open Res. Softw.* 7:23.

87. Cannon, R. C., and G. D'Alessandro. 2006. The ion channel inverse problem: neuroinformatics meets biophysics. *PLoS Comput. Biol.* 2:e91.

88. Ranjan, R., G. Khazen, …, H. Markram. 2011. Channelpedia: an integrative and interactive database for ion channels. *Front. Neuroinform.* 5:36.

89. Vandenberg, J. I., M. D. Perry, …, A. P. Hill. 2012. hERG K(+) channels: structure, function, and clinical significance. *Physiol. Rev.* 92:1393–1478.

90. Lee, W., M. J. Windley, …, A. Hill. 2019. Protocol-dependent differences in IC50 values measured in hERG assays occur in a predictable way and can be used to quantify state preference of drug binding. *Mol. Pharmacol.* 95:537–550.

91. Golowasch, J., M. S. Goldman, …, E. Marder. 2002. Failure of averaging in the construction of a conductance-based neuron model. *J. Neurophysiol.* 87:1129–1131.

92. Lei, C., M. Clerx, …, K. Wang. 2019. Rapid characterisation of hERG channel kinetics I: using an automated high-throughput system. *Biophys. J.* Published online July 25, 2019. https://doi.org/10.1016/j.bpj.2019.07.029.

93. Bett, G. C., Q. Zhou, and R. L. Rasmusson. 2011. Models of HERG gating. *Biophys. J.* 101:631–642.

94. Wang, S., S. Liu, …, R. L. Rasmusson. 1997. A quantitative analysis of the activation and inactivation kinetics of HERG expressed in Xenopus oocytes. *J. Physiol.* 502:45–60.

95. Gray, R. A., and P. Pathmanathan. 2018. Patient-specific cardiovascular computational modeling: diversity of personalization and challenges. *J. Cardiovasc. Transl. Res.* 11:80–88.

Biophysical Journal, Volume *117*

Supplemental Information

Four Ways to Fit an Ion Channel Model

Michael Clerx, Kylie A. Beattie, David J. Gavaghan, and Gary R. Mirams

CONTENTS

S1 Understanding voltage protocols **2**
S1.1 Phase plane analysis . . . 2
S1.2 Pr2: A time constant of activation . . . 4
S1.3 Pr3: The steady state of activation . . . 5
S1.4 Pr4: Time constant of inactivation . . . 6
S1.5 Pr5: Time constants, IV curve, and steady state of inactivation . . . 7
S1.5.1 A note on calculating steady-state of inactivation . . . 9
S1.6 The summary curves are not the model variables . . . 10
S1.7 Pr6: AP validation protocol . . . 11
S1.8 Pr7: Sinusoidal protocol . . . 11
S1.9 Three-dimensional phase diagrams . . . 12
S1.10Improving experimental protocols . . . 13

S2 Supplementary methods **14**
S2.1 Experimental data for all 9 cells . . . 14
S2.2 Boundaries on the parameter space . . . 14
S2.3 Cell S: synthetic data study . . . 15
S2.3.1 Synthetic data fits provide excellent predictions . . . 15
S2.3.2 Points with a low RMSE were clustered in a tight region . . . 17
S2.3.3 Fits were accurate, but had a small noise-induced bias . . . 18
S2.4 Using transformed parameter spaces . . . 19
S2.4.1 Effects of transformations on reliability . . . 21
S2.4.2 Effects of transformations on performance . . . 22
S2.4.3 Recommendations . . . 22

S3 Supplemental results **23**
S3.1 Obtained parameters . . . 23
S3.2 Validation and cross-validation figures for all cells . . . 23
S3.3 Relative RMSE tables for all cells . . . 24
S3.4 Performance . . . 25
S3.5 Cross-sections of the optimisation surfaces . . . 27
S3.6 Method 1b: Minimising E_{M1} . . . 28
S3.7 Method 2b: Minimising E_{M2} starting from Method 1 result . . . 30
S3.8 Method 3b: Minimising E_{M2} starting from Method 1 result . . . 30

This document contains supporting material for the article "Four ways to fit an ion current model". Further figures, animations, and code can be found at `https://github.com/CardiacModelling/FourWaysOfFitting`.

S1 UNDERSTANDING VOLTAGE PROTOCOLS

In this section we analyse the protocols used in this study (and in Beattie et al. (1)), using phase plane analysis. The first four protocols, Pr2–5, are adaptations of common voltage clamp protocols used to characterise I_{Kr}, while Pr7 is a novel sinusoidal protocol intended to provide the same information in a much shorter time. Pr6 is a collection of (regular and irregular) action potential wave forms to measure the behaviour of I_{Kr} under physiological and pathological conditions.[1] As in Beattie et al. (1), we used Pr6 as a *validation* protocol, while either Pr7 or the set Pr2–5 were used for model *fitting*. Note that the full set of protocols was run on every cell.

There are some similarities between the protocols. Pr2–5 are all periodic protocols, repeated several times (with each repeat shown in a different colour in the figures) with a change either in one step's duration (Pr2) or voltage (Pr3–5). All protocols in this study start with a constant holding potential of −80 mV followed by a brief step down −120 mV. Because I_{Kr} is mostly inactive at these potentials, this allows the I_{Kr}-independent leak current to be estimated and subtracted from the signal (1). The protocols end with another step down to −120 mV, which is intended to rapidly bring the channels into a closed state, thereby reducing the time needed to settle back to steady state between repeats or between experiments.

S1.1 Phase plane analysis

Using a two-dimensional model (see main manuscript) allows us to represent each possible state as a point on a *phase plane*, in which we plot activation a on the x-axis and recovery r on the y-axis. By running simulations and plotting the trajectories of a and r in the plane we can show the motivation behind different voltage-clamp protocols in terms of the types of behaviour they provoke.

Held at any given voltage V, the model will eventually converge to a steady state $a = a_\infty(V)$, $r = r_\infty(V)$ known as a *stable node*, see Figure S1.A. When V is changed abruptly, the stable node instantaneously moves to a new position, to which the states then converge with speeds dictated by $\tau_a(V)$ and $\tau_r(V)$. With typical parameters for I_{Kr}, inactivation/recovery is orders of magnitude faster than activation/deactivation so that many trajectories through the phase space will start with fast vertical movement followed by a slower horizontal drift. The model conductance at any point (a, r) in the plane is proportional to the product $a \cdot r$. Connecting points with an equal $a \cdot r$ leads to the iso-conductance lines shown in Figure S1.B, which indicate the fraction of maximal conductance g_{Kr} in different regions. Using these two graphs an intuitive idea of I_{Kr} behaviour may be developed, and we will use these phase planes to analyse different voltage protocols throughout the text. An example of a phase plane trajectory is shown in Figure S2.

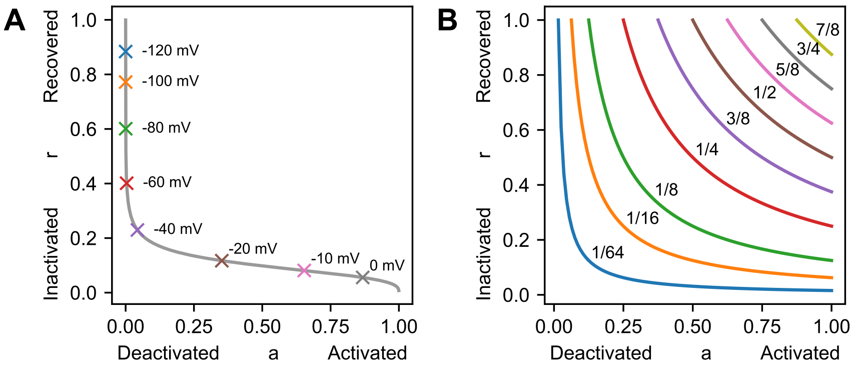

Figure S1: A guide to interpreting phase portraits. (*A*) At any voltage V, the point $(a_\infty(V), r_\infty(V))$ forms a *stable node* to which the system, if held at this V, will converge. The grey line in the figure is formed by plotting these stable nodes for a wide range of voltages, based on a simulation with the parameters for Cell #5 identified in Beattie et al. (1). (*B*) Different points in the phase plane correspond to different fractions of the maximal conductance (given by the product $a \cdot r$). When all the points for a given fraction are connected they form the iso-conductance lines shown here. Combining these two figures we can see that the channel's steady-states have low conductance while any larger currents are necessarily transient.

[1]Although we measured current through a hERG1a channel in a CHO cells, we will occasionally use the shorthand I_{Kr} to describe the current in this manuscript

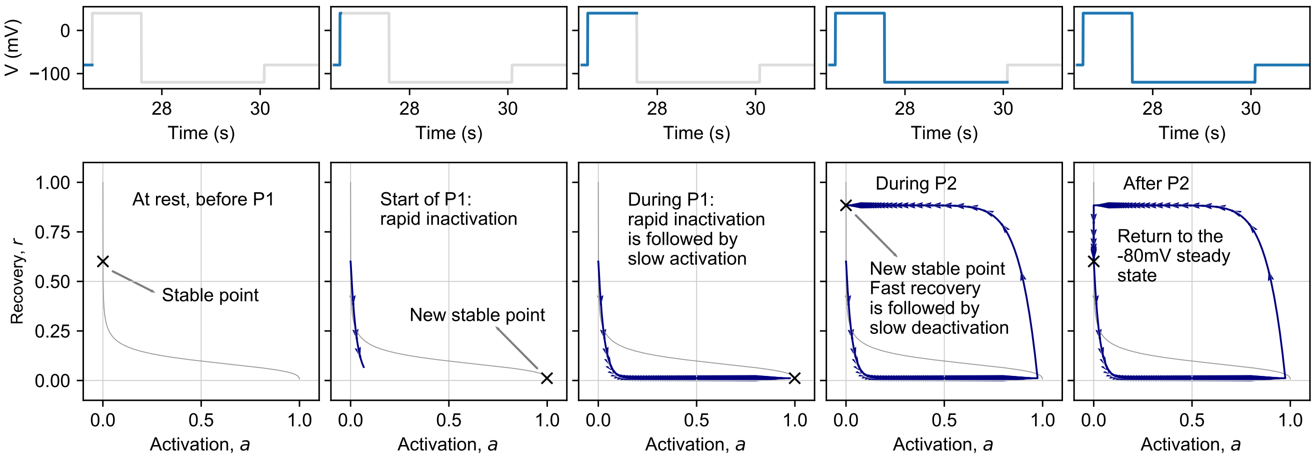

Figure S2: A simulated phase plane trajectory for the final repeat of protocol Pr2. In the first panel, the cell is being held at −80mV, and the system is in its steady state $a_\infty(V = -80), r_\infty(V = -80)$. In the next panel, a voltage step (P1) is applied, causing an instantaneous jump of the stable point towards the bottom right of the graph. The system now starts to rapidly inactivate, resulting in a downward trajectory in the phase plane. After a few milliseconds, inactivation is nearly complete, and activation begins to dominate the trajectory, resulting in the horizontal trajectory shown in the third panel. At the end of P1, the system is at (or very close to) its new stable state. Now, a new step (P2) is applied, again leading to an instantaneous jump of the stable state, followed by a vertical-then-horizontal trajectory of the system through phase space. In the final panel, the original membrane potential is restored, causing the system to revert to its original stable state.

S1.2 Pr2: A time constant of activation

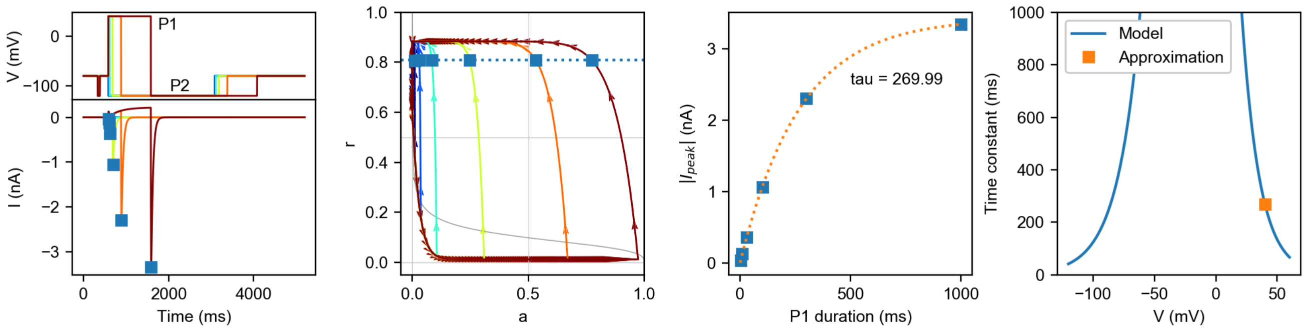

Figure S3: Simulated analysis of Pr2, approximating the time constant of activation at $V_2 = 40$mV. The protocol and current are shown in the left-most panel, with the peak currents during each repeat highlighted. The same highlighting is applied in the phase diagram, which shows that all peaks occur at almost the same level of recovery, $r \approx \tilde{r}$. Next, the peak current is plotted against the P1 duration and fit with a single exponential. This results in a single time constant, which is shown in the final panel along with the underlying model variable.

Pr2 (6 repeats of 5.2s each, 31.2s in total) is used to obtain an approximation of the time constant of activation at $V = 40$ mV. Its main feature is a variable-duration step (P1) at +40 mV. In the phase diagram this corresponds to a movement from top-left (the steady-state for −80 mV) down to the stable node for +40 mV in the lower-right part of the plane. Note that only the longest step (darkest red) actually reaches the stable node, while in the other repeats the step ends before the it is reached. During this time, the model is inactivated ($r \approx 0$) to approximately the same degree for each repeat, while the activation level varies depending on the time spent at 40 mV — it is this activation level that we wish to measure. To that end, P1 is followed by a step (P2) down to −120 mV, triggering a rapid recovery and a measurable current.

We now inspect the peak currents during P2, I_{peak}. From the phase diagram we can observe that the level of recovery at the peak is roughly the same for each repeat, so that we can approximate it by some constant (but unknown) value $\tilde{r}$, and write

$$I_{\text{peak}} \approx g_{\text{Kr}} \cdot a(t_{\text{peak}}) \cdot \tilde{r} \cdot (V_2 - E_K), \tag{S1}$$

where V_2 is the voltage during the P2 step, and $a(t_{\text{peak}})$ is some unknown activation level. We can also see that the trajectory from the end of P1 (a point near the x-axis) up to the point where peak current occurs (blue squares) is near-vertical, so that the level of activation at the peak, $a(t_{\text{peak}})$, is approximately equal to the level at the end of P1, a_1, so that we can write

$$I_{\text{peak}} \approx g_{\text{Kr}} \cdot a_1 \cdot \tilde{r} \cdot (V_2 - E_K), \tag{S2}$$

Next, we solve the differential equation for a under a fixed voltage, to find

$$a(t) = a_\infty(V) - (a_\infty(V) - a_0)e^{-t/\tau_a(V)} \tag{S3}$$

where V is the voltage during the step, t is the time since the start of the step, and a_0 is the level of activation at the start of the step. Adapting this for the activation at the end of P1, we fill in $V = V_1$ and set t equal to the step duration t_1 to find

$$a_1 = a_\infty(V_1) - (a_\infty(V_1) - a_0)e^{-t_1/\tau_a(V_1)} \tag{S4}$$

which we combine with the equation for I_{peak} to find

$$I_{\text{peak}} \approx g_{\text{Kr}} \cdot \tilde{r} \cdot (V_2 - E_K) \cdot \left(a_\infty(V_1) - (a_\infty(V_1) - a_0)e^{-t_1/\tau_a(V_1)}\right) \tag{S5}$$

$$= c_1 + c_2 e^{-t_1/\tau_a(V_1)}. \tag{S6}$$

In other words, I_{peak} should (approximately) be a function of the P1 duration t_1 and three unknowns c_1, c_2, and $\tau_a(V_1)$. As a result, we can plot I_{peak} against the P1 duration t_1, and fit a single exponential to find the time constant $\tau_a(V = V_1)$.

In a typical run of experiments, this protocol would be repeated with different P1 voltages, resulting in time constants for several voltages. As the data set from (1) does not include these, we will instead obtain further time constants of activation from Pr5. Finally, a slight variation of this protocol was used in cells 7 and 8, details of which can be found in the code published with this manuscript at `https://github.com/CardiacModelling/FourWaysOfFitting`.

S1.3 Pr3: The steady state of activation

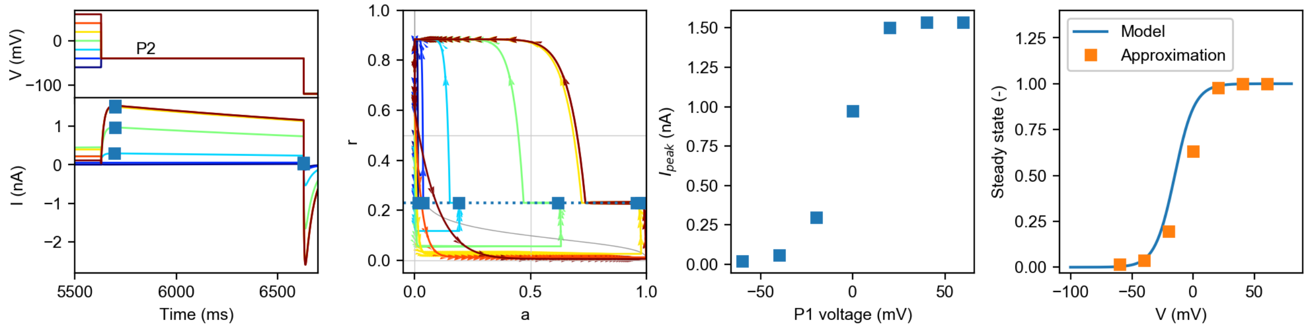

Figure S4: Simulated analysis of Pr3, approximating the steady state activation curve. The protocol and current are shown in the left-most panel, with the peak currents during each repeat highlighted. The same highlighting is applied in the phase diagram, which shows that all peaks occur at almost the same level of recovery. Next, the peak current is plotted against the P1 voltage. Because each repeat had approximately the same recovery level, $V = V_2$ for each repeat, and because the highest voltage leads to $a_\infty \approx 1$, we can normalise the peak currents by dividing through the highest obtain value to find the approximation of the steady state of activation shown in the final panel. Note the difference between the true model variable and the approximation, which is due to the incomplete approach to the steady state for voltages around 0 mV, which can be seen in the phase diagram.

Pr3 (7 repeats of approx. 8.3s each, 58s in total) is intended to characterise I_{Kr}'s steady state of activation for several voltages. Its main feature is a 5 s long variable-voltage step, P1, followed by a step P2 down to a fixed voltage, during which current is measured. In the phase plane, P1 is visible as a downwards movement from the steady state at −80 mV (top left for all repeats) to a new steady state lower on the plot. This steady state is close to $a = 1$ for high positive voltages (but notice that lower voltages have not yet quite reached the stable point after 5 s, which will be important later). Due to the large difference in the time constants of activation and recovery the P1 currents show a rapid downward movement, followed by a slower horizontal drift. At the end of P1, the system is close to the steady state of activation for the tested voltage, so that measuring the current at this point in time would provide us with clear information about the voltage-dependence of activation. Unfortunately, the level of inactivation at this point makes this current very small, so that it cannot be measured with a reasonable signal-to-noise ratio. The P2 step to a fixed voltage of −40 mV elicits a much stronger I_{Kr} current by causing rapid recovery from inactivation. This is visible in the phase plane as a rapid upward movement, which abruptly stops and turns into a slow leftward drift. Two interesting things happen near this abrupt 'corner' in the graph: (1) as this point is the furthest top-right of any point in P2, this is where the P2 peak current occurs; (2) recovery reaches its steady-state for the P2 voltage ($r_\infty(V_2) \approx 0.23$), which is the same for every test voltage repeat. Since there has been very little change in activation a when the peak current is reached, we can approximate the peak current by

$$I_{\text{peak}}(V_1) \approx g_{Kr} \cdot a_\infty(V_1) \cdot r_\infty(V_2) \cdot (V_2 - E_K) \tag{S7}$$

where V_1 and V_2 are the voltages during P1 and P2 respectively. At the highest voltage tested $V_1 = V_{\text{max}}$, we can assume that $a_\infty(V_{\text{max}}) \approx 1$, so that we can write

$$I_{\text{max}} = I_{\text{peak}}(V_1) \approx g_{Kr} \cdot r_\infty(V_2) \cdot (V_2 - E_K) \tag{S8}$$

As a result, we can divide by I_{max} to obtain

$$\frac{I_{\text{peak}}}{I_{\text{max}}} \approx a_\infty(V_1). \tag{S9}$$

This can be plotted to give the summary curve shown in Figure S4, and is commonly known as the 'activation curve'.

S1.4 Pr4: Time constant of inactivation

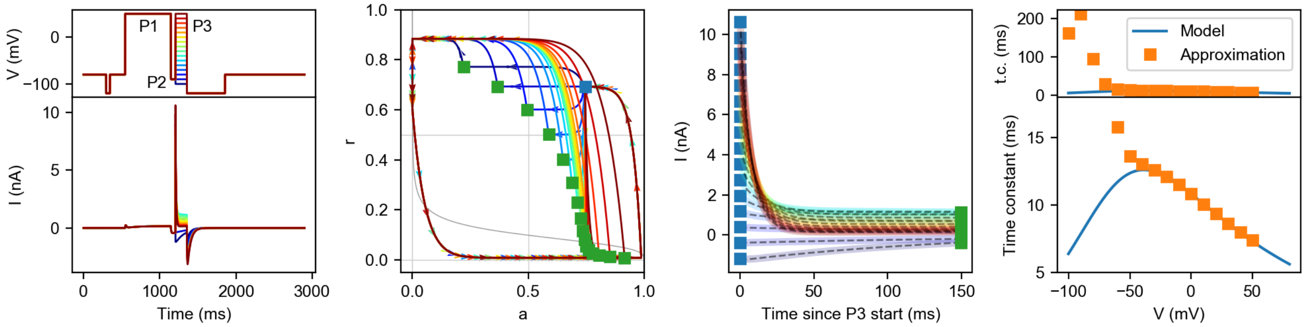

Figure S5: Simulated analysis of Pr4, approximating the time constant of inactivation. The protocol and current are shown in the left-most panel. Next, a phase diagram is shown with the start of P3 highlighted with a blue square, and the end of P3 for every repeat shown with a green square. Simulated currents during P3 are shown in the third panel, and are well-fitted by a single exponential. This yields the time constants shown in the final panel. The values obtained this way are accurate for higher voltages but contaminated by activation for lower voltages. Looking back to the phase diagram, the low voltages (blue lines) have trajectories with a strong horizontal (activation) component, while only the higher voltages (dark red lines) have trajectories determined mostly by recovery.

Pr4 (16 repeats of approx. 2.9s each, 46s in total, can be used to approximate the time constant of inactivation, and consists of a long step (P1) at +50 mV, followed by a quick step (P2) down to −90 mV and finally a variable-voltage step P3. During P1 the model quickly inactivates and then activates, which is visible in the phase plane as a movement from top-left down to the lower-right corner. P2 then causes a rapid recovery (and a large current), and only minor deactivation: an upwards movement in the phase plane that is deflected left, to a point near the phase-plane coordinates ($a = 0.75$, $r = 0.7$).

Next, the short variable-voltage step P3 is applied. Since P2 left the channels both activated and recovered, large currents can be recorded throughout P3. Due to P3's short duration and the large difference between the rates of activation and inactivation, the decays of most of these currents are characterised almost entirely by inactivation (vertical versus horizontal movements on the phase plane). As a result, we can fit exponential curves to these decays to approximate a time constant of inactivation for every tested voltage. Note however, that this assumption is increasingly invalidated for lower potentials, so that Pr4 can only be used to approximate time constants for higher voltages. The resulting time constant approximations are shown in Figure S5.

Note: Some of the most striking parts of the phase plane diagram for Pr4 correspond to the step *after* the very short P3. To trace the movements of the system through the phase plane it may be helpful to consult the 3-dimensional phase diagrams given in Figure S10 or the videos at `https://github.com/CardiacModelling/FourWaysOfFitting`.

S1.5 Pr5: Time constants, IV curve, and steady state of inactivation

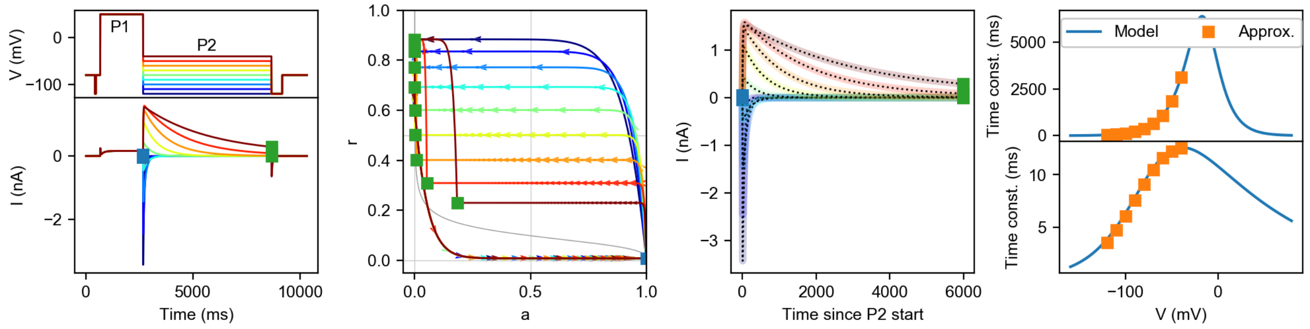

Figure S6: Simulated analysis of Pr5, approximating the time constants of activation and inactivation. The protocol and current are shown in the left-most panel, with the start and end of the P2 step indicated with blue and green squares respectively. The same highlighting is applied in the phase diagram, which shows all P2 currents start with rapid recovery, followed by slow deactivation. In the next panel, the P2 currents are plotted and shown to be well fitted by a double exponential. This results in two time constants, which are shown in the final panels along with the underlying model variables.

Pr5 (9 repeats of approx. 10.3s each, 93s in total) is used to estimate time constants of both activation and inactivation, as well as providing a graph of voltage-dependent peak currents (the 'IV curve') from which the steady states of inactivation can be approximated. The main part of Pr5 consists of a step to +50 mV (P1) followed by a variable-voltage step (P2). As before, the P1 step of Pr5 can be seen in the phase plane as a movement from top-left to a stable point in the lower-right of the plane where the channels are almost entirely activated ($a \approx 1$) and inactivated ($r \approx 0$). Next, a much lower voltage is applied during P2, causing the channels to rapidly recover and resulting in a strong current. This is shown as an upward movement in the phase plane, which then gradually turns into a horizontal movement as the system begins to deactivate. As a result, P2 is characterised by a very rapid deflection (positive or negative depending on the sign of $V_2 - E_K$) caused by recovery-from-inactivation, followed by a much more gradual decay as deactivation sets in.

We can use this two-phase character of the current by fitting one exponential to the start of the P2 current (pre-peak) to estimate the time constant of inactivation (τ_r), and fitting a second exponential to the end of the current (post-peak) to estimate the time constant of activation (τ_a) for each test voltage V_2. These estimates can be improved by fitting the sum of both exponentials directly to the currents, using the independently acquired values as initial guesses. The resulting values are plotted in Figure S6.

IV Curve and steady state of inactivation

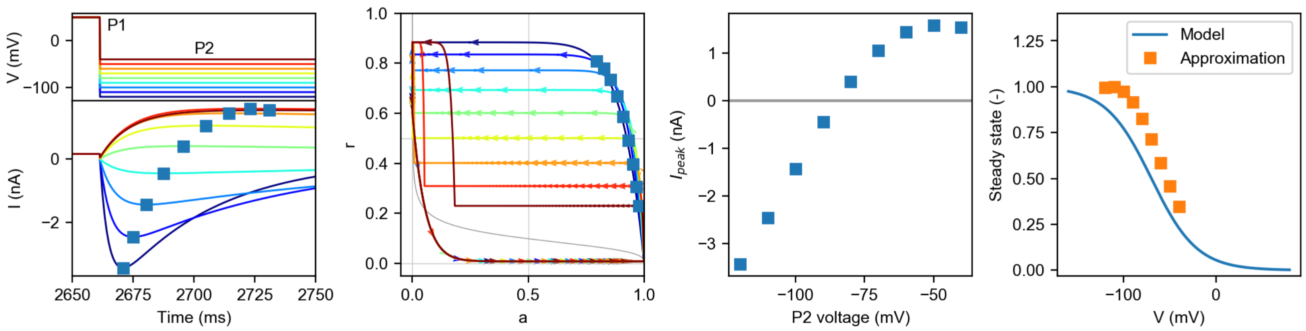

Figure S7: Simulated analysis of Pr5, approximating the steady state of inactivation. The protocol and current are shown in the left-most panel, with the peak currents during each repeat highlighted. The same highlighting is applied in the phase diagram. Next, the peak current is plotted against the P2 voltage, resulting in a (commonly referred to as 'the') IV curve. By dividing the peak current through $V_2 - E_K$ we obtain $g_{\mathrm{Kr}} \cdot a(t) \cdot r(t)$. We then use the approximations $a(t) \approx 1$ and $r(t) \approx r_\infty(V_2)$, to find $g_{\mathrm{Kr}} \cdot a(t) \cdot r(t) \approx g_{\mathrm{Kr}} \cdot r_\infty(V_2)$. Finally, we assume that the peak r measured is ≈ 1, so that $g_{\mathrm{Kr}} \approx \max\left[g_{\mathrm{Kr}} \cdot r_\infty(V_2)\right]$, and we can divide by this value to find $r_\infty(V_2)$ for every tested V_2. The resulting values are shown in the final panel, and can be seen to differ from the underlying model variable. Looking at the phase diagram, we see that the final assumption (peak $r \approx 1$) does not hold, but also that low-voltage peaks do not occur exactly at $r(t) = r_\infty$.

In the second application of Pr5, the steady-state of inactivation (r_∞) is approximated in a similar manner to Pr3. First, we extract the peak current during P2 and plot it as a function of voltage. The result is known as an IV curve, and is shown in Figure S7. Again, note that the peak current occurs when the trajectory in the phase plane changes from vertical (upwards) to horizontal (leftwards) movement, and that — especially for the higher voltages — there is relatively little deactivation at this point. As a result, the peak current can be approximated as

$$I_{\mathrm{peak}} \approx g_{\mathrm{Kr}} \cdot a_\infty(V_1) \cdot r_\infty(V_2) \cdot (V_2 - E_K) \tag{S10}$$

where $(V_2 - E_K)$ is known and $a_\infty(V_1) \approx 1$ at +50 mV. Dividing by these two quantities, we find an approximation for $g_{\mathrm{Kr}} \cdot r_\infty(V_2)$. If we further assume that for the lowest voltage $r_\infty(V_{\mathrm{min}}) \approx 1$ (again, note for later that it is actually closer to 0.9 in this parameterisation of the model) we can divide by $g_{\mathrm{Kr}} \cdot r_\infty(V_{\mathrm{min}})$ to find an approximation of $r_\infty(V_2)$ for every tested V_2. The result is shown in Figure S7

S1.5.1 A note on calculating steady-state of inactivation

Calculating steady states of activation and inactivation requires a division by $(V - E_K)$, creating a singular point at $V = E_K$ where conductance cannot be calculated, but more importantly a region around the point $V = E_K$ where any small error is amplified. Looking at Figure S7, we can see that many of the most rapidly changing (and therefore most informative) parts of the inactivation curve occur in this region.

In Figure S8.A, we have plotted the peak currents during the P2 step of Pr5 for all cells. A clear and regular trend can be seen for each cells, and all cells show qualitatively similar behaviour. In the next panel (Figure S8.B) we show the multiplication factor $(V - E_K)^{-1}$ (blue line) that is applied to the panel A data to obtain the steady-state curve (using $E_K = -88.4$mV). To illustrate what will happen if the term $V - E_K$ is imperfectly known we also plot the 10-th and 90-th percentile of the distribution $1/\mathcal{N}(V - E_K, \sigma)$, where $\mathcal{N}$ is a normal distribution and a (somewhat arbitrary) estimate $\sigma = 2$mV. This type of error could easily arise if the calculated E_K differs from the true reversal potential, or if the true transmembrane voltage differs from the command potential.

The summary curves for all cells in Figure S8.C show that this is not just a hypothetical concern, with most cells showing a dramatic deviation at -90mV (and even a change of sign for cells 5 and 6). As a result, we had to omit the data from $V = -90$mV from the summary curves for the steady state of inactivation. In Figure S8.D we plot the same data with a rescaled y-axis, and omit the -90mV points, but now it becomes clear that the wide region of error predicted by panel B is also borne out in practice, as several of the point at -100mV and -80mV also show a strong deviation from the expected sigmoid voltage-dependence.

In the summary curves used in this work, we omitted the -90mV point for both the steady state of inactivation and the time constant of inactivation (where nearness to -90mV caused problems when fitting an exponential curve). Finally, we point out that these issues (caused in part by experimental noise) can be somewhat reduced by averaging the values for all cells (a common noise-reduction technique): the mean value shown is not perfect, but displays a clearer sigmoid form than the data for the individual cells. This means that this issue, although present in all studies that use a similar summary curve, becomes more apparent when aiming for cell-specific results (see also (2)).[2]

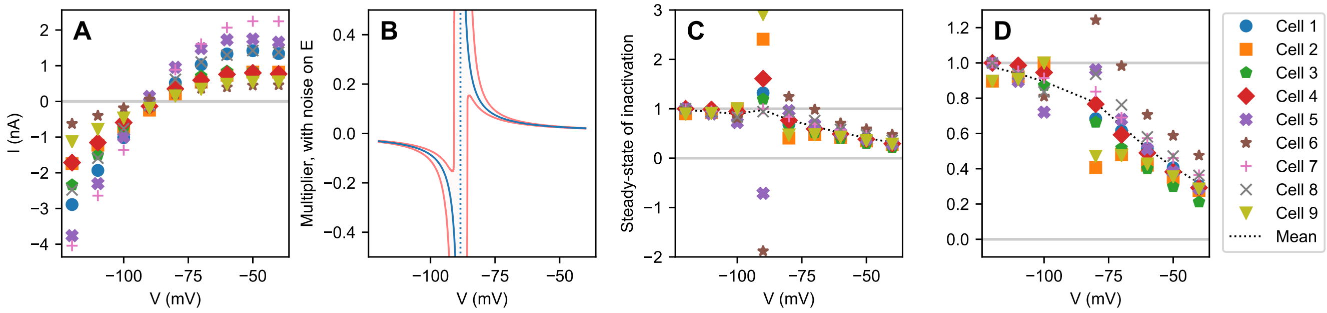

Figure S8: Approximating the steady state of inactivation. (*A*) The calculation of the steady state of inactivation starts from the IV curve data from Pr5. This data is smooth, and has the same qualitative nature for every cell. (*B*) Next, the data is multiplied by a term $1/(V - E_K)$. As shown by the blue line, this term has a large magnitude near $V = E_K$, resulting in a strong amplification of measurement error. The effect of small errors can also be seen by the red lines, which indicate the 10-th and 90-th percentile of the distribution $1/\mathcal{N}(V - E_K, 2\text{mV})$. (*C*) As expected, a major disruption is visible in the experimental data, especially near $V = -90\text{mV} \approx E_K$. (*D*) Omitting the data points for $V = -90$mV removes the largest errors, but strong effects can still be seen for -100, -80, and -70mV.

[2]It may be possible to deal with this issue by introducing a suitable noise model, and performing a weighted fit that assigns lower importance to the affected points. However, as we did not see this approach in our literature review we did not include such a method in this work.

S1.6 The summary curves are not the model variables

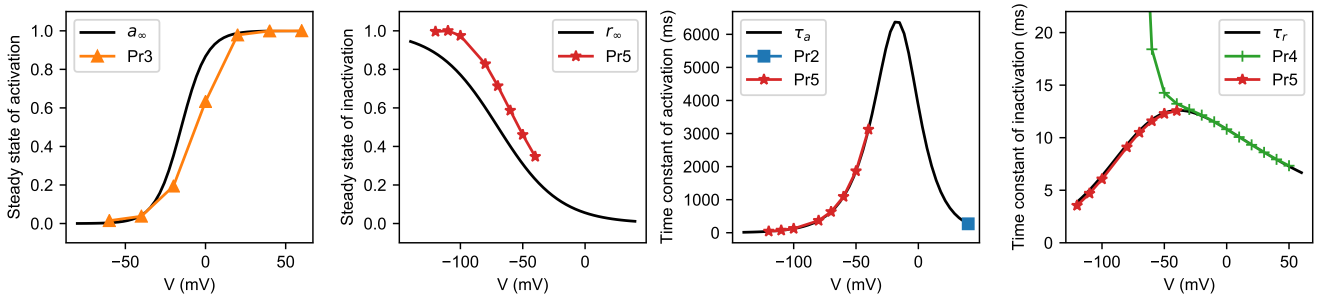

Figure S9: (*Black lines*) The model variables a_∞, r_∞, τ_a, and τ_r, all drawn using the parameters for Cell #5 given in Beattie et al. (1). (*Coloured lines*) Simulated summary curves, obtained by using the same model parameters, running simulations for Pr2–5 and performing exactly the same analysis as on the experimental data. The protocol from which each data point originates is indicated in the legend.

In explaining the rationale behind Pr2–5, we have relied heavily on the idea that they are designed to approximate the model variables a_∞, r_∞, τ_a, and τ_r. Historically, this certainly seems to how these protocols originated (see e.g. Hodgkin and Huxley (3)). However, as theories and models of I_{Kr} (and other currents) have grown in complexity the strong connection of protocols like Pr2–5 to their modelling origins has increasingly been lost. The analysis methods have, however, remained important as *biomarkers* in their own right, and many physiologists (aware of the shortcomings and pitfalls we discuss below) have chosen to interpret them as such. However, even if we take this view, the best *interpretation* we have of what these biomarkers signify is still that they resemble the variables of a two-state Hodgkin-Huxley model fit to the data. In addition, Method 1 relies on the assumption that these procedures accurately approximate the model variables, so that it's worthwhile pointing out some of the issues in their calculation below.

Steady-state of activation: The central idea of Pr3 was to reach the steady state of activation (i.e. reach the stable point for the P1 voltage) and quickly measure a current during P2. Inspecting the phase diagram for Pr3 we can see that this objective is not met for the voltages around 0mV (e.g. the blue and green lines). This leads to an underestimation of the activation at these voltages, which causes the rightward shift of the estimated steady state of activation observable in Figure S9. Less clear from this graph, is that the voltage-dependency of the effect will have caused a change in the *slope* of the estimated steady state curve. Note that using a longer variable voltage step would have brought the system closer to the stable point and reduced the apparent shift, while a shorter step would have caused an even stronger rightward shift. This time-dependency has been recognised by e.g. Vandenberg et al. (4), who warn only to compare data from protocols with an equal P1 duration, which they term 'isochronal activation data'. Similar issues have been recognised for other currents. For example, in a 1992 publication on I_{Na}, Sakakibara et al. (5) consistently avoid the term 'steady state of activation' in favour of 'normalized conductance-voltage relation', which much more cautiously describes what the 'activation' protocol has actually measured.

Steady-state of inactivation: The method to obtain a steady-state of inactivation from Pr5 relied on the assumptions that (1) the peak currents measured were not strongly affected by deactivation and (2) that the lowest voltage tested induced an inactivation level $r_\infty(V_{\mathrm{min}}) \approx 1$. As can readily be seen from the phase diagram, the first assumption is violated increasingly at lower P2 potentials. This leads to both an underestimation of the steady state of inactivation (visible as a rightward shift) and a change in the apparent slope of the inactivation curve (as lower voltages are affected more than higher ones). Looking back at the phase diagram, it is clear that the second assumption is violated too, and as a result the normalisation of the estimated curve will be off, again leading to changes in both midpoint and slope of inactivation. This second problem could be perhaps addressed by adding even lower potentials to the protocol, but notice that this would exacerbate problem 1. Staying with Pr5, it seems from the phase diagram that for lower voltages the separation between the recovering and deactivating parts of the P2 current becomes increasingly less clear. However, the strategy of fitting both at once appears to have paid off, as the Pr5 time constants in both right-hand panels of Figure S9 overlap well with the model variables.

Time constants: Finally, as already noted Pr4 fails to estimate good time constants for lower potentials (see the right-most panel in Figure S9, but this limitation can be overcome by using the Pr5 derived points instead. However, the curves from both protocols don't quite line up, indicating further issues with one or both analyses.

Previous work: Please note that the above demonstrations are not novel, but reaffirmations of (much) earlier work by e.g. Beaumont et al. (6, 1993), Willms et al. (7, 1999), and Lee et al. (8, 2006).

S1.7 Pr6: AP validation protocol

An action potential voltage clamp protocol (Pr6) is used in this study and in Beattie et al. (1) as a *validation protocol*: instead of fitting to data from these measurements we use it to evaluate predictions from models fit to the other data sets. Unlike Protocols 2–5, Pr6 does not contain any repeats. The bulk of Pr6 is a sequence of realistic action potentials, so that validation happens against physiologically relevant situations including pathological after-depolarisations. Looking at the phase diagram in the main paper (which has been coloured through time to match Pr6's voltage and current traces) we can see a secondary effect: the short time between the APs causes a build-up of activation, so that the early parts of Pr6 are a proxy for I_{Kr} during low heart rates, while the latter parts elicit I_{Kr} behaviour during periods of prolonged higher rates.

S1.8 Pr7: Sinusoidal protocol

Pr7 (single run of 8s) is a novel sinusoidal protocol introduced in Beattie et al. (1). Like Pr6, it does not contain any repeats but instead consists of a single eight second sweep. The protocol starts with a step to +40 mV followed by a step down to −120 mV, eliciting high conductance and a strong negative current. In the phase diagram shown in the main paper, this corresponds to the blue trajectory from lower-right to top-left. Note how the blue line goes close to the top-right corner of the plane (high a and r). This appears to be crucial in the protocol design for estimating the conductance parameter $p_9 = g_{\mathrm{Kr}}$ accurately: at the point $(1, 1)$ in the phase plane the current would be given by $I_{\mathrm{Kr}} = g_{\mathrm{Kr}}(V - E_K)$ allowing g_{Kr} to be estimated directly. By including a step that approaches this point, we gain a lot of information about g_{Kr}.

The remainder of the protocol consists of three sine waves of varying frequencies added together. In the phase diagram, this induces rapid near-vertical movements from which we can infer the properties of inactivation, but also slower horizontal movements that tell us about activation, across the full physiological range of voltages. Importantly, many changes in the trajectory happen far from the x and y-axis – in other words – many of the dynamical changes induced by Pr7 occur while strong I_{Kr} is being generated and the current is experimentally observable.

S1.9 Three-dimensional phase diagrams

The voltage-dependence of the steady state can be made more clear by plotting the phase diagrams in three dimensions, with voltage on the third axis.

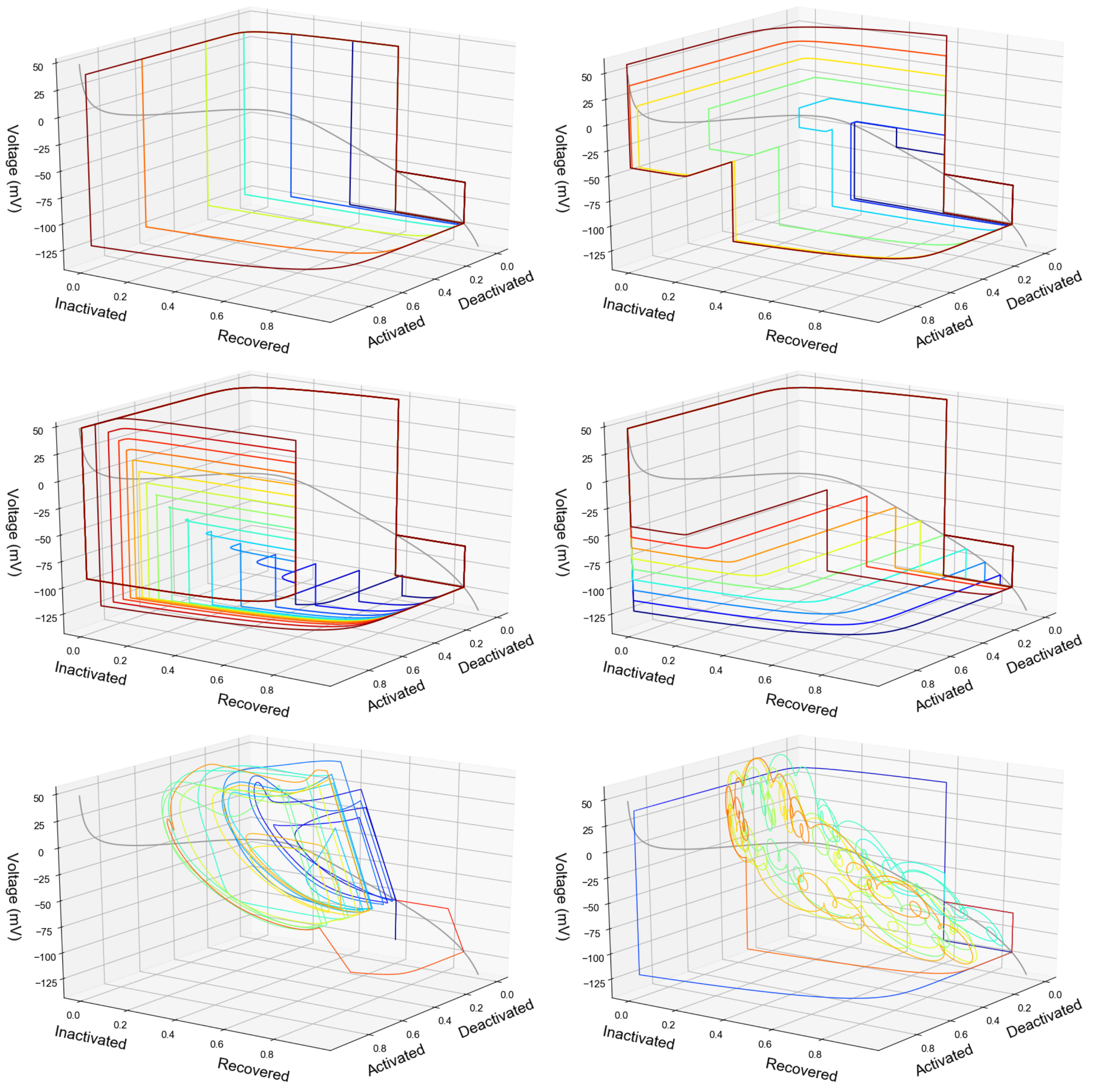

Figure S10: Three-dimensional phase diagrams for Pr2–7.

S1.10 Improving experimental protocols

Hints for improved protocol design can be found in the phase plane diagrams shown above. Inspecting the phase plane diagrams in Figure S10, we can see that large parts of Pr2–5 are concerned with setting up the system for a measurement, e.g. waiting to get into a certain steady-state, and subsequently with restoring the original state again. These steps were necessary for manual analysis, but have less use for Method 3. Following the trajectory of the system for these parts of the protocols, we see it is mostly near the x-axis and y-axis. From Figure S1.B we can see that these are areas where the system has low conductance, leading to very small currents and a poor signal-to-noise ratio. For Method 3, these parts provide information about the noise in the signal, but not about the current's dynamics. By contrast, Pr7 spends a large proportion of its time away from axes, in the 'measurable area'.

Looking at the phase planes further, it is tempting to think that exploring the full plane is a desirable property of the protocol. However, our goal in parameterising a model is not to visit every state, but to observe the kinetic parameters (p_1 to p_8) in action for as many voltages as possible. In other words, we want to observe the current while the system makes each of its four transitions (activation, deactivation, inactivation, and recovery), for all physiologically relevant voltages. (Note that if we had full confidence in our model, a few voltages per parameter would suffice, as the equations constrict the system once a few points are known.) The three-dimensional phase planes shown in Figure S1 demonstrate how the sine wave protocol comes close to realising this ideal. It visits a wide range of physiological voltages, and makes seemingly arbitrary transitions throughout the voltage range. Note however, that it still has relatively low conductance throughout, so that adapting the protocol to start with greater levels of activation may be advantageous. To improve the protocol's performance on predicting deactivation, it may also be useful to add a lower frequency sine wave, causing greater activation while the existing higher frequencies stop the system from inactivating and reducing the amount of observable current.

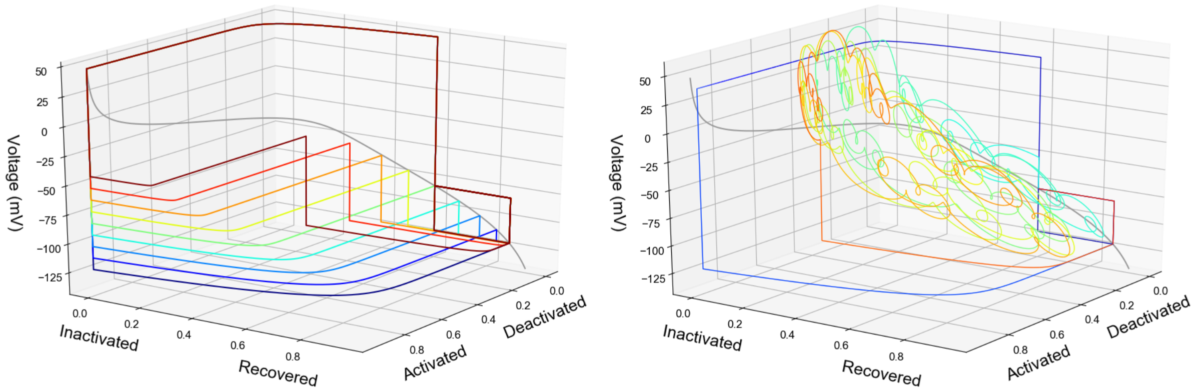

Figure S11: Three-dimensional phase diagrams for Pr5 and Pr7. The relative sparsity of the Pr5 can clearly be seen in this plot, sticking mostly to the walls (where no current is observable) and making only brief controlled forays into observable space. This careful setting up of the right conditions before measuring anything is highly advantageous for traditional analysis, but is not the most efficient strategy for whole-current fitting methods. By contrast the chaotic nature of Pr7 looks very difficult to interpret, but induces all four transitions (activation, deactivation, inactivation, and recovery) at several voltages, all the while producing measurable current.

S2 SUPPLEMENTARY METHODS

S2.1 Experimental data for all 9 cells

Figures showing the data for all 9 cells can be found at https://github.com/CardiacModelling/FourWaysOfFitting.

S2.2 Boundaries on the parameter space

Boundaries were defined on the parameter space, based on physiological constraints. This is similar to the concept of a *prior* in Bayesian inference, as it encodes our prior knowledge about the parameter values. As in Beattie et al. (1), we constrained (i) the maximum conductance g_{Kr} (parameter p_9); (ii) the values of the kinetic parameters p_1 to p_8; and (iii) the reaction rates k_1 to k_4.

Lower and upper bounds for the maximum conductance in each cell were estimated by Beattie et al. (1), and are shown in Table S1. The lower conductance for each cell was estimated in Beattie et al. (1) by assuming that the current was fully conducting ($a = r = 1$) at some point after the initial +40 mV step of the sine wave protocol (see Beattie et al. (1) for details). An upper bound was then derived by assuming that $a \cdot r > 0.1$ at this point.

Table S1: Cell-specific limits on the conductance parameter

Cell #	g_{lower} (mS)	g_{upper} (mS)
1	0.0478	0.478
2	0.0255	0.255
3	0.0417	0.417
4	0.0305	0.305
5	0.0612	0.612
6	0.0170	0.170
7	0.0886	0.886
8	0.0434	0.434
9	0.0203	0.203

Wide bounds for the kinetic parameters were set based on expected physiological ranges of the resulting reaction rates, as well as their expected voltage sensitivity (1):

$$10^{-7}\,\text{ms}^{-1} \leqslant p_i \leqslant 10^{3}\,\text{ms}^{-1}, \quad i \in 1, 3, 5, 7, \tag{S11}$$

$$10^{-7}\,\text{mV}^{-1} \leqslant p_j \leqslant 0.4\,\text{mV}^{-1}, \quad j \in 2, 4, 6, 8. \tag{S12}$$

Additionally, we set lower and upper bounds for the *maximum* transition rates, representing timescales of a minute to a microsecond, using

$$1.67 \cdot 10^{-5}\,\text{ms}^{-1} \leqslant k_i(V = +60) \leqslant 1000\,\text{ms}^{-1}, \quad i \in 1, 3, \tag{S13}$$

$$1.67 \cdot 10^{-5}\,\text{ms}^{-1} \leqslant k_j(V = -120) \leqslant 1000\,\text{ms}^{-1}, \quad j \in 2, 4. \tag{S14}$$

Here, the values for the lower and upper bounds, again taken from Beattie et al. (1), are chosen to yield (very wide) limits on what can be considered a physiologically realistic maximum reaction rate during the sine wave protocol, which is restricted to a voltage range from −120 mV to +60 mV. The additional rate constraints in Eq. (S13–S14) are functions of two parameters, so they effectively specify 2-dimensional constraints on the parameter pairs (p_1, p_2), (p_3, p_4), (p_5, p_6), and (p_7, p_8), which are shown in Figure S16. When running optimisations, parameter sets that violated any of the boundary conditions were assigned an error of ∞.

S2.3 Cell S: synthetic data study

To test the reliability of the different fitting methods in a setting where the ground truth is known, we conducted a synthetic data study. Synthetic data sets were created by simulating Pr2–5 and Pr7 using the 'Cell #5' parameters given by Beattie et al. (1). Independent identically-distributed Gaussian noise samples were added to these signals, using mean of 0 and a standard deviation of 0.025, which was comparable with the noisiest signal in our data set (for which we estimated a standard deviation of approximately 0.0247). We refer to the resulting traces as 'Cell S' data.

S2.3.1 Synthetic data fits provide excellent predictions

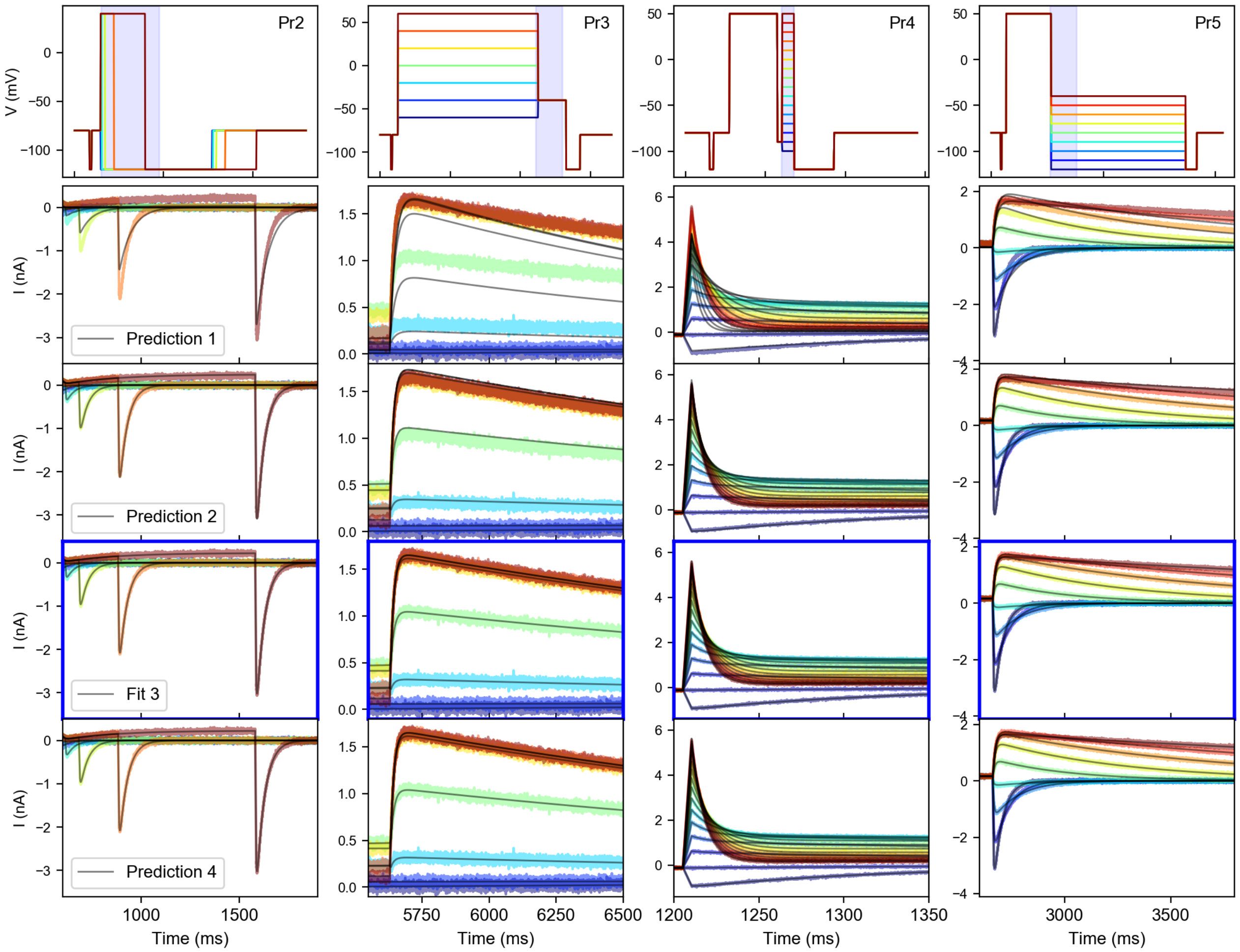

Figure S12: Method 3 goodness-of-fit and cross validation on the synthetic Cell S. As in the main manuscript, the top row shows the voltage protocols Pr2–5, with different colours used for each sweep. The rows below show predictions of (1, 2, and 4) and a fit to (3) the synthetic data from Cell S.

Figure S12 shows a fit to Pr2–5 using Method 3, and the predictions of the Method 3 data using methods 1, 2, and 4. As expected, the row labelled 'Fit 3' now shows an excellent fit, indicating that the fitting methods are able to find a good solution to the optimisation problem — although this does not yet prove this solution is unique. The prediction made by a model fit using Method 4 is also shown to provide a good fit: this shows that models made using this method have a good predictive abilities (and is an indicator that we are not overfitting but have captured an underlying trend in the data). Interestingly, the prediction from the Method 2 model is good, but not as good as the prediction from Method 4. Finally, as expected from the analysis of the protocols in the first section of this supplement, the issues with Method 1 show up in the synthetic data study too, leading to poor predictions.

These findings are confirmed in the validation results shown in Figure S13.

A quantitative view of this data is presented in Figure S14. For methods 2–4 we now see that methods 3 and 4 perform very

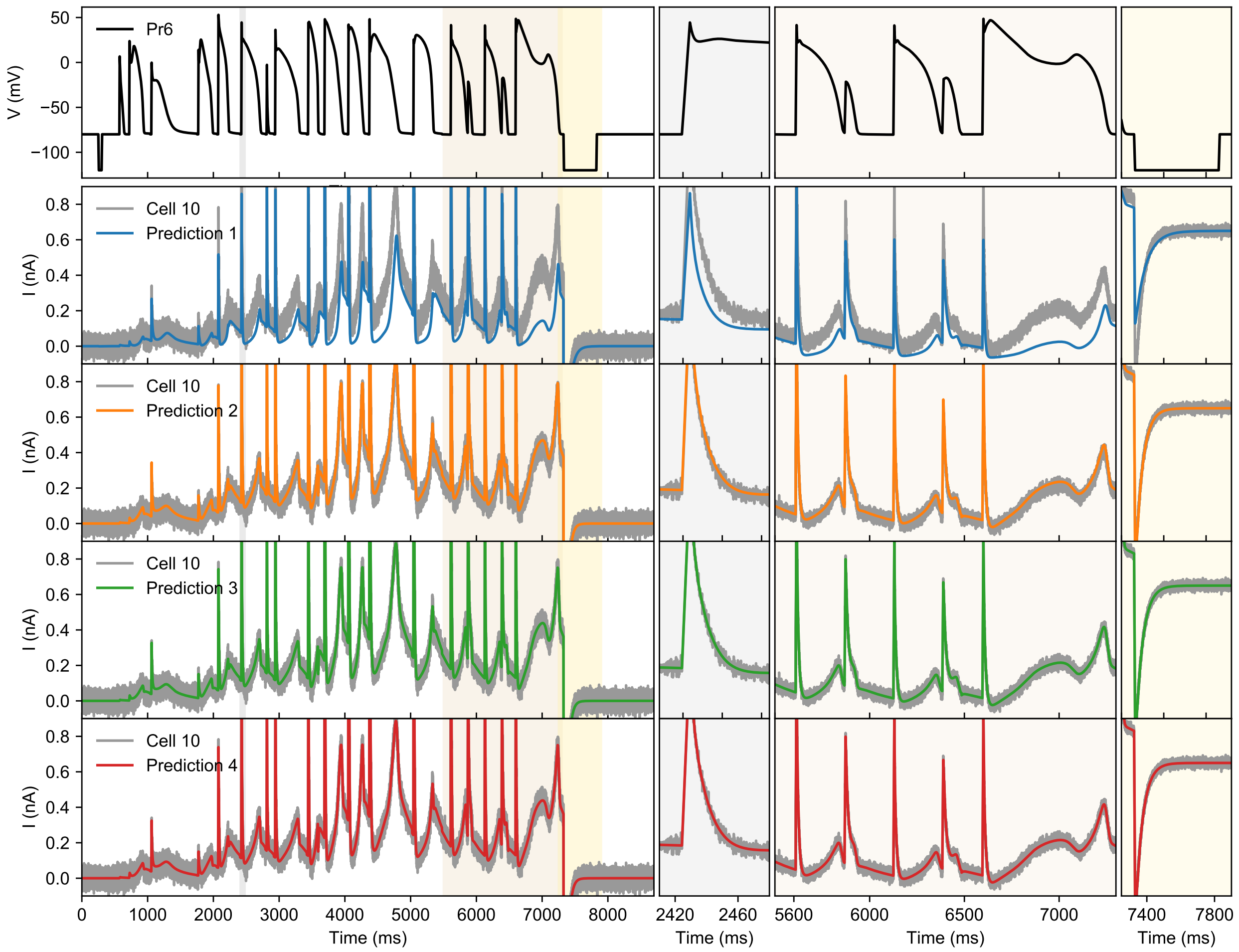

Figure S13: Validation on the AP-waveform signal (Pr6) in the synthetic Cell S

well when predicting each others' training data, as well as the AP validation data. Method 2 fits well to its own data set, but the resulting model has less predictive power than the models made with methods 3 and 4.

As was visible in the previous figures, Method 1 performs poorly. Notably, the result for 'Method 1' is not the best in the row for 'Method 1 RMSE'. This is possible because, unlike methods 2–4, the evaluation method for Method 1 (an RMSE measure defined in equation 17 of the main manuscript) is not minimised directly by Method 1.

Cell 10	Method 1	Method 2	Method 3	Method 4
AP validation	5.77	1.17	1.00	1.00
Method 1 RMSE	1.60	1.00	1.00	1.00
Cross-validation M2	35.59	1.00	1.57	1.72
Cross-validation M3	3.59	1.17	1.00	1.00
Cross-validation M4	4.67	1.12	1.00	1.00

Figure S14: Validation and cross-validation results for the synthetic Cell S. Note that Method 1 here is being applied to the simulated data from Cell S, rather than the idealised summary curves underlying the model, mimicking the experimental situation.

S2.3.2 Points with a low RMSE were clustered in a tight region

Identifiability problems can occur if, instead of having one best solution, multiple disparate points in parameter space result in similar 'best' fits. To investigate if this was the case in our data, we looked at the parameters and RMSEs returned from each repeat of methods 2–4 (80 repeats were run for Method 2, versus 50 repeats for Method 3 and Method 4). From these results, we selected the subset with an RMSE within 1% of the best RMSE returned for each cell and each method. Next, we defined the variation in an obtained parameter value p_i as

$$v_i = \frac{|p_{ij} - p_{i,1}|}{p_{i,1}} \cdot 100\% \tag{S15}$$

where p_{ij} is the p_i value obtained in the j-th repeat, and the repeats are ordered from best to worst (so that $p_{i,1}$ are the parameters leading to the highest score). We then defined the maximum variation in each subset as:

$$v_{\max} = \max_i \max_j \frac{|p_{ij} - p_{i,1}|}{p_{i,1}} \cdot 100\% \tag{S16}$$

The results per method per cell are shown in Table S2. For the synthetic data of Cell S, all results with a low RMSE are clustered in a small region of the parameter space. For methods 3 and 4, this also holds true for the real data. For Method 2 however, the best results from some cells show considerable variation. To further investigate this, we counted how many of the 'best RMSE' results were within 1% parameter variation of the best result. The results, displayed in Table S3, show that the large spread observed for some cells' Method 2 results is typically due to a small number of outliers. For Cell #9 however, only 2 in 80 repeats gave a good result, and neither result was close to the other. These results indicate there may be some identifiability problems for Method 2.

Table S2: Maximum variation $v_{\max}$ in the parameters with a low RMSE per method, per cell

Method	Cell S	Cell 1	Cell 2	Cell 3	Cell 4	Cell 5	Cell 6	Cell 7	Cell 8	Cell 9
2	6e-5	0.01	11.520	14.184	5.227	1.262	0.91	16.997	10.160	3.251
3	1e-6	3e-6	5e-6	3e-6	4e-6	5e-6	5e-6	4e-6	4e-6	8e-6
4	0.04	0.19	0.27	0.11	0.09	0.10	0.07	0.14	0.13	0.13

Table S3: The number of results with an RMSE within 1% of the best result (right), and the number that were also with 1% maximum variation of the best result's parameters (left). Note that 80 repeats were run for Method 2, but only 50 repeats for Method 3 and Method 4.

Method	Cell S	Cell 1	Cell 2	Cell 3	Cell 4	Cell 5	Cell 6	Cell 7	Cell 8	Cell 9
2	8 / 8	2 / 2	9 / 10	5 / 7	8 / 10	5 / 9	3 / 3	5 / 7	11 / 12	1 / 2
3	40 / 40	42 / 42	39 / 39	34 / 34	26 / 26	36 / 36	34 / 34	29 / 29	40 / 40	25 / 25
4	41 / 41	47 / 47	39 / 39	30 / 30	36 / 36	40 / 40	40 / 40	43 / 43	42 / 42	37 / 37

S2.3.3 Fits were accurate, but had a small noise-induced bias

In the synthetic study, the ground truth is known, and so we can also assess the accuracy of the obtained parameters. However, as we purposely created a noisy data set, we should expect this noise to have created a *bias* in our data (similar to the standard error of the mean, when estimating the mean from a finite sample). Furthermore, this bias should diminish with the number of data points used to calculate the error measures, so that method 2 should have the largest bias (42 data points), followed by method 4 (79,600 points after capacitance filtering), followed by method 3 (2,271,442 points after capacitance filtering). This prediction is borne out in Figure S15, which shows a lower RMSE was found by the optimiser for methods 2, 3, and 4, with the difference in RMSE being greatest in method 2 and smallest in method 3.

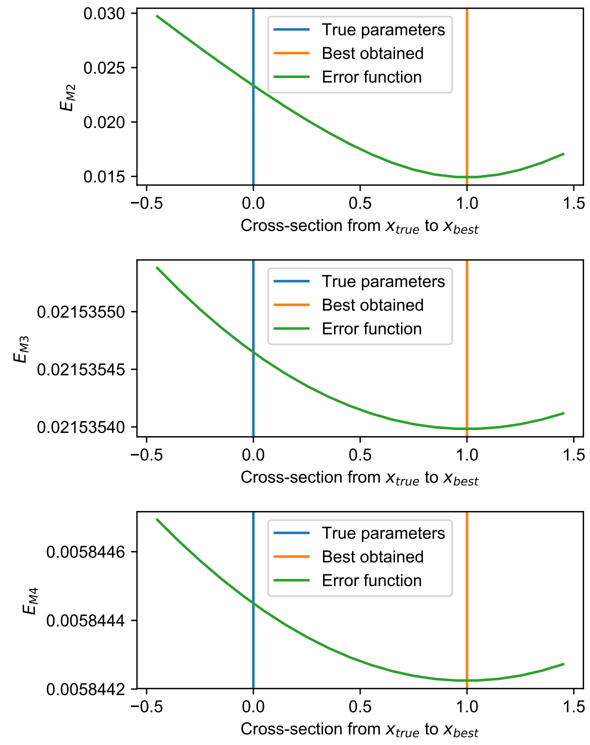

Figure S15: Noise in the synthetic data causes a shift in the point with the lowest RMSE, so that the best parameter set found by the optimiser deviates slightly from the ground truth. This effect diminishes when the number of data points increases, so that it is greatest in E_{M2} and smallest in E_{M3} (note the different y-axis scales).

S2.4 Using transformed parameter spaces

The transition rates in our model are of the form $k = a \exp(\pm bV)$, with bounds on k, a and b to keep behaviour in physiologically relevant timescales as explained in Section S2.2.

When running optimisations for the main manuscript, we used a log-transformation on all 'a parameters', that is p_1, p_3, p_5, and p_7, in that both:

1. Initial guesses for each optimisation were chosen by sampling uniformly across this transformed space; and
2. The optimiser itself worked with parameters in the transformed space (de-transforming before every simulation).

In this section of the Supporting Materials, we outline how we made this choice by using the synthetic data of Cell S.

The result of sampling uniformly in the different spaces is visualised in Figure S16, which shows the boundaries described in Section S2.2, drawn with two linear axes (top), a logarithmic x-axis (middle), or two logarithmic axes (bottom). We have denoted these transforms as:

- *n-space* — no transform on either a or b parameters.
- *a-space* — log transform on a parameters ($p_{1,3,5,7}$), no transform on b parameters.
- *f-space* — fully log transformed, for both a and b parameters (as well as the conductance parameter p_9).

Initial guesses were sampled uniformly within the boundaries in each space, as shown in Figure S16.

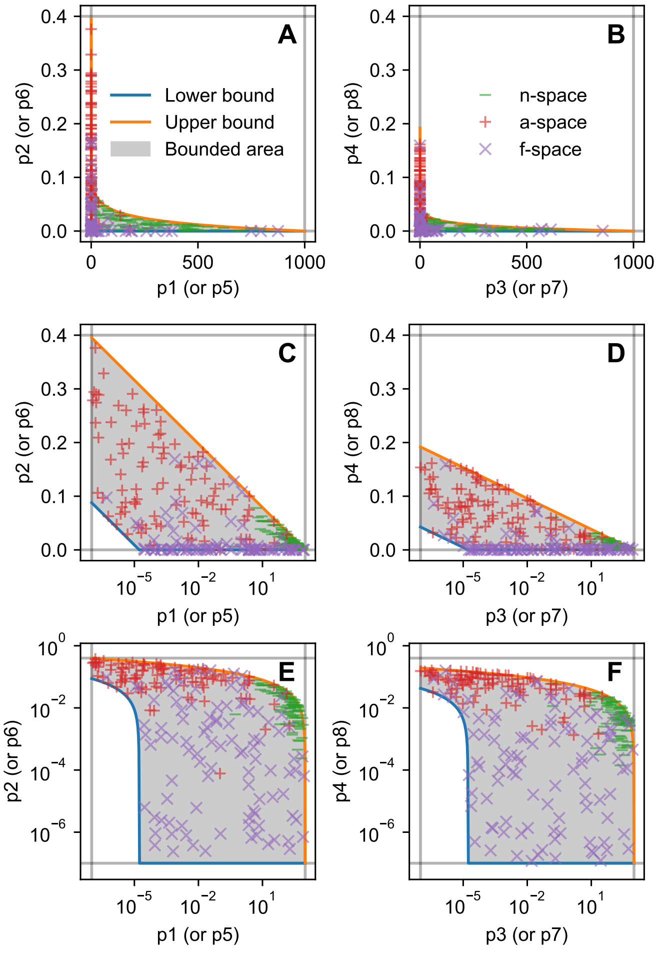

Figure S16: **Sampling from within the boundaries** A visualisation of the boundaries on the kinetic parameters is shown, drawn with two linear axes (*A* & *B*), a logarithmic x-axis (*C* & *D*), or two logarithmic axes (*E* & *B*). In each panel, the lower and upper bound for the parameters are shown as grey lines, the constraints imposed via the rate constant restrictions are shown in blue and orange, and the region fully within the boundaries is indicated with grey shading. Points sampled uniformly in the untransformed space (labelled the 'n-space') are shown, indicated with green '−' marks. The '+' marks indicate points sampled from a space where p_1, p_3, p_5, and p_7 are log-transformed (the 'a-space'), and points sampled uniformly from within a space where all parameters are log-transformed (the 'f-space') are shown as purple '×' marks.

S2.4.1 Effects of transformations on reliability

To investigate the effects of sampling initial points and searching in transformed spaces, we performed 50 Method 4 optimisations in different configurations on the synthetic data of Cell S, and calculated the percentage of results with an RMSE within 1% of the best result in each configuration. We used the notation *(search space, sampling space)* to denote each configuration, where 'search space' is the space presented to the optimiser, and 'sampling space' is the space from which we sampled uniformly to pick the starting point. The results, shown in Figure S17, suggest that changing the either the search-space or the choice of initial point in isolation only has a small effect on the reliability of the method. In contrast, changing both has a drastic influence, with the *(a, a)* configuration strongly outperforming the *(n, n)* and *(f, f)* configurations.

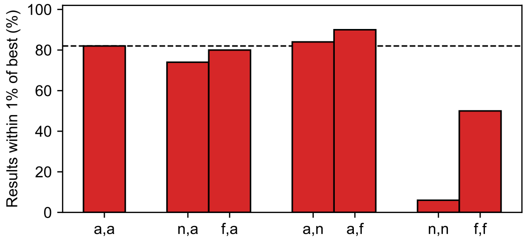

Figure S17: The percentage of Method 4 results with an RMSE within 1% of the best result found, for 50 repeats in different configurations, all for the synthetic data of Cell S. The first bar shows the baseline configuration *(a,a)* where searching and choosing initial points were performed in the a-space. The next two bars show the influence of changing the search space without changing the way initial points were chosen: *(n, a)* searching in the untransformed n-space, but choosing starting positions in a-space; and *(f, a)* searching in the p_{1-9} log-transformed f-space but sampling in a-space. Next, to see the effect of the starting position without changing the searching method we tested: *(a, n)* searching in a-space but choosing the first point from n-space; and *(a, f)* searching in a-space but choosing a point from a distribution uniform in f-space. And finally, we varied both simultaneously: *(n, n)* searching and sampling in n-space; and *(f, f)* searching and sampling in f-space.

Next, we repeated the experiment using Method 3. Here, the effects of changing the search space or initial point selection in isolation were larger. When changing both, the performance of the (f, f) configuration was much closer to the (a, a) configuration, but the (n, n) configuration still proved very unreliable.

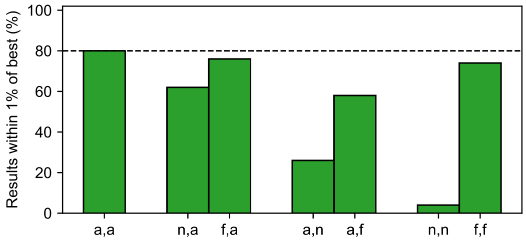

Figure S18: The percentage of Method 3 results with an RMSE within 1% of the best result found, for 50 repeats in different configurations, for the synthetic data of Cell S.

S2.4.2 Effects of transformations on performance

Next, we investigated the effects of parameter transformations on optimisation performance, by plotting the number of evaluations required in each optimisation for Method 3 and Method 4 in several configurations in Figure S19. These results show that (1) searching in a transformed space is more efficient than in an untransformed space; (2) the starting position has less influence on the performance, although choosing a distribution that favours points near the true solution reduces the number of evaluations needed; (3) that choosing bad starting points can negate the positive effects of using a transformed search space.

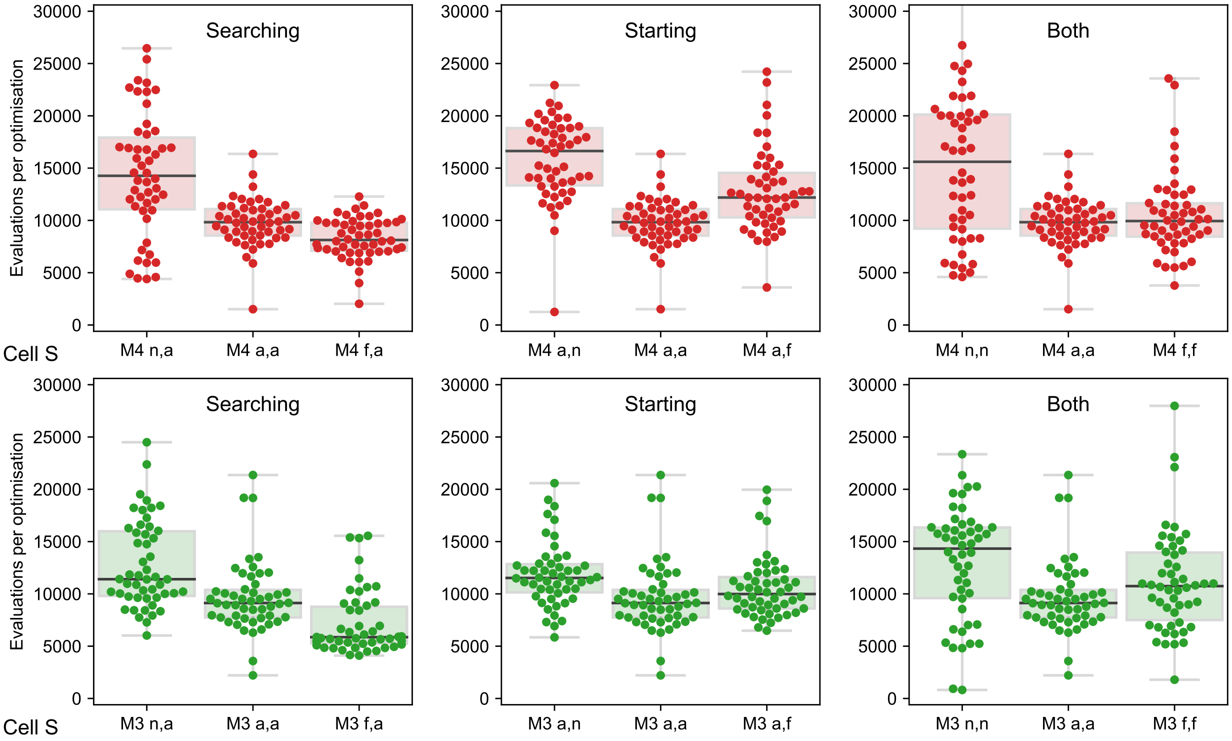

Figure S19: The number of evaluations per optimisation on the synthetic data of Cell S, for 50 optimisations in different configurations. (*Top*) Method 4 results for different search spaces (*left*), choosing starting points uniformly in different spaces (*centre*), or varying both (*right*). Note that the centre of each panel shows the same data (the baseline (a, a) configuration). (*Bottom*) A similar exploration for Method 3.

S2.4.3 Recommendations

In conclusion, our synthetic data study indicates that for transition rates of the form $a\exp(\pm bV)$ a parameter transform of '$\ln a$' and linear 'b' is a good choice for both reliability (reproducible best fits from different initial guesses) and performance (number of function evaluations to minimise with CMA-ES). These results may generalise to other model structures with voltage-dependent rates of the form $a\exp(\pm bV)$. The findings perhaps suggest rewriting the model equations in the form $exp(a' \pm bV)$ and removing the need for parameter transforms entirely by performing optimisation on a' and b.

S3 SUPPLEMENTAL RESULTS

S3.1 Obtained parameters

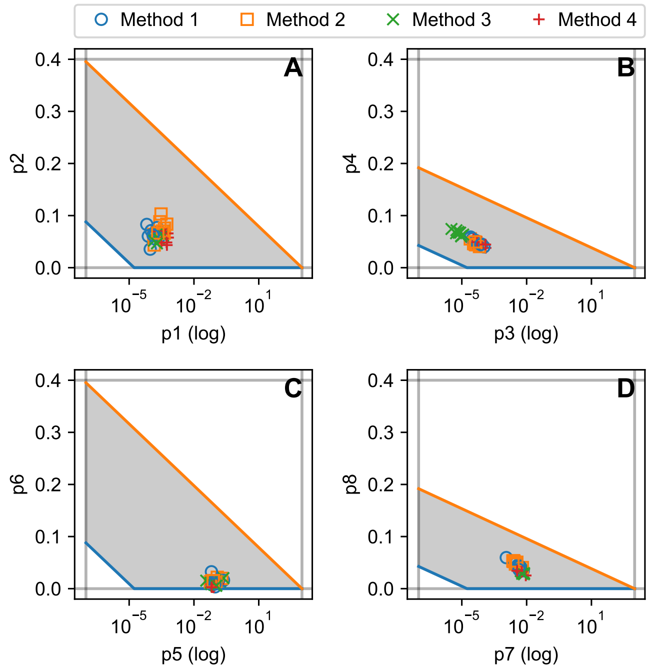

Figure S20: The best parameters returned by all four methods, for all nine cells. Only the eight kinetic parameters are shown. Parameters p_1 and p_2, shown in panel A, together determine the activation rate, while p_3 and p_4 in panel B determine deactivation. Similarly, inactivation and recovery are determined by the parameters in panels C and D respectively.

S3.2 Validation and cross-validation figures for all cells

Validation and cross-validation figures for all cells can be found at
https://github.com/CardiacModelling/FourWaysOfFitting.

S3.3 Relative RMSE tables for all cells

Cell 1	Method 1	Method 2	Method 3	Method 4
AP validation	1.53	1.35	1.00	1.36
Method 1 RMSE	1.14	1.00	3.21	1.59
Cross-validation M2	2.53	1.00	4.31	2.27
Cross-validation M3	1.99	1.75	1.00	1.92
Cross-validation M4	3.15	2.30	1.84	1.00

Cell 2	Method 1	Method 2	Method 3	Method 4
AP validation	2.26	3.00	1.00	1.19
Method 1 RMSE	1.12	1.00	2.34	1.60
Cross-validation M2	2.85	1.00	3.99	2.91
Cross-validation M3	2.33	2.46	1.00	2.07
Cross-validation M4	3.20	4.85	1.65	1.00

Cell 3	Method 1	Method 2	Method 3	Method 4
AP validation	1.14	2.10	1.00	1.37
Method 1 RMSE	1.00	1.07	2.85	1.94
Cross-validation M2	1.83	1.00	3.48	2.18
Cross-validation M3	1.29	1.46	1.00	1.54
Cross-validation M4	1.66	4.92	1.77	1.00

Cell 4	Method 1	Method 2	Method 3	Method 4
AP validation	2.03	1.87	1.22	1.00
Method 1 RMSE	1.01	1.00	2.94	2.64
Cross-validation M2	2.18	1.00	3.67	3.21
Cross-validation M3	1.35	1.77	1.00	1.73
Cross-validation M4	3.04	4.16	2.20	1.00

Cell 5	Method 1	Method 2	Method 3	Method 4
AP validation	1.82	4.25	1.29	1.00
Method 1 RMSE	1.00	1.11	4.64	1.99
Cross-validation M2	2.58	1.00	6.33	2.48
Cross-validation M3	2.46	3.88	1.00	2.13
Cross-validation M4	3.78	13.54	1.70	1.00

Cell 6	Method 1	Method 2	Method 3	Method 4
AP validation	2.92	1.00	1.07	1.32
Method 1 RMSE	2.33	1.00	3.74	2.06
Cross-validation M2	4.47	1.00	5.20	3.17
Cross-validation M3	2.41	1.79	1.00	2.29
Cross-validation M4	3.82	2.15	1.60	1.00

Cell 7	Method 1	Method 2	Method 3	Method 4
AP validation	2.04	1.85	1.12	1.00
Method 1 RMSE	1.00	1.21	5.85	3.03
Cross-validation M2	2.23	1.00	6.74	3.38
Cross-validation M3	1.36	1.52	1.00	1.47
Cross-validation M4	2.71	3.25	2.09	1.00

Cell 8	Method 1	Method 2	Method 3	Method 4
AP validation	2.09	1.56	1.00	1.09
Method 1 RMSE	1.35	1.00	4.79	2.28
Cross-validation M2	2.64	1.00	7.07	2.87
Cross-validation M3	1.61	1.50	1.00	1.47
Cross-validation M4	2.90	2.35	1.93	1.00

Cell 9	Method 1	Method 2	Method 3	Method 4
AP validation	1.42	1.70	1.00	1.25
Method 1 RMSE	1.00	1.01	2.21	1.81
Cross-validation M2	2.34	1.00	3.36	3.59
Cross-validation M3	1.57	1.61	1.00	1.52
Cross-validation M4	1.66	1.94	1.43	1.00

All cells	Method 1	Method 2	Method 3	Method 4
AP validation	1.7 (0.3)	1.9 (0.8)	1.0 (0.1)	1.1 (0.2)
Method 1 RMSE	1.2 (0.5)	1.0 (0.2)	3.3 (0.6)	1.9 (0.2)
Cross-validation M2	2.6 (0.7)	1.0 (0.1)	4.8 (1.0)	2.9 (0.4)
Cross-validation M3	1.7 (0.4)	1.8 (0.4)	1.0 (0.4)	1.7 (0.4)
Cross-validation M4	2.8 (0.9)	3.8 (1.8)	1.8 (0.5)	1.0 (0.3)

Figure S21: Relative RMSE for all cells.

S3.4 Performance

The experimental and computational effort that goes into each method is illustrated in Figure S22.

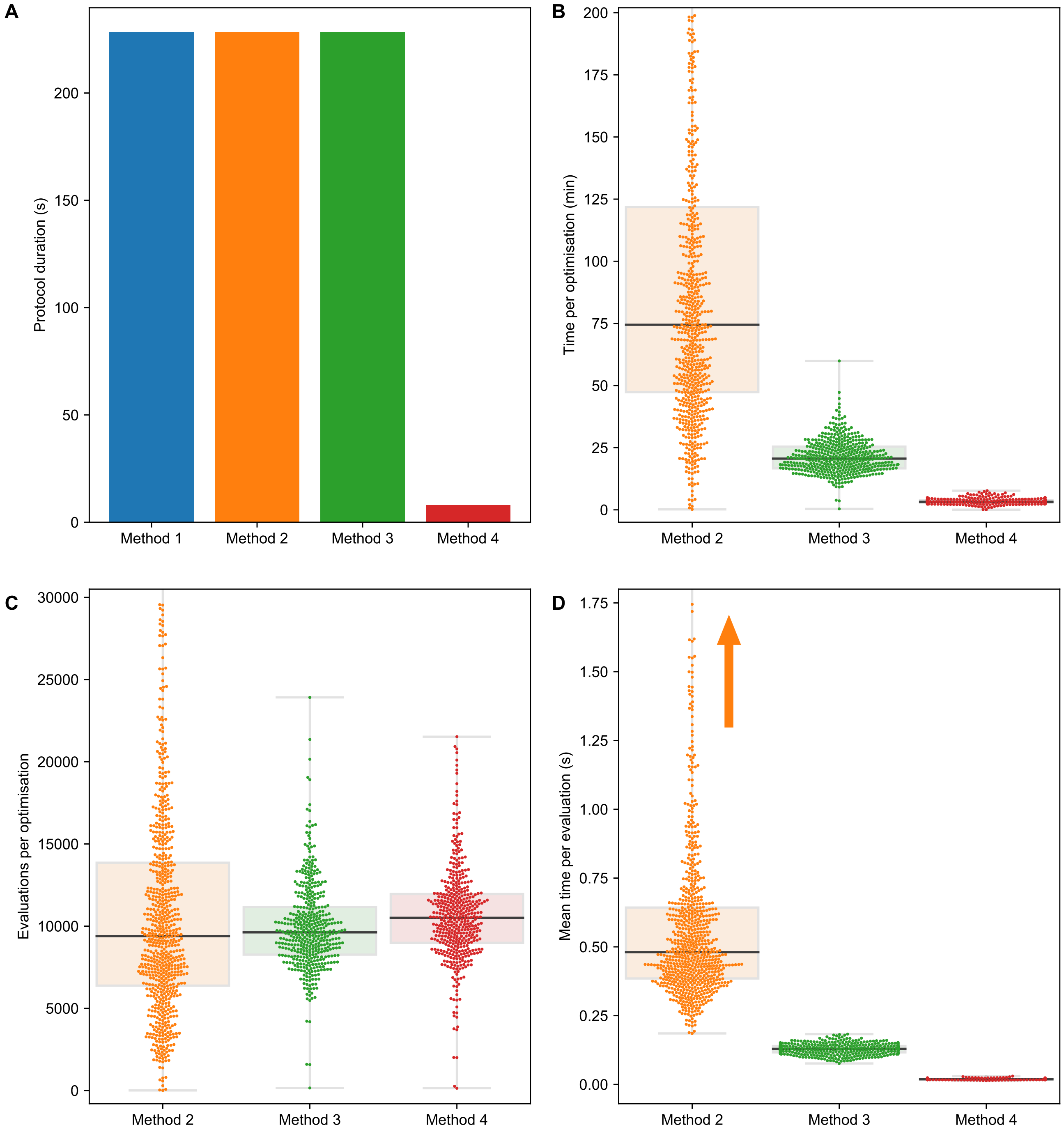

Figure S22: Experimental duration and optimisation duration. (*A*) The duration of the protocols needed for each method: 228s for methods 1–3 (which are based on Pr2–5), and 8 s for Method 4 (based on Pr7). (*B*) The time taken for a single optimisation, with 50 points shown per method per cell. (*C*) The number of evaluations per optimisation for methods 2–4. (*D*) The mean time per evaluation, calculated for 50 optimisations for each cell. Orange arrows indicate hidden outliers from Method 2.

Figure S22.A shows that the experiments required for Method 4 are considerably faster than those needed for methods 1–3, with only 8 seconds needed for Pr7, compared to 228 seconds for the Pr2–5 combination. The time for a single optimisation (one of the 50 repeats we ran per method) is shown for methods 2–4 in Figure S22.B. Method 1 is deterministic and runs almost instantaneously, so is omitted here. Method 2 takes far longer than the other methods, and Method 3 is slower than Method 4. Inspecting the number of evaluations (simulations) performed by each method, shown in Figure S22.C we see that Method 2 is much more similar to methods 3 and 4 in this respect. A large number of Method 2 optimisations terminated with a low number of evaluations, which indicates that they stopped exploring early, e.g. due to hitting a local minimum.

The time spent per optimisation (Figure S22.D) is largest in Method 2, followed by Method 3, followed by Method 4. Figure S23 shows this difference is due to the time per evaluation, rather than the number of evaluations. Method 3 evaluations take longer than Method 4 evaluations, as they require the simulation of multiple, long protocols (see Figure S22.A). The time per evaluation for Method 2 is larger still, since Method 2 not only runs a simulation every evaluation, but also needs to post-process the results to extract time constants and steady states.

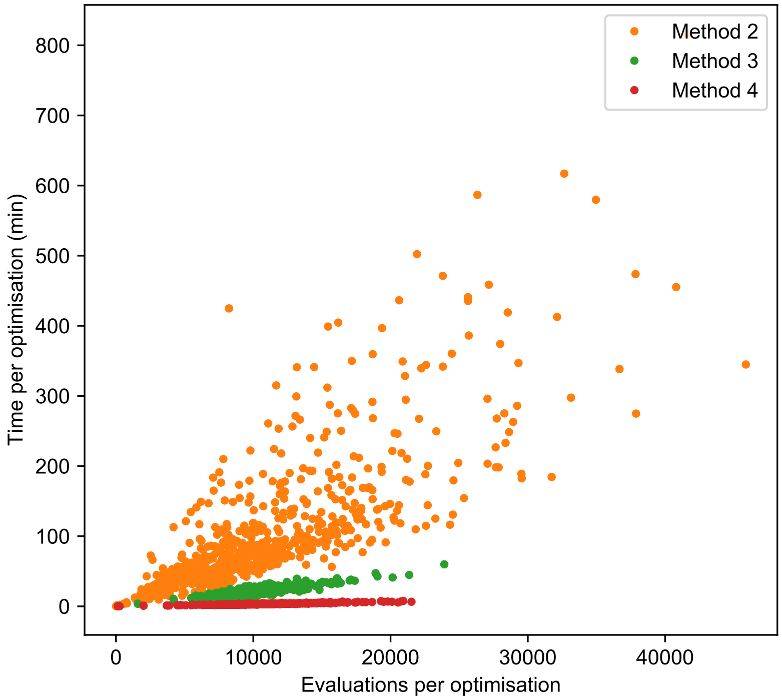

Figure S23: Time per optimisation versus number of evaluations per optimisation.

S3.5 Cross-sections of the optimisation surfaces

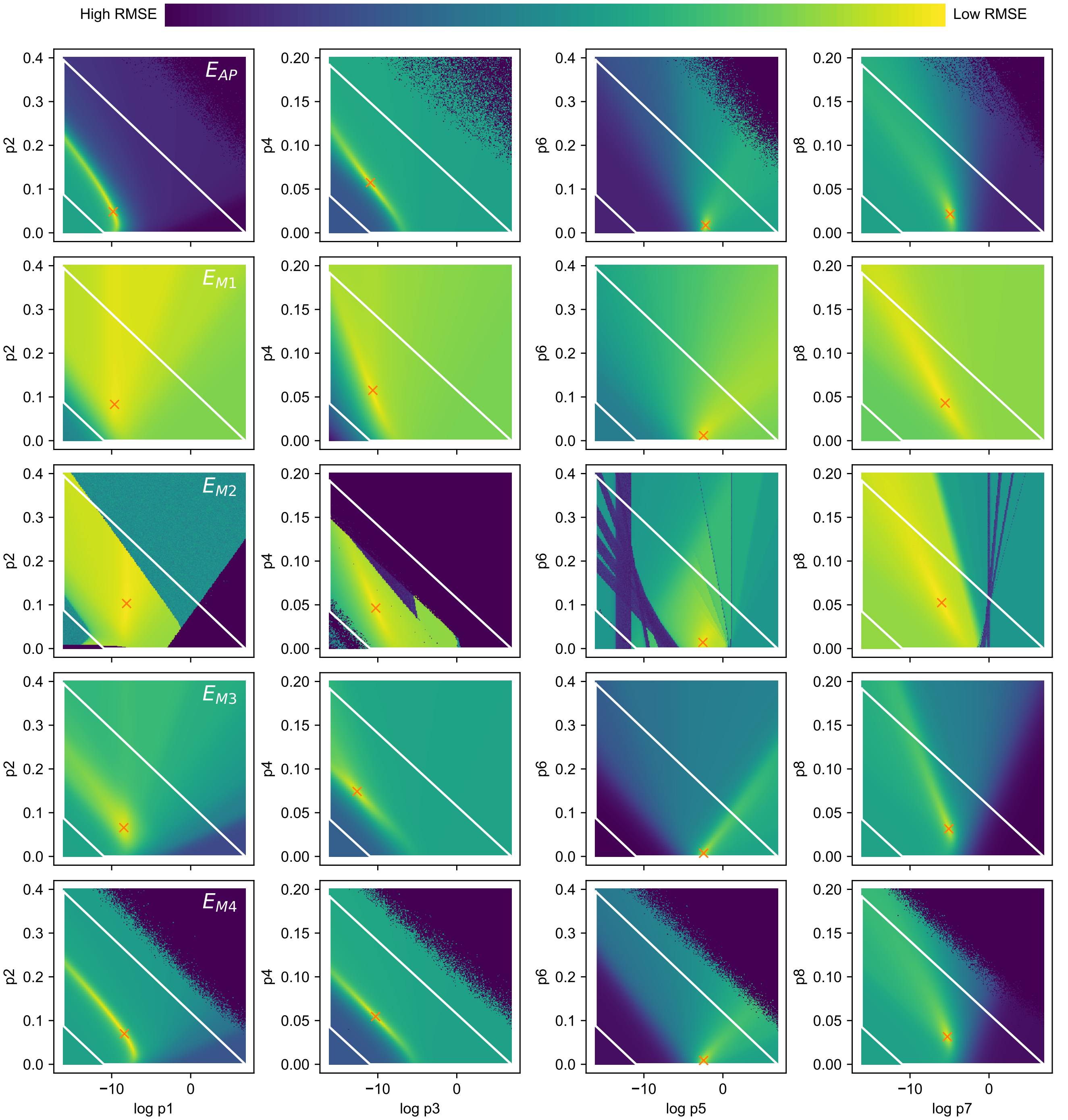

Figure S24: Cross-sections of all optimisation criteria for Cell #5, for E_{AP} (*Top*), and E_{M1} to E_{M4}. The surfaces were drawn by performing a brute-force mapping (256x256 evaluations) around a fixed point. For E_{AP} this fixed point was chosen by first running an optimisation to find its optimum. For E_{M1} to E_{M4} the fixed point was the result returned by methods 1–4. Note that for E_{M1} this point does not correspond exactly to E_{M1}'s minimum.

S3.6 Method 1b: Minimising E_{M1}

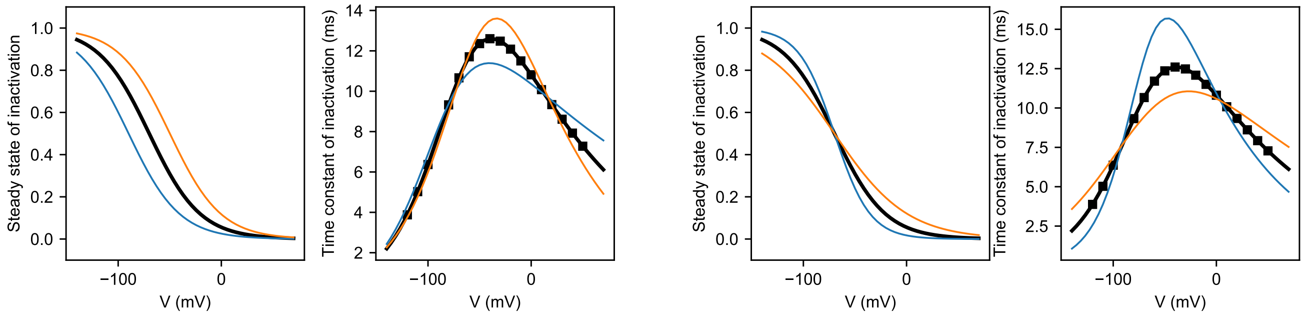

Figure S25: In Method 1, the steady-state approximations are used in deriving the approximations of the time constants. The time constants are relatively robust against shifts in the midpoints of (in)activation, but react more strongly to changes in the steady state slopes. For Method 1, this implies that a small error in estimating the slope (for example due to having points near the reversal potential) can cause a large error in the time constants. For Method 2, it shows that a better fit on the time constants can be obtained by slightly tweaking the steady state curves.

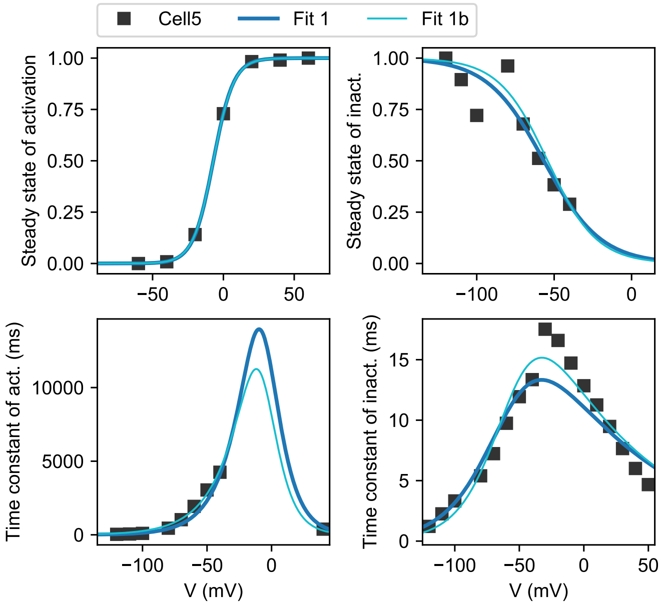

Figure S26: Minimising E_{M1} directly allows for a trade-off between goodness-of-fit in the steady states and the time constants. This results in improved time-constant fits, without much change to the steady-state fits. Data is shown for Cell #5.

Cell 5	Method 1	Method 2	Method 3	Method 4
AP validation	1.82	4.25	1.29	1.00
Method 1 RMSE	1.00	1.11	4.64	1.99
Cross-validation M2	2.58	1.00	6.33	2.48
Cross-validation M3	2.46	3.88	1.00	2.13
Cross-validation M4	3.78	13.54	1.70	1.00

Cell 5	Method 1b	Method 2	Method 3	Method 4
AP validation	1.85	4.25	1.29	1.00
Method 1 RMSE	1.00	1.43	5.96	2.56
Cross-validation M2	1.98	1.00	6.33	2.48
Cross-validation M3	3.46	3.88	1.00	2.13
Cross-validation M4	6.84	13.54	1.70	1.00

Figure S27: Using Method 1b (*Right*) leads to E1 RMSEs that outperform Method 1 (*Left*). However, this improvement does not necessarily translate to better predictions, as the fundamental idea — that the model equations should be overlaid on their experimental approximates — is still flawed. Data is shown for Cell #5.

S3.7 Method 2b: Minimising E_{M2} starting from Method 1 result

A Method 2 variant ("Method 2b") can be created by adapting Method 2 to only perform a single run, starting from the parameters returned by Method 1. In the experiments we ran, this gave very similar results to Method 2, but at a lower computational costs. However, these results will only generalise if the function E_{M2} is smooth and easy to navigate between the Method 1 and Method 2 results.

Table S4: Method 2 and Method 2b results

Cell	E_{M2} Method 2	E_{M2} Method 2b
1	0.1842753785	0.1900501636
2	0.1807762308	0.1827848996
3	0.2155275628	0.2155275628
4	0.1648576405	0.1648576405
5	0.1583982474	0.1584057577
6	0.1664606846	0.1669911494
7	0.1335628150	0.1335628150
8	0.1654924563	0.1654924564
9	0.1846339139	0.1880672499

S3.8 Method 3b: Minimising E_{M2} starting from Method 1 result

A similar adaptation can be used to create a Method 3 variant, "Method 3b".

Table S5: Method 3 and Method 3b results

Cell	E_{M3} Method 3	E_{M3} Method 3b
1	0.0448121214	0.0448121214
2	0.0479263275	0.0479263275
3	0.0613629021	0.0613629021
4	0.0801896081	0.0801896081
5	0.0394435210	0.0394435210
6	0.0666019884	0.0666019884
7	0.1164911334	0.1164911334
8	0.1005265480	0.1005265480
9	0.0683642823	0.0683642823

REFERENCES

1. Beattie, K. A., A. P. Hill, R. Bardenet, Y. Cui, J. I. Vandenberg, D. J. Gavaghan, T. P. de Boer, and G. R. Mirams, 2018. Sinusoidal Voltage Protocols For Rapid Characterization Of Ion Channel Kinetics. *The Journal of Physiology* 596:1813–1828.

2. Clerx, M., 2018. Personalisation of cellular electrophysiology models: utopia? *In* Computing in Cardiology. CINC, volume 45.

3. Hodgkin, A. L., and A. F. Huxley, 1952. A quantitative description of membrane current and its application to conduction and excitation in nerve. *The Journal of Physiology* 117:500–544.

4. Vandenberg, J. I., M. D. Perry, M. J. Perrin, S. A. Mann, Y. Ke, and A. P. Hill, 2012. hERG K+ channels: structure, function, and clinical significance. *Physiological Reviews* 92:1393–1478.

5. Sakakibara, Y., J. A. Wasserstrom, T. Furukawa, H. Jia, C. E. Arentzen, R. S. Hartz, and D. H. Singer, 1992. Characterization of the sodium current in single human atrial myocytes. *Circulation Research* 71:535–546.

6. Beaumont, J., F. Roberge, and L. Leon, 1993. On the interpretation of voltage-clamp data using the Hodgkin-Huxley model. *Mathematical biosciences* 115:65–101.

7. Willms, A. R., D. J. Baro, R. M. Harris-Warrick, and J. Guckenheimer, 1999. An improved parameter estimation method for Hodgkin-Huxley models. *Journal of Computational Neuroscience* 6:145–168.

8. Lee, J., B. Smaill, and N. Smith, 2006. Hodgkin–Huxley type ion channel characterization: an improved method of voltage clamp experiment parameter estimation. *Journal of Theoretical Biology* 242:123–134.
